# Synthesis and optical resolution of 4,5-diaminohomoadamantane: a promising scaffold for chiral ligands and bioactive compounds

**DOI:** 10.3762/bjoc.22.80

**Published:** 2026-07-01

**Authors:** Polina Anatolyevna Man’kova, Vadim Andreevich Shiryaev, Olga S Podlipnova, Marat M Khisyamov, Dmitry Sergeevich Nikerov, Alexander Nikolaevich Reznikov, Yuri Nikolaevich Klimochkin

**Affiliations:** 1 Department of Organic Chemistry Samara State Technical University, Molodogvardeyskaya st. 244, 443100 Samara, Russia

**Keywords:** asymmetric Henry reaction, asymmetric Michael reaction, diaminohomoadamantane, enantiodivergent effect, resolution of racemate

## Abstract

Vicinal diamines based on a rigid polycyclic framework such as homoadamantane remain underexplored. We anticipated that the unique steric and lipophilic properties of chiral *trans*-4,5-diaminohomoadamantane could provide the necessary stereoinduction in metal-catalyzed asymmetric reactions. In addition, such structures may serve as a novel scaffold for bioactive compounds. Herein, we report a synthetic approach to this previously inaccessible chiral scaffold. 4,5-Diaminohomoadamantane was prepared as a mixture of *cis*- and *trans*-isomers by reduction of the corresponding vicinal azidoxime with LiAlH_4_. In contrast, the *trans*-isomer was selectively obtained via ring-opening of an *N*-Tf-protected aziridine. The racemic *trans*-4,5-diaminohomoadamantane was resolved with dibenzoyl-ʟ-tartaric acid. The absolute (4*R*,5*R*)-configuration was proposed on the basis of TDDFT calculations of the specific optical rotation using the CAM-B3LYP functional and the 6-311G++(2d,2p) basis set with solvation by CH_2_Cl_2_ in the SMD model on the base of conformational analysis. Catalytic systems based on (4*R*,5*R*)-4,5-diaminohomoadamantane derivatives exhibited low to moderate asymmetric induction in Henry and Michael reactions. These results suggest that further molecular design of homoadamantane-based chiral *N*,*N*-ligands is promising.

## Introduction

**Figure 1:**
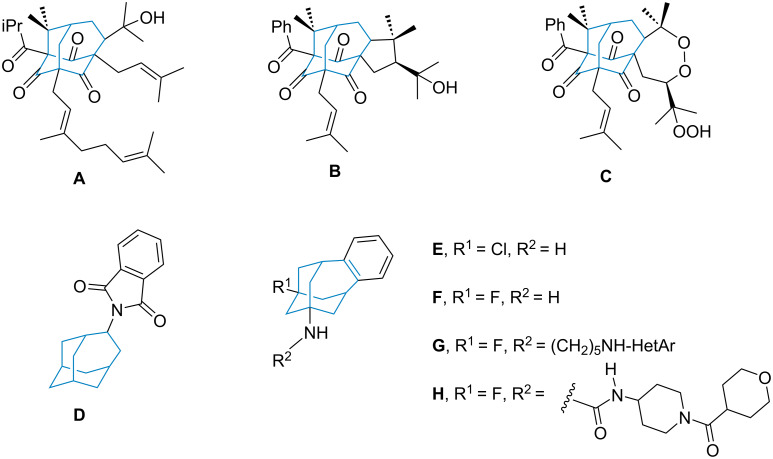
Examples of bioactive homoadamantanes.

The chemistry of organic polycyclic cage compounds has been of interest to organic chemists for many years. Such a simple and symmetric structure as adamantane is widely used in pharmaceutics to obtain drugs with a wide spectrum of action, as well as to improve the properties of drugs currently in use [1–7]. The chemistry of adamantane has been extensively studied [8–16], while the properties of homoadamantane, its closest homologue, have not been studied so thoroughly. Chemical transformations of homoadamantane both at the bridgehead [17–23] and at the C-4 positions [24–28] are described in the literature. However, compounds of the homoadamantane family with substituents at the C-3 and C-4 [29–38] or at the C-4 and C-5 [39–53] positions have recently attracted more attention. At present, a few examples of homoadamantane derivatives showing pronounced biological activity are known. More than 40 homoadamantane polyprenylated acylphloroglucinols have been isolated from *Hypericum* plants [54–56]. Among them, hyperacmosins **A** and **B** showed a protective effect in paracetamol-induced cell damage [57], and activity against *Staphylococcus aureus* was noted for peroxysampsone A **С** [58]. Basarić and co-workers showed that the replacement of 2-substituted adamantane by a homoadamantane skeleton led to enhanced antiproliferative activity (compound **D**) [59]. Compound **E** showed efficacy against *Staphylococcus aureus* [60]. Fluorobenzohomoadamantanamine **F** proved to be a potent NMDA receptor antagonist with efficacy very close to memantine [61] and compound **G** is approximately two times more potent as an NMDA antagonist than compound **F** [62]. The benzohomoadamantane-based derivative **H** showed high analgesic activity in a mouse model of cystitis (Figure 1) [63].

**Figure 2:**
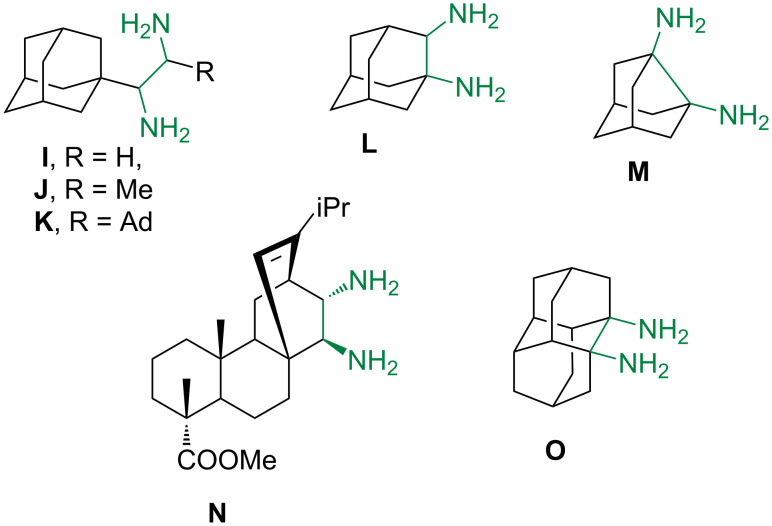
Examples of cage vicinal diamines.

Vicinal diamines are of interest to chemists not only as precursors for the synthesis of heterocyclic compounds, but also because of their wide range of practical applications. Structures with a 1,2-diamine fragment are widely used in clinical practice [64–66], as well as auxiliaries and ligands in important asymmetric transformations [67–70]. Bi- and polycyclic vicinal diamines can also be considered as potential ligands for asymmetric catalysis [71–74], but little attention has been paid to methods for obtaining compounds of this type. Vicinal diamines with an adamantyl substituent **I**–**K** have already been obtained [75–78] and 1,2-diaminoadamantane **L** has been synthesized by different methods [79–81]. For 1,2-diaminonoradamantane **M** there are even fewer data [82]. Polycyclic vicinal diamines **N** and **O** have been prepared recently (Figure 2) [69,83–84].

## Results and Discussion

**Scheme 1:**
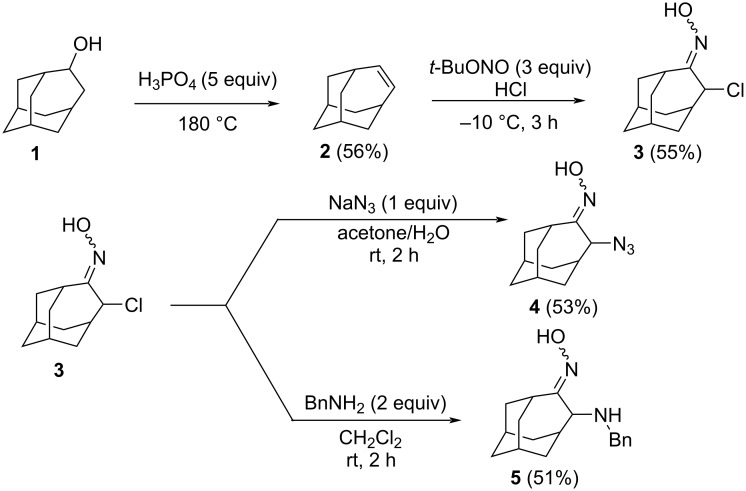
Synthetic route to homoadamantane oximes **3**–**5**.

Synthetic approaches to cage vicinal diamines are actively studied, as their rigid cage framework makes them promising ligands for asymmetric catalysis. In continuation of the theme of our studies [75,77,81], we decided to focus on new approaches to the synthesis of 4,5-diaminohomoadamantane, its chiral resolution, and to test its applicability as chirality-inducing ligand in selected model reactions. We commenced the study using a route for the synthesis of 4,5-diaminohomoadamantane from azidoxime **4**. Initially, homoadamantan-4-ene (**2**) was obtained from homoadamantan-4-ol (**1**). In 1969, a method for the preparation of alkene **2** from homoadamant-4-yl tosylate was proposed [85]. However, the formation of methyl tosylate and the need for purification of the product reduces its usefulness. We herein report an improved method for the synthesis of compound **2** from alcohol **1** using phosphoric acid, affording the target compound in high yield and purity. In the next step, chloronitrosation of alkene **2** led to α-chloroxime **3** in moderate yield. Substitution of chlorine in **3** with azido and benzylamino groups was then carried out to give azidoxime **4** and aminoxime **5** (Scheme 1).

**Scheme 2:**
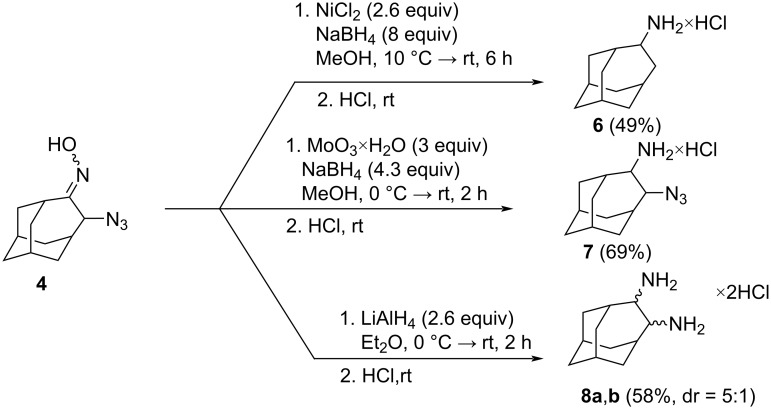
Reduction of azidoxime **4**.

The reduction of azidoxime **4** was carried out using various systems in order to obtain *trans*-4,5-diaminohomoadamantane. Thus, treatment of compound **4** with NaBH_4_ in the presence of nickel(II) chloride [86] resulted in the formation of 4-aminohomoadamantane (**6**). The NaBH_4_/MoO_3_ system [81] led to the selective reduction of only the hydroxyimino group, affording azidoamine **7**. The reduction of azidoxime **4** using LiAlH_4_ [75,87] allowed total reduction of both the oxyimino and azido groups, resulting in the formation of isomeric diamines **8a**,**b** (Scheme 2).

**Scheme 3:**
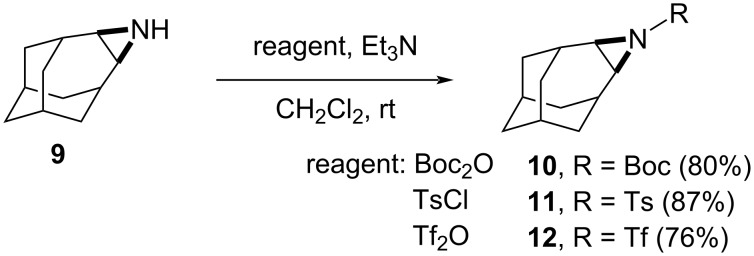
Synthesis of *N*-substituted aziridines **10**–**12**.

Since 4,5-diaminohomoadamantane was isolated as a mixture of *trans*- and *cis*-isomers **8a**,**b**, we decided to synthesize *trans*-4,5-diaminohomoadamantane through ring opening of aziridine **9**. This compound was obtained from homoadamantanone oxime by reduction with LiAlH_4_ [41]. Since the aziridine ring-opening reaction usually proceeds easily when the aziridine bears an electron-withdrawing group at the *N*-atom, we synthesized *N*-substituted aziridines **10**–**12** by reactions of **9** with Boc_2_O, TsCl, and Tf_2_O, respectively (Scheme 3).

**Scheme 4:**
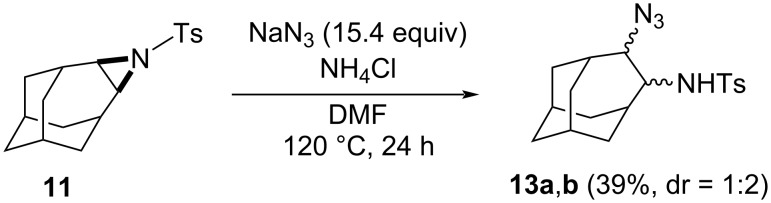
Aziridine **11** ring-opening reaction with NaN_3_.

Compounds **10**–**12** were studied in the aziridine ring-opening reaction with sodium azide. Typically, *N*-Boc-substituted aziridines easily give the corresponding ring-opened products [77,88–89], however in our case only the starting compound **10** was isolated. In the reaction of tosylated compound **11** with sodium azide in the presence of ammonium chloride, *trans*- and *cis*-diastereomers of **13a**,**b** were obtained (Scheme 4).

**Scheme 5:**
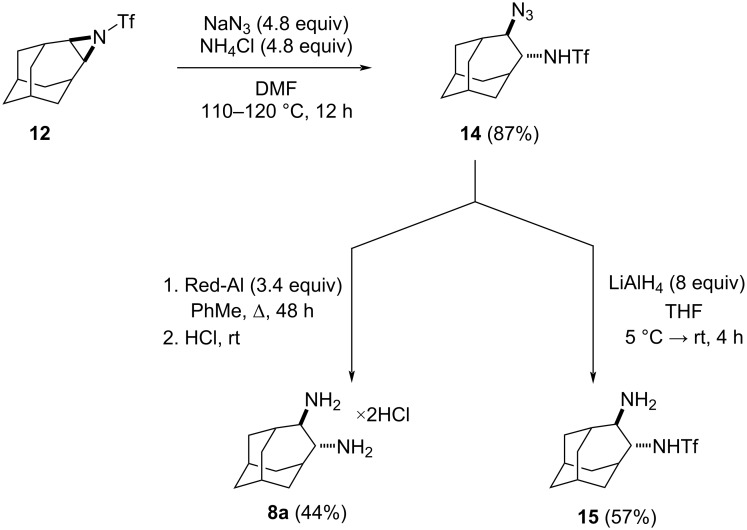
Aziridine **12** ring-opening and reduction.

In order to find a more selective method for the synthesis of *trans*-4,5-diaminohomoadamantane (**8a**), aziridine **12** was studied. When the tosyl group was replaced with a triflic group (**12**), only the *trans*-diastereomer **14** was formed in the reaction with NaN_3_ and NH_4_Cl. Upon reduction of the azido group with LiAlH_4_, the *trans*-isomer **15** was isolated. However, the removal of the protecting group in **15** by the known method [90] (sequential action of chloroacetonitrile and Cs_2_CO_3_) was unsuccessful. Therefore, we decided to use Red-Al [91–92] for simultaneous reduction and deprotection of **14** yielding only the *trans*-diastereomer **8a** (Scheme 5).

**Scheme 6:**
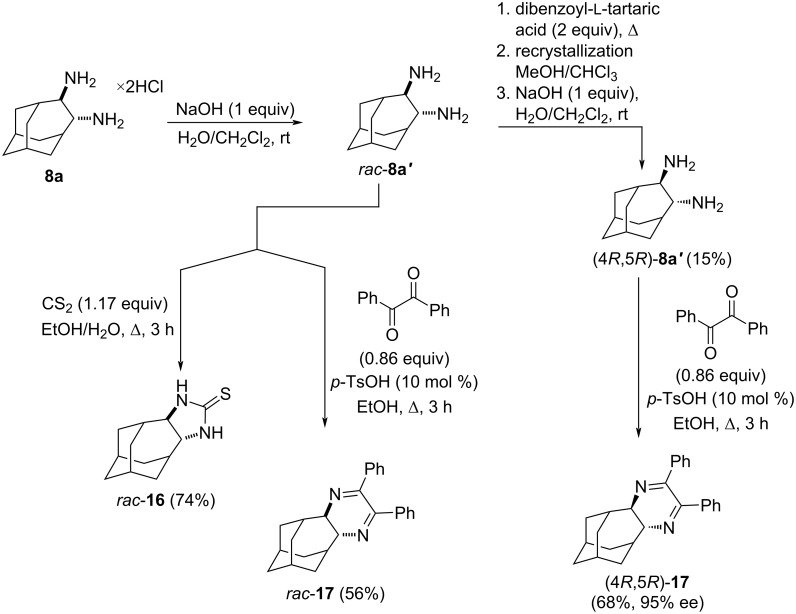
Resolution of racemic diamine **8a′**.

At the next stage of the research, we attempted to resolve racemic *trans*-4,5-diaminohomoadamantane (**8a′**) with the use of ʟ-tartaric, ʟ-malic, (*R*)-mandelic and dibenzoyl-ʟ-tartaric acids. The diastereomeric salts derived from the corresponding acids and 4,5-diaminohomoadamantane (**8a′**) in both 2:1 and 1:1 ratios were studied. Successful resolution was achieved by triple crystallization of the bis(dibenzoyl)-ʟ-tartrate from a MeOH/CHCl_3_ mixture (Scheme 6).

The attempted determination of the enantiomeric ratio by chiral HPLC of the free amine failed because no optimal analysis conditions could be identified. Therefore, a preliminary derivatization of diamine **8a′** was required, which was carried out by reactions with carbon disulfide [75] or benzil [77].

**Figure 3:**
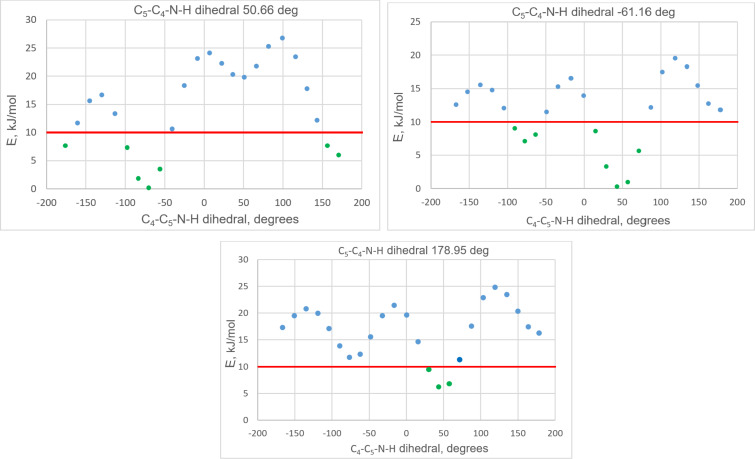
The results of C_4_–C_5_–N–H dihedrals scan for staggered conformations with C_5_–C_4_–N–H dihedrals 50.66, −61.16, and 178.95 degrees. Red lines in the graphics represent the energy of 10 kJ/mol. The scans were performed using HF/6-31G(d) method.

However, HPLC analysis was only successful for racemic 2,3-dihydropyrazine **17** (Scheme 6). For (4*R*,5*R*)-2,3-dihydropyrazine (4*R*,5*R*)-**17** derived from diamine (4*R*,5*R*)-**8a'**, the enantiomeric excess was determined to be 95%. Multiple attempts to grow a crystal suitable for X-ray analysis were unsuccessful; therefore, the absolute configuration of (4*R*,5*R*)-**8a'** was proposed on the basis of quantum mechanical calculations. To date, a number of successful determinations of the absolute configuration for both conformationally flexible and rigid molecules have been performed by comparison of experimental and calculated specific rotation values [93–101]. The main difficulty of such a procedure is taking into account all possible conformers of a molecule, especially a flexible one, since specific rotation depends fundamentally on dihedral angles along bonds adjacent to the asymmetric atom. Therefore, the calculation of the specific rotation of rigid molecules is significantly easier since fewer conformers must be taken into account. Recently, it was shown, that calculated optical rotation values may be significantly affected not only by conformers but also by conformations that are close to them in energy [102–103]. Therefore, to calculate the specific rotation of (4*R*,5*R*)-**8a′** we applied a methodology that considers optical rotation of conformations that differ from the stable ones by no more than 10 kJ/mol [103]. All calculations were performed with the Gaussian 09 software [104]. Optimization of the molecule’s geometry by DFT was performed using the CAM-B3LYP functional and the 6-311G++(2d,2p) basis set with solvation by CH_2_Cl_2_ in the SMD model. The dihedral scan for the C_4_–NH_2_ and C_5_–NH_2_ bonds was performed using the HF method in the 6-31G+(d) basis set. The dihedral angle for C_5_–C_4_–N–H was set to 50.66, −61.16, and 178.95 degrees that corresponds to staggered conformations and for each of them the dihedral angle for C_4_–C_5_–N–H was scanned in 15 degrees increments, starting from 171.48 degree. The results of scans are represented in Figure 3.

All in all, 18 conformations, that have energies within 10 kJ/mol from the most stable conformer, were chosen for further computation. The geometry optimization and optical rotation calculations of the chosen conformations were performed using DFT method with the CAM-B3LYP functional and the 6-311G++(2d,2p) basis set with solvation by CH_2_Cl_2_ in SMD model. The distribution of conformations was calculated on the basis of their relative energy with respect to the minimum energy using the Boltzmann law. The overall specific rotation was calculated through weight averaging of all chosen conformations. The results of the specific rotation calculation for six wavelengths, reported in Table 1, agree well with the experimental values of specific rotation, what allows to assign the (4*R,*5*R*)-configuration to compound **8a′**.

**Table 1:** Predicted and experimental specific rotations for (*4R,5R*)-**8a′**.

Wavelength, nm	Average calсd 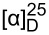 , CAM-B3LYP/6-311G++ (2d,2p)/SMD(CH_2_Cl_2_)	Experimental 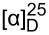

365	71.89	100.10
405	41.66	60.44
436	36.59	36.78
546	17.98	20.98
589.3	14.48	13.13
633	11.92	8.41

**Scheme 7:**
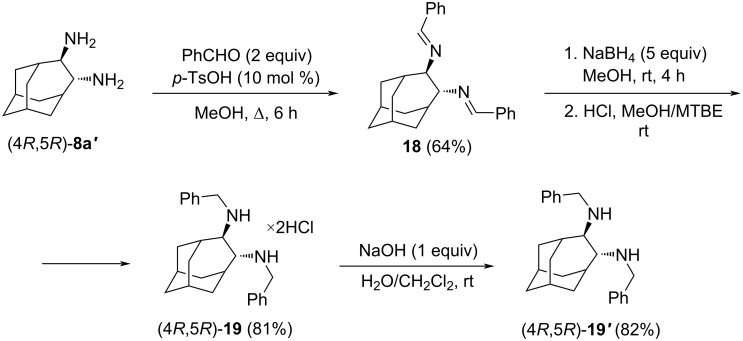
Synthesis of ligands **18** and **19′**.

Since the chiral resolution of *trans*-4,5-diaminohomoadamantane was successful, we decided to study metal complexes of ligands based on (4*R*,5*R*)-**8a′** in model reactions. Ligand **18** was obtained by the reaction of diamine (4*R*,5*R*)-**8a'** with benzaldehyde and the *N*,*N*’-dibenzyl-substituted ligand (4*R*,5*R*)-**19’** was synthesized by reduction of diimine **18** (Scheme 7).

**Scheme 8:**
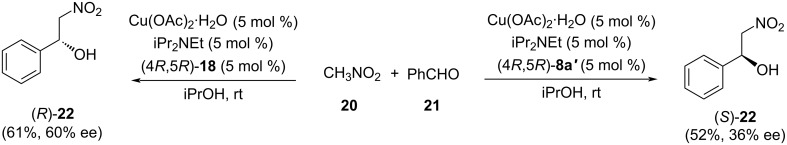
Enantiodivergent Henry reaction when using ligands **8a′** and **18**.

Ligands (4*R*,5*R*)-**8a′** and **18** were studied as chiral inducers in a Henry reaction. The reaction of benzaldehyde with nitromethane was carried out in iPrOH in the presence of copper(II) acetate hydrate, ligand **8a′** or **18,** and diisopropylethylamine (5 mol % each) (Scheme 8).

When diamine (4*R*,5*R*)-**8a′** was used, the reaction mixture was enriched in the (*S*)-isomer (36% ee), while diimine ligand **18** promoted the formation of mainly the (*R*)-isomer (60% ee). The formation of products with different configurations when using ligands of similar structure indicates an enantiodivergent effect [105].

**Scheme 9:**
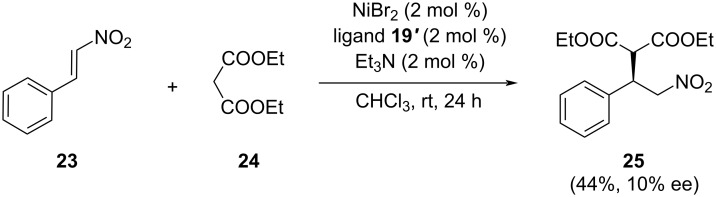
Catalysis of the Michael reaction using the complex (4*R*,5*R*)-**19′**−NiBr_2_.

The ligand **19′** was studied in a model Michael reaction. The reaction of nitrostyrene with diethyl malonate was conducted in chloroform in the presence of ligand **19′**, NiBr_2_, and NEt_3_ (2 mol % each). This reaction does not proceed in the absence of triethylamine, as evidenced by the quantitative recovery of the starting materials (Scheme 9).

A slight enantiomeric enrichment in the (*S*)-enantiomer (10% ee) was observed with the *N*,*N*′-dibenzyl-substituted diamine **19′** based complex.

## Conclusion

In conclusion, a new route for the synthesis of *trans*-4,5-diaminohomoadamantane and its subsequent optical resolution has been proposed. This compound is a valuable chiral scaffold for the construction of bioactive molecules and chiral ligands. In this work, chiral imine and amine ligands based on *trans*-4,5-diaminohomoadamantane were obtained. Catalytic systems based on them have been studied in the asymmetric Michael and Henry reactions. Low enantioselectivity (up to 60% ee) was observed, but an enantiodivergent effect was noted in the Henry reaction.

## Supporting Information

File 1 Experimental section.

## Data Availability

All data that supports the findings of this study is available in the published article and/or the supporting information of this article.
