# Supplementary material for: Synthesis and optical resolution of 4,5-diaminohomoadamantane: a promising scaffold for chiral ligands and bioactive compounds
Source: Beilstein J Org Chem. 2026 Jul 1;22:1013–22. doi: 10.3762/bjoc.22.80 (PMC13338599; doi:10.3762/bjoc.22.80)
Supplement: File 1 — Experimental section. [file Beilstein_J_Org_Chem-22-1013-s001.pdf]

BEILSTEIN JOURNAL OF ORGANIC CHEMISTRY

Supporting Information

for

Synthesis and optical resolution of 4,5-diaminohomoadamantane: a promising scaffold for chiral ligands and bioactive compounds

Polina A. Man’kova, Vadim A. Shiryaev, Olga S. Podlipnova, Marat M. Khisyamov, Dmitry S. Nikerov, Alexander N. Reznikov and Yuri N. Klimochkin

Beilstein J. Org. Chem. **2026,** *22,* 1013–1022. doi:10.3762/bjoc.22.80

Experimental section

License and Terms: This is a supporting information file under the terms of the Creative Commons Attribution License (https://creativecommons.org/licenses/by/4.0). Please note that the reuse, redistribution and reproduction in particular requires that the author(s) and source are credited and that individual graphics may be subject to special legal provisions.
The license is subject to the *Beilstein Journal of Organic Chemistry* terms and conditions: (https://www.beilstein-journals.org/bjoc/terms)

Table of contents

1. General information S2

2. Synthetic procedures S2

3. ^{1}H NMR and ^{13}C NMR spectra for synthesized compounds S15

3.1. NMR spectra of 5-chlorotricyclo[4.3.1.13,8]undecane-4-one oxime (**3**) S15

3.2. NMR spectra of 5-azidotricyclo[4.3.1.$1^{3,8}$]undecane-4-one oxime (**4**) S17

3.3. NMR spectra of 5-(benzylamino)tricyclo[4.3.1.$1^{3,8}$]undecan-4-one oxime (**5**) S19

3.4. NMR spectra of tricyclo[4.3.1.$1^{3,8}$]undecane-4-amine hydrochloride (**6**) S21

3.5. NMR spectra of 5-azidotricyclo[4.3.1.$1^{3,8}$]undecan-4-amine hydrochloride (**7**) S23

3.6. NMR spectra of *trans*/*cis*-4,5-diaminohomoadamantane dihydrochloride **8a,b** S25

3.7. NMR spectra of *trans*-4,5-diaminohomoadamantane dihydrochloride (**8a**) S27

3.8. NMR spectra of *tert*-butyl 5-azatetracyclo[5.3.1.$1^{3,9}$.$0^{4,6}$]dodecane-5-carboxylate (**10**) S29

3.9. NMR spectra of *cis*-5-*p*-tosyl-5-azatetracyclo[5.3.1.$1^{3,9}$.$0^{4,6}$]dodecane (**11**) S31

3.10. NMR spectra of *cis*-5-((trifluoromethyl)sulfonyl)-5-azatetracyclo[5.3.1.$1^{3,9}$.$0^{4,6}$]dodecane (**12**) S33

3.11. NMR spectra of *trans*/*cis*-*N*-(5-azidotricyclo[4.3.1.$1^{3,8}$]undecan-4-yl)-4-methylbenzenesulfonamide (**13a,b**) S35

3.12. NMR spectra of *trans*-*N*-(5-azidotricyclo[4.3.1.$1^{3,8}$]undecan-4-yl)-1,1,1-trifluoromethanesulfonamide (**14**) S37

3.13. NMR spectra of *trans*-*N*-(5-aminotricyclo[4.3.1.$1^{3,8}$]undecan-4-yl)-1,1,1-trifluoromethanesulfonamide (**15**) S39

3.14. NMR spectra of *trans*-decahydro-4,8:6,10-dimethanocyclonona[*d*]imidazole-2(1*H*)-thione (**16**) S41

3.15. NMR spectra of *rac*-2,3-diphenyl-4*a*,6,7,8,9,10,11,11*a*-octahydro-5*H*-5,9:7,11-dimethanocyclonona[*b*]pyrazine (**17**) S43

3.16. NMR spectra of (4*R*,5*R*)-*N*,*N*´-(tricyclo[4.3.1.$1^{3,8}$]undecane-4,5-diyl)bis(1-phenylmethanimine) (**18**) S45

4. Computational details of specific rotation determination of (4*R*,5*R*)-**8a´** S47

References S90

1. General information

IR spectra were recorded on a Shimadzu IR Affinity-1 spectrophotometer with single-reflection ATR accessory. ^{1}H and ^{13}C NMR spectra were recorded with a JEOL JNM-ECX400 spectrometer at 399.78 and 100.53 MHz in $CDCl_3$ and DMSO-d_6 solution. Chemical shifts and coupling constants were recorded in units of parts per million and hertz, respectively. The peak of a solvent was used as internal standard. Elemental analysis was performed on a CHNS EuroVector EA-3000 EA analyzer. Melting points were determined by capillary method on a SRS OptiMelt MPA100 apparatus and are uncorrected. Specific optical rotation was measured on an Autopol V Plus automatic polarimeter (Rudolph Research Analytical) in a 1 dm cuvette at 25 °C at a wavelength of 589 nm for all substances, while additional wavelengths 365, 405, 436, 546 and 633 nm were used for (4*R*,5*R*)-**8a´**. The enantiomeric purity of the products was determined by HPLC analysis on Shimadzu Prominence LC-20AD (Spd-20a uv vis detector, Cto-20a column over, Dgu-20a degassing unit) equipped with a chiral stationary phase column (ChiralPAK AD-3) with hexane/2-propanol as eluent (flow rate 1.2 mL/min, column temperature 30 °C). Monitoring of the reaction was done by TLC on Merck plates (display with I_2 vapor and UV light). Aziridine **9** was obtained by the described method [1].

2. Synthetic procedures

Tricyclo[4.3.1.1^{3,7}]undec-4-en (2)

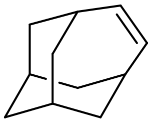

Conc. H_3PO_4 (10 mL) was added to tricyclo[4.3.1.1^{3,8}]-undecane-4-ol (**1**, 5 g, 0.03 mol). The resulting mixture was heated to 180 °C in a sublimation apparatus. The sublimed crystals were dissolved in CH_2Cl_2, the organic solution was washed with water (2 × 50 mL), dried over Na_2SO_4, and concentrated under reduced pressure. Yield of **2** is 2.5 g (56%), colorless crystals, mp 237–238 °C [lit. [2] mp 237.5–238.5 °C].

5-Chlorotricyclo[4.3.1.1^{3,8}]undecane-4-one oxime (3)

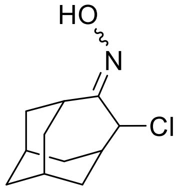

Conc. HCl (23.4 mL) was added dropwise to a solution of tricyclo[4.3.1.1^{3,8}]undec-4-ene (**2**, 5.9 g, 0.04 mol) in *tert*-butyl nitrite (14 mL) at −10 °C. The solution was stirred for 3 h at −10 °C. The precipitate was filtered, washed with petroleum ether, and recrystallized from EtOH. Yield of **3** is 4.7 g (55%), colorless crystals, mp 158–160 °C.

IR (ATR): 2907, 2859, 1665, 1464, 1354, 1062, 707 cm^{-1}.

^{1}H NMR ($CDCl_3$, 400 MHz), δ, ppm: 8.57 (br s, 1H, OH), 5.11 (d, 1H, J = 4.8 Hz, CH), 2.89-2.93 (m, 1H, CH), 2.45-2.50 (m, 1H, CH), 2.33-2.41 (m, 1H, CH), 2.03-2.12 (m, 1H, CH), 1.91-1.99 (m, 5H, 5CH), 1.50-1.70 (m, 5H, 5CH).

^{13}C NMR ($CDCl_3$, 100 MHz), δ, ppm: 165.2 (C=N), 55.9 (CH), 38.3 (CH_2), 38.2 (CH), 35.8 (CH), 35.8 (CH_2), 34.1 (CH_2), 32.0(CH_2), 31.9 (CH_2), 26.8 (CH), 26.7 (CH).

Anal. Calcd for $C_{11}H_{16}ClNO$: C, 61.82; H, 7.55; N, 6.55. Found: C, 61.89; H, 7.51; N, 6.59.

5-Azidotricyclo[4.3.1.1^{3,8}]undecane-4-one oxime (4)

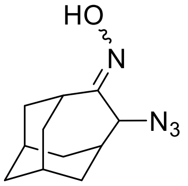

A solution of NaN_3 (0.24 g, 3.75 mmol) in water (3.8 mL) was added to a solution of 5-chlorotricyclo[4.3.1.1^{3,8}]undecane-4-one oxime (**3**, 0.8 g, 3.75 mmol) in acetone (3.8 mL). The mixture was stirred for 2 h at room temperature, then acetone was evaporated in vacuo. The residue was extracted with $CHCl_3$ (3 × 2 mL). The total organic extracts were dried over Na_2SO_4, and concentrated under reduced pressure. The product was recrystallized from MeOH. Yield of **4** is 0.44 g (53%), colorless crystals, mp 121–122 °C.

IR (ATR): 3200, 2899, 2853, 2087, 1443, 1236, 939 cm^{-1}.

^{1}H NMR ($CDCl_3$, 400 MHz), δ, ppm: 9.06 (br s, 1H, OH), 4.28 (d, J = 5.2 Hz, 1H, CH), 3.74-3.79 (m, 1H, CH), 2.02-2.26 (m, 1H, CH), 1.78-1.99 (m, 6H, 6CH), 1.49-1.70 (m, 6H, 6CH).

^{13}C NMR ($CDCl_3$, 100 MHz), δ, ppm: 164.2 (C=N), 68.1 (CH), 35.7 (CH_2), 34.8 (CH_2), 34.1 (CH_2), 33.7 (CH), 32.1 (CH_2), 31.2 (CH_2), 29.3 (CH), 26.8 (2CH).

Anal. Calcd for $C_{11}H_{16}N_4O$: C, 59.98; H, 7.32; N, 25.44. Found: C, 59.94; H, 7.36; N, 25.41.

5-(Benzylamino)tricyclo[4.3.1.1^{3,8}]undecan-4-one oxime (5)

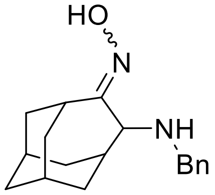

Benzylamine (1.07 g, 1.09 mL, 10 mmol) was added dropwise to a solution of 5-chlorotricyclo[4.3.1.1^{3,8}]undecan-4-one oxime (**3**, 1 g, 5 mmol) in CH_2Cl_2 (5 mL). After stirring for 2 h at room temperature, the mixture was washed with water (2 × 10 mL). The organic extracts were dried over Na_2SO_4, and concentrated under reduced pressure. Yield of **5** is 0.68 g (51%), light purple crystals, mp 115-117 °C.

IR (ATR): 3059, 3026, 2897, 2840, 1603, 1452, 1356, 932 cm^{-1}.

^1H NMR ($CDCl_3$, 400 MHz), δ, ppm: 7.27-7.35 (m, 5H, CH and OH), 7.20-7.24 (m, 1H, CH), 3.89 (d, *J* = 13.5 Hz, 1H, CH), 3.76 (d, *J* = 13.5 Hz, 1H, CH), 3.65 (t, *J* = 6.0 Hz, 1H, CH), 3.37 (d, *J* = 2.3 Hz, 1H, CH), 2.19-2.22 (m, 1H, CH), 1.96-2.02 (m, 1H, CH), 1.88-1.93 (m, 3H, 3CH), 1.67-1.85 (m, 4H, 4CH), 1.51-1.63 (m, 4H, 4CH).

^{13}C NMR ($CDCl_3$, 100 MHz), δ, ppm: 165.9 (C=N), 140.3 (C), 128.4 (2CH), 128.1 (2CH), 126.9 (CH), 65.2 (CH), 51.4 (CH_2), 37.4 (CH_2), 36.2 (CH_2), 34.4 (CH_2), 33.1 (CH), 32.9 (CH_2), 30.0 (CH_2), 29.6 (CH), 27.0 (CH), 26.9 (CH).

Anal. Calcd for $C_{18}H_{24}N_2O$: C, 76.02; H, 8.51; N, 9.85. Found: C, 76.10; H, 8.55; N, 9.83.

Tricyclo[4.3.1.1^{3,8}]undecane-4-amine hydrochloride (6)

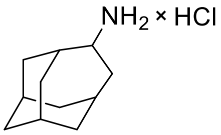

$NaBH_4$ (0.5 g, 0.013 mol) was added portionwise to a mixture of 5-azidotricyclo[4.3.1.1^{3,8}]undecan-4-one oxime (**4**, 0.8 g, 4 mmol) and $NiCl_2$ (0.34 g, 2.6 mmol) in MeOH (5 mL) at 10 °C. The reaction mixture was stirred for 6 h at room temperature, then poured into water (25 mL). A 5% aq NaOH solution was added to adjust pH 14, and the mixture was extracted with CH_2Cl_2 (5 × 5 mL). The total organic extracts were dried over Na_2SO_4, and the solvent was evaporated under reduced pressure. The residue was purified by column chromatography ($CHCl_3$–EtOH, 50:1). The resulting oil was dissolved in Et_2O and HCl gas was passed through the solution. Yield of **6** is 0.39 g (49%), yellow crystals, mp 283–285 °C [1,3].

IR (ATR): 3404, 2901, 2848, 1560, 1447 cm^{-1}.

^1H NMR data for the **free amine** ($CDCl_3$, 400 MHz), δ, ppm: 4.97 (br s, 2H, NH_2), 3.39 (t, *J* = 8 Hz, 1H, CH), 2.41-2.49 (m, 1H, CH), 2.06-2.13 (m, 2H, 2CH), 1.83-1.99 (m, 6H, 6CH), 1.74-1.79 (m, 1H, CH), 1.45-1.60 (m, 6H, 6CH).

^{13}C NMR data for the **free amine** ($CDCl_3$, 100 MHz), δ, ppm: 57.0 (CH), 41.4 (CH_2), 40.6 (CH_2), 38.3 (CH), 38.1 (CH_2), 36.3 (CH_2), 34.6 (CH_2), 29.6 (CH), 29.4 (CH_2), 27.0 (CH), 26.7 (CH).

5-Azidotricyclo[4.3.1.1$^{3,8]}$undecan-4-amine hydrochloride (7)

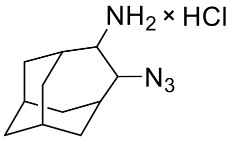

MoO_3×H_2O (2.48 g, 17.0 mmol) was added to a solution of 5-azidotricyclo[4.3.1.1^{3,8}]undecane-4-one oxime (**4**, 1.1 g, 5.3 mmol) in MeOH (10 mL). Then, $NaBH_4$ (1.28 g, 34.0 mmol) was added portionwise to the resulting suspension at 0 °C. The reaction mixture was stirred for 2 h at room temperature and poured into water (30 mL). A 5% aq NaOH solution was added to adjust pH 14, and the mixture was extracted with CH_2Cl_2 (5 × 5 mL). The total organic extracts were dried over

Na_2SO_4, and the solvent was evaporated under reduced pressure. The resulting oil was dissolved in Et_2O and HCl gas was passed through the solution. Yield of **7** is 0.7 g (69%), colorless crystals, mp 190–193 °C.

IR (ATR): 3360, 2903, 2853, 2106, 1447, 1271, 1100, 943 cm^{-1}.

^{1}H NMR (DMSO-d_6, 400 MHz), δ, ppm: 8.12 (br s, 3H, NH_3^+), 4.32-4.36 (m, 1H, CH), 3.56 (t, 1H, J = 6.4 Hz, CH), 2.29-2.34 (m, 1H, CH), 2.05-2.09 (m, 1H, CH), 1.70-1.80 (m, 6H, 6CH), 1.45-1.58 (m, 6H, 6CH).

^{13}C NMR (DMSO-d_6, 100 MHz), δ, ppm: 65.2 (CH), 56.3 (CH), 37.3 (CH_2), 36.1 (CH_2), 34.8 (CH), 34.2 (CH), 32.2 (CH_2), 31.5 (CH_2), 29.0 (CH_2), 26.4 (CH), 26.3 (CH).

Anal. Calcd for $C_{11}H_{19}ClN_4$: C, 54.43; H, 7.89; N, 23.08. Found: C, 54.47; H, 7.86; N, 23.10.

trans/*cis*-4,5-Diaminohomoadamantane dihydrochloride (8a,b)

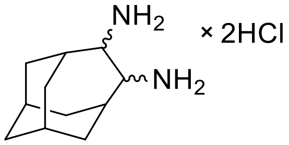

A solution of 5-azidotricyclo[4.3.1.1^{3,8}]undecane-4-one oxime (**4**, 0.78 g, 3.55 mmol) in Et_2O (8 mL) was added to the suspension of $LiAlH_4$ (0.54 g, 14.2 mmol) in Et_2O (11 mL) at 0 °C under an inert atmosphere. The reaction mixture was stirred at room temperature for 2 h, cooled to 5 °C, and then quenched by the dropwise successive addition of water (0.54 mL), a 15% aqueous NaOH solution (0.54 mL), and water (1.62 mL). The mixture was stirred at room temperature for an additional 1 h. The precipitate was filtered off and washed with Et_2O (3 × 5 mL). The filtrate was evaporated under reduced pressure. The residue was dissolved in MeOH and HCl gas was passed through. The precipitate was filtered off. Yield of **8a,b** is 0.52 g (58%), *dr* = 5:1, colorless crystals, mp 338–340 °C [4].

IR (ATR): 3020, 2900, 2850, 2548, 1598, 1571, 1500, 1452, 1040,

1012 cm^{-1}.

^{1}H NMR (DMSO-d_6, 400 MHz), δ, ppm: 8.77 (s, 6H, $2NH_2$×2HCl), 8.67 (s, 6H, $2NH_2$×2HCl), 3.86 (s, 2H, 2CH), 3.38 (s, 2H, 2CH), 2.21-2.25 (m, 2H, 2CH), 2.10-2.14 (m, 2H, 2CH), 1.52-1.91 (m, 20H, 20CH), 1.47 (s, 4H, 4CH).

^{13}C NMR (DMSO-d_6, 100 MHz), δ, ppm: 60.1 (2CH *trans*), 55.6 (2CH *cis*), 36.5 (CH_2 *trans*), 35.6 (CH_2 *trans*), 34.8 (CH_2 *cis*), 34.7 (CH *cis*), 34.4 (2CH *trans*), 29.9 (CH_2 *cis*), 29.0 ($3CH_2$ *trans*), 26.1 (CH *cis*), 25.8 (CH *cis*), 25.7 (2CH *trans*).

Anal. Calcd for $C_{11}H_{22}Cl_2N_2$: C, 52.18; H, 8.76; N, 11.06. Found: C, 52.21; H, 8.74; N, 11.09.

tert-Butyl 5-azatetracyclo[5.3.1.1^{3,9}.0^{4,6}]dodecane-5-carboxylate (10)

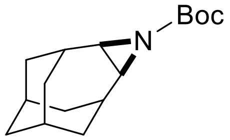

To a solution of 5-azatetracyclo[5.3.1.1^{3,9}.0^{4,6}]dodecane (**9**, 0.89 g, 5.4 mmol) in CH_2Cl_2 (15 mL) at 0 °C were added Et_3N (1.18 g, 1.2 mL, 11.6 mmol) and Boc_2O (1.18 g, 5.4 mmol). The reaction mixture was stirred at room temperature for 1 h and then washed with water (2 × 20 mL) and saturated NaCl solution (2 × 20 mL). The organic layer was dried over Na_2SO_4, and the solvent was evaporated under reduced pressure. The residue was purified by column chromatography (petroleum ether/EtOAc 10:1). Yield of **10** is 1.2 g (80%), colorless crystals, mp 40–42 °C.

IR (ATR): 2897, 2846, 1680, 1529, 1448, 1363, 1253, 1163, 1053, 1020 cm^{-1}.

^{1}H NMR ($CDCl_3$, 400 MHz), δ, ppm: 2.54-2.59 (m, 2H, 2CH), 2.39-2.43 (m, 2H, 2CH), 1.68-1.87 (m, 6H, 6CH), 1.51-1.62 (m, 6H, 6CH), 1.41 (s, 9H, $3CH_3$).

^{13}C NMR ($CDCl_3$, 100 MHz), δ, ppm: 163.2 (C=O), 80.2 (C-O), 44.9 (2CH), 36.2 (CH_2), 34.3 ($2CH_2$), 30.6 ($2CH_2$), 30.0 (2CH), 28.1 ($3CH_3$), 27.8 (CH), 25.7 (CH).

Anal. Calcd for $C_{16}H_{25}NO_2$: C, 72.97; H, 9.57; N, 5.32. Found: C, 72.92; H, 9.61; N, 5.36.

cis-5-*p*-Tosyl-5-azatetracyclo[5.3.1.1^{3,9}.0^{4,6}]dodecane (11)

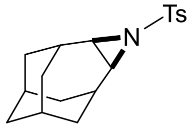

To a solution of 5-azatetracyclo[5.3.1.1^{3,9}.0^{4,6}]dodecane (**9**, 0.2 g, 1.2 mmol) in CH_2Cl_2 (10 mL) at 0 °C were added Et_3N (0.14 g, 0.19 mL, 1.4 mmol) and *p*-TsCl (0.26 g, 1.4 mmol). The reaction mixture was stirred at room temperature for 2 h, washed with 5% HCl solution (2 × 15 mL) and 5% aqueous $NaHCO_3$ solution (2 × 15 mL). The organic layer was dried over Na_2SO_4, and the solvent was evaporated under reduced pressure. The residue was purified by column chromatography (petroleum ether/EtOAc 10:1). Yield of **11** is 0.34 g (87%), colorless crystals, mp 80–82 °C [5].

IR (ATR): 2910, 2845, 1597, 1448, 1323, 1155, 1083, 867, 796, 667, 572, 557 cm^{-1}.

^{1}H NMR ($CDCl_3$, 400 MHz), δ, ppm: 7.80 (d, J = 8.0 Hz, 2H, 2CHAr), 7.29 (d, J = 8.0 Hz, 2H, 2CHAr), 2.95-2.99 (m, 2H, 2CH), 2.42 (s, 3H, CH_3), 2.33 (br s, 2H, 2CH), 1.87 (br s, 1H, CH), 1.46-1.76 (m, 9H, 9CH), 1.41 (s, 2H, 2CH).

^{13}C NMR ($CDCl_3$, 100 MHz), δ, ppm: 143.9 (C), 136.1 (C), 129.3 (2CH), 127.6 (2CH), 47.0 (2CH), 36.1 (CH_2), 33.9 ($2CH_2$), 30.6 ($2CH_2$), 29.5 (2CH), 27.6 (CH), 25.6 (CH), 21.7 (CH_3).

Anal. Calcd for $C_{18}H_{23}NO_2S$: C, 68.11; H, 7.30; N, 4.41; S, 10.10. Found: C, 68.15; H, 7.34; N, 4.38; S, 10.17.

cis-5-((Trifluoromethyl)sulfonyl)-5-azatetracyclo[5.3.1.1^{3,9}.0^{4,6}] dodecane (12)

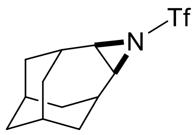

To a solution of 5-azatetracyclo[5.3.1.1^{3,9}.0^{4,6}]dodecane (**9**, 7.37 g, 44.9 mmol) in CH_2Cl_2 (70 mL) at 0 °C were added Et_3N (6.84 g, 9.4 mL, 67.8 mmol) and Tf_2O (14.25 g, 8.5 mL, 50.5 mmol). The reaction mixture was stirred at room temperature for 2 h, washed with 5% HCl solution (2 × 50 mL), 5% aqueous $NaHCO_3$ solution (2 × 50 mL), and water (2 × 50 mL). The organic layer was dried over Na_2SO_4, and the solvent was evaporated under reduced pressure. The residue was purified by column chromatography (petroleum ether). Yield of **12** is 10.1 g (76%), light yellow oil.

IR (ATR): 2902, 2852, 1444, 1361, 1179, 1126, 948, 931, 615 cm^{-1}.

^{1}H NMR ($CDCl_3$, 400 MHz), δ, ppm: 3.29-3.34 (m, 2H, 2CH), 2.48-2.52 (m, 2H, 2CH), 1.56-1.94 (m, 10H, 10CH), 1.47 (s, 2H, 2CH).

^{13}C NMR ($CDCl_3$, 100 MHz), δ, ppm: 119.1 (CF_3, J = 319.4 Hz), 49.4 (2CH), 35.8 (CH_2), 33.3 (2CH_2), 30.4 (2CH_2), 29.4 (2CH), 27.3 (CH), 25.3 (CH).

Anal. Calcd for $C_{12}H_{16}F_3NO_2S$: C, 48.81; H, 5.46; N, 4.74; S, 10.86. Found: C, 48.87; H, 5.41; N, 4.78; S, 10.80.

trans/*cis*-*N*-(5-Azidotricyclo[4.3.1.1^{3,8}]undecan-4-yl)-4-methylbenzene sulfonamide (13a,b)

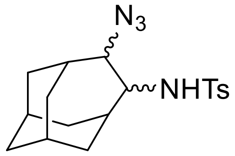

To a solution of 5-*p*-tosyl-5-azatetracyclo[5.3.1.1^{3,9}.0^{4,6}]dodecane (**11**,0.34 g, 1.0 mmol) in DMF (15 mL) were added NaN_3 (1 g, 15.4 mmol) and NH_4Cl (1.36 g, 25.4 mmol). The reaction mixture was stirred at 120–125 °C for 24 h under an inert atmosphere. After cooling, the mixture was poured into water (50 mL) and extracted with EtOAc (5 × 5 mL). The combined organic extracts were dried over Na_2SO_4, and the solvent was evaporated under reduced pressure. The residue was purified by column chromatography (CCl_4). Yield of **13a,b** is 0.15 (39%), dr = 1:2, colorless crystals, mp 160–164 °C.

IR (ATR): 3251, 2906, 2848, 2092, 1448, 1323, 1259, 1149, 1083, 804, 794, 667, 545 cm^{-1}.

^{1}H NMR ($CDCl_3$, 400 MHz), δ, ppm: 7.76-7.82 (m, 4H, 2CH $_{trans}$ + 2CH $_{cis}$), 7.27-7.33 (m, 4 H, 2CH $_{trans}$ + 2CH $_{cis}$), 5.19 (d, J = 6.8 Hz, 1H, NH $_{cis}$), 5.04 (d, J = 6.0 Hz, 1H, NH $_{trans}$), 3.75-3.77 (m, 1H, CH $_{trans}$), 3.50-3.53 (m, 1H, CH $_{trans}$), 3.29-3.31 (m, 1H, CH $_{cis}$), 3.19-3.22 (m, 1H, CH $_{cis}$), 2.67 (s, 3H, CH_3 $_{cis}$), 2.41 (s, 3H, CH_3 $_{trans}$), 1.52-2.27 (m, 28H, 14CH $_{trans}$ + 14CH $_{cis}$).

^{13}C NMR ($CDCl_3$, 100 MHz), δ, ppm: 143.5 (C $_{trans}$ + C $_{cis}$), 137.6 (C $_{cis}$), 136.9 (C $_{trans}$), 129.6 (2CH $_{cis}$), 129.5 (2CH $_{trans}$), 127.6 (2CH $_{trans}$), 127.2 (2CH $_{cis}$), 74.8 (CH $_{cis}$), 72.6 (CH $_{trans}$), 69.5 (CH $_{trans}$), 65.5 (CH $_{cis}$), 39.9 (CH $_{trans}$), 39.0 (CH_2 $_{trans}$), 38.3 (CH_2 $_{trans}$), 38.2 (CH_2 $_{cis}$), 38.0 (CH_2 $_{cis}$), 37.2 (CH $_{trans}$), 37.0 (CH $_{cis}$), 36.1 (CH_2 $_{trans}$), 36.0 (CH_2 $_{cis}$), 35.8 (CH $_{cis}$), 29.7 (CH_2 $_{trans}$), 29.4 (CH_2 $_{cis}$), 29.1 (CH_2 $_{cis}$), 29.0 (CH_2 $_{trans}$), 26.2 (CH $_{cis}$), 26.0 (2CH $_{trans}$ + 2CH $_{cis}$), 25.9 (2CH $_{trans}$).

***trans-N*-(5-Azidotricyclo[4.3.1.1^{3,8}]undecan-4-yl)-1,1,1-trifluoromethane sulfonamide (14)**

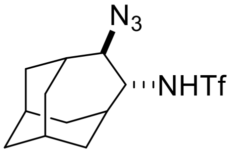

To a solution of 5-((trifluoromethyl)sulfonyl)-5-azatetra- cyclo[5.3.1.1^{3,9}.0^{4,6}]dodecane (**12**, 9.6 g, 32.6 mmol) in DMF (130 mL) were added NaN_3 (10.6 g, 0.16 mol) and NH_4Cl (8.7 g, 0.16 mol). The reaction mixture was stirred at 110–120 °C for 12 h under an inert atmosphere. After cooling, the mixture was poured into water (700 mL) and extracted with EtOAc (5 × 40 mL). The combined organic extracts were dried over Na_2SO_4, and the solvent was evaporated under reduced pressure. The residue was purified by column chromatography (CCl_4). Yield of **14** is 9.6 (87%), colorless crystals, mp 102–103 °C.

IR (ATR): 3275, 2906, 2860, 2102, 1448, 1379, 1226, 1192, 1147, 1035, 609 cm^{-1}.

^{1}H NMR (DMSO-d_6, 400 MHz), δ, ppm: (s, 1H, NH), 3.67-3.69 (m, 1H, CH), 3.34 (br s, 1H, CH), 2.12-2.15 (m, 1H, CH), 1.93-1.95 (m, 1H, CH), 1.43-1.84 (m, 12H, 12CH).

^{13}C NMR (DMSO-d_6, 100 MHz), δ, ppm: 119.9 (CF_3, J = 321.3 Hz), 72.9 (CH), 67.5 (CH), 39.1 (CH), 38.1 (CH_2), 37.9 (CH_2), 35.9 (CH), 35.8 (CH_2), 29.5 (CH_2), 28.8 (CH_2), 26.0 (CH), 25.9 (CH).

Anal. Calcd for $C_{12}H_{17}F_3N_4O_2S$: C, 42.60; H, 5.06; N, 16.56; S, 9.48. Found: C, 42.66; H, 5.10; N, 16.51; S, 9.52.

***trans-N*-(5-Aminotricyclo[4.3.1.1^{3,8}]undecan-4-yl)-1,1,1-trifluoromethanesulfonamide (15)**

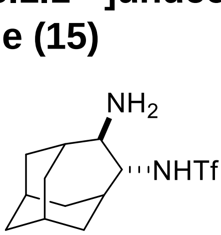

A solution of *trans-N*-(5-azidotricyclo[4.3.1.1^{3,8}]undecane-4-yl)-1,1,1-tri-fluoromethanesulfonamide (**14**, 0.25 g, 0.74 mmol) in THF (5 mL) was added dropwise to a suspension of $LiAlH_4$ (0.11 g, 3.0 mmol) in THF (30 mL) at 5 °C under an inert atmosphere. The reaction mixture was stirred at room temperature for 4 h, cooled to 5 °C, and then quenched by the dropwise successive addition of water (0.11 mL), 15% aqueous NaOH solution (0.11 mL), and water (0.33 mL). After stirring at room temperature for an additional 1 h, the precipitate was filtered off and washed with Et_2O

(3 × 5 mL). The filtrate was evaporated under reduced pressure. Yield of **15** is 0.13 g (57%), colorless oil.

IR (ATR): 2900, 2849, 1647, 1446, 1379, 1259, 1186, 798, 607 cm^{-1}.

^1H NMR (DMSO-d_6, 400 MHz), δ, ppm: (s, 2H, NH_2), 4.26-4.31 (m, 1H, NH), 3.12 (d, 1H, J = 7.2 Hz, CH), 2.88 (d, 1H, J = 7.6 Hz, CH), 1.95-1.98 (m, 1H, CH), 1.73-1.85 (m, 6H, 6CH), 1.45-1.60 (m, 5H, 5CH).

^{13}C NMR (DMSO-d_6, 100 MHz), δ, ppm: 123.0 (CF_3, J = 330.8 Hz), 66.8 (CH), 65.3 (CH), 39.5 (CH), 39.0 (CH_2), 38.8 (CH_2), 33.5 (CH), 36.6 (CH_2), 29.4 (CH_2), 29.1 (CH_2), 26.3 (CH), 26.2 (CH).

Anal. Calcd for $C_{12}H_{19}F_3N_2O_2S$: C, 46.14; H, 6.13; N, 8.97; S, 10.26. Found: C, 46.18; H, 6.09; N, 8.93; S, 10.22.

trans-4,5-Diaminohomoadamantane dihydrochloride (8a)

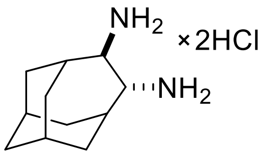

A Red-Al solution (60% in toluene, 34.9 g, 33.7 mL, 0.1 mol) was added dropwise to a solution of *N*-(5-azidotricyclo[4.3.1.1^{3,8}]undecan-4-yl)-1,1,1-trifluoromethane sulfonamide (**14**, 5 g, 14.8 mmol) in toluene (50 mL) under an inert atmosphere. The reaction mixture was heated under reflux for 48 h, cooled to room temperature, and then quenched by the dropwise addition of a 4% aqueous NaOH solution (50 mL). After stirring for 6 h, the precipitate was filtered off and washed with toluene (3 × 10 mL). The organic layer was separated, dried over Na_2SO_4, and concentrated under reduced pressure. The residue was dissolved in a minimal amount of MeOH, acidified to pH 1 with concentrated HCl, and treated with MTBE. The resulting precipitate was collected by filtration. The reprecipitation was repeated in the MeOH/MTBE. Yield of **8a** is 1.65 g (44%), colorless crystals, mp >290 °C (with decomposition) (lit. [4] mp >300 °C (with decomposition)).

IR (ATR): 3020, 2900, 2850, 2546, 1597, 1573, 1500, 1454, 1041, 1014, 551 cm^{-1}.

^1H NMR (DMSO-d_6, 400 MHz), δ, ppm: (s, 6H, $2NH_2$×2HCl), 3.34 (s, 2H, 2CH), 2.16 (br s, 2H, 2CH), 1.97-2.00 (m, 2H, 2CH), 1.79 (br s, 4H, 4CH), 1.59-1.63 (m, 4H, 4CH), 1.47 (s, 2H, 2CH).

^{13}C NMR (DMSO-d_6, 100 MHz), δ, ppm: 60.1 (2CH), 36.5 (CH_2), 35.6 (CH_2), 34.4 (2CH), 29.0 ($3CH_2$), 25.7 (2CH).

trans-4,5-Diaminohomoadamantane (8a′)

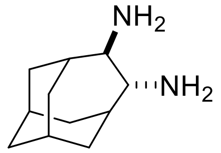

To a stirred solution of *trans*-4,5-diaminohomoadamantane dihydrochloride (**8a**, 0.86 g, 3.43 mmol) in water (10 mL) and CH_2Cl_2 (5 mL) was added NaOH (0.26 g, 6.5 mmol). The mixture was stirred for 15 min, and the organic layer was separated. The aqueous layer was extracted with CH_2Cl_2 (4 × 5 mL). The combined organic extracts were dried over K_2CO_3, and the solvent was evaporated under reduced pressure. Yield of **8a′** is 0.56 g (90%), colorless oil.

IR (ATR): 3336, 3267, 2897, 2846, 1589, 1450, 1346, 902, 860, 725 cm^{-1}.

(4*R*,5*R*)-4,5-Diaminohomoadamantane ((4*R*,5*R*)-8a′)

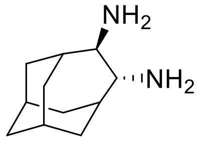

A mixture of *trans*-4,5-diaminohomoadamantane **8a′** (0.8 g, 5.3 mmol) and dibenzoyl-L-tartaric acid (4.01 g, 10.7 mmol) in water (12 mL) and EtOH (6 mL) was heated under reflux for 15 min. The solvent was evaporated under reduced pressure. The residue was dissolved in a minimal amount of hot MeOH, $CHCl_3$ (3 mL) was added, the mixture was left for 24 h at 5 °C, the precipitate was filtered off. Reprecipitation was repeated twice until a constant optical rotation was achieved ($[\alpha]_D^{25}$= +14.1° (*c* 0.5, EtOH–H_2O, 1:1)). Yield of salt is 0.72 g, colorless crystals.

To a stirred solution of the salt (0.72 g) in water (30 mL) and CH_2Cl_2 (10 mL) was added NaOH (0.13 g, 3.25 mmol). After stirring for 15 min, the organic layer was separated. The aqueous layer was extracted with CH_2Cl_2 (4 × 5 mL). The combined organic extracts were dried over K_2CO_3, and the solvent was evaporated under reduced pressure. Yield of (4*R*,5*R*)-**8a′** is 0.11 g (15%), colorless oil, $[\alpha]_D^{25}$= +13.1° (*c* 0.2, CH_2Cl_2).

trans-Decahydro-4,8:6,10-dimethanocyclonona[*d*]imidazole-2(1*H*)-thione (16)

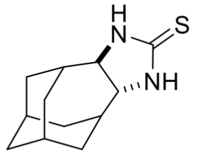

Carbon disulfide (42 mg, 0.03 mL, 0.61 mmol) was added to a solution of *trans*-4,5-diaminohomoadamantane (**8a′**, 0.1 g, 0.56 mmol) in EtOH (8 mL) and water (2 mL). The reaction mixture was heated under reflux for 3 h. Then, three drops of concentrated HCl were added, and the mixture was heated under reflux for an additional 5 h. The solvent was evaporated under reduced pressure, and the residue

was recrystallized from aqueous EtOH. Yield of **16** is 91 mg (74%), colorless crystals, mp 270–278 °C (with decomposition).

IR (ATR): 3020, 2900, 2850, 2546, 1597, 1573, 1500, 1454, 1041, 1014, 551 cm^{-1}.

^{1}H NMR ($CDCl_3$, 400 MHz), δ, ppm: 7.94 (s, 2 H, 2NH), 4.01 (s, 2H, 2CH), 1.89 (s, 2H, 2CH), 1.72-1.80 (m, 4H, 4CH), 1.49-1.57 (m, 6H, 6CH), 1.33-1.36 (m, 2H, 2CH).

^{13}C NMR ($CDCl_3$, 100 MHz), δ, ppm: 180.2 (C=S), 64.9 (2CH), 35.9 (CH_2), 34.6 (2CH), 34.3 (2CH_2), 30.8 (2CH_2), 26.8 (CH), 26.1 (CH).

Anal. Calcd for $C_{12}H_{18}N_2S$: C, 64.82; H, 8.16; N, 12.60; S, 14.42. Found: C, 64.87; H, 8.11; N, 12.64; S, 14.38.

2,3-Diphenyl-4*a*,6,7,8,9,10,11,11*a*-octahydro-5*H*-5,9:7,11-dimethanocyclonona[*b*]pyrazine (17)

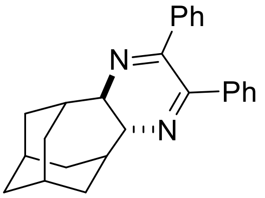

A mixture of *trans*-4,5-diaminohomoadamantane (**8a´**, 0.12 g, 0.7 mmol), 1,2-diphenylethane-1,2-dione (0.12 g, 0.6 mmol) and *p*-TsOH (13 mg, 0.07 mmol) in EtOH (5 mL) was heated under reflux for 3 h. The mixture was cooled to room temperature, and the precipitate that formed was filtered off. Yield of **17** is 0.13 g (56%), light yellow crystals, mp 165–167 °C.

IR (ATR): 3059, 3028, 2900, 2850, 1539, 1442, 1249, 1022, 748, 694, 584 cm^{-1}.

^{1}H NMR ($CDCl_3$, 400 MHz), δ, ppm: 7.39-7.42 (m, 4H, 4CH), 7.20-7.28 (m, 6H, 6CH), 2.86 (s, 2H, 2CH), 2.74 (s, 2H, 2CH), 1.89-2.00 (m, 8H, 8CH), 1.63 (s, 4H, 4CH).

^{13}C NMR ($CDCl_3$, 100 MHz), δ, ppm: 159.5 (2C=N), 137.6 (2C), 129.4 (2CH), 128.2 (4CH), 128.0 (4CH), 68.8 (2CH), 40.4 (2CH_2), 37.6 (CH_2), 37.1 (2CH), 29.9 (2CH_2), 27.3 (2CH).

Anal. Calcd for $C_{25}H_{26}N_2$: C, 84.70; H, 7.39; N, 7.90. Found: C, 84.79; H, 7.35; N, 7.86.

(4*R*,5*R*)-2,3-Diphenyl-4*a*,6,7,8,9,10,11,11*a*-octahydro-5*H*-5,9:7,11-dimethanocyclonone[*b*]pyrazine ((4*R*,5*R*)-17)

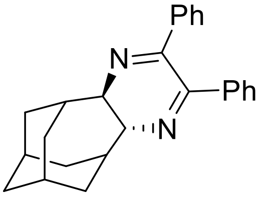

The compound (4*R*,5*R*)-**17** was obtained by a similar procedure from (4*R*,5*R*)-4,5-diaminohomoadamantane (0.03 g, 0.17 mmol), the product was purified by column

chromatography ($CHCl_3$). Yield of (4*R*,5*R*)-**17** is 0.04 g (68%), 95% *ee*, light yellow crystals, mp 180-182°C, $[\alpha]_D^{25}$= +54.4° (*c* 0.2, $CHCl_3$).

HPLC analysis (ChiralPAK AD-3 column; hexane/2-propanol 93:7; flow rate 1.2 mL/min; wavelength 210 nm): t_R = 9.9 min (4*R*,5*R*)-**17**, 11.3 min (4*S*,5*S*)-**17**

(4*R*,5*R*)-*N*,*N*′-(Tricyclo[4.3.1.1^{3,8}]undecane-4,5-diyl)bis(1-phenylmethanimine) (18)

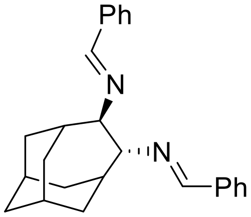

A mixture of (4*R*,5*R*)-4,5-diaminohomoadamantane (0.05 g, 0.33 mmol), *p*-TsOH (6.4 mg, 0.03 mmol) and PhCHO (0.07 g, 0.07 mL, 0.66 mmol) in MeOH (10 mL) was heated under reflux for 6 h. The mixture was cooled to room temperature, and the precipitate that formed was filtered off. Yield of **18** is 76 mg (64%), colorless crystals, mp 148–150 °C, $[\alpha]_D^{25}$= +150.9° (*c* 0.1, $CHCl_3$).

IR (ATR): 3060, 2902, 2846, 1641, 1448, 761, 696 cm^{-1}.

^{1}H NMR ($CDCl_3$, 400 MHz), δ, ppm: (s, 2H, 2CH=N), 7.60-7.64 (m, 4H, 4CH), 7.30-7.34 (m, 6H, 6CH), 3.35 (s, 2H, 2CH), 2.26-2.29 (m, 2H, 2CH), 1.81-2.02 (m, 12H, 12CH).

^{13}C NMR ($CDCl_3$, 100 MHz), δ, ppm: 159.6 (2CH=N), 136.7 (2C), 130.2 (2CH), 128.5 (4CH), 128.0 (4CH), 82.1 (2CH-N), 39.6 (CH_2), 38.9 ($3CH_2$), 37.3 (CH_2), 30.8 (2CH), 27.3 (2CH).

Anal. Calcd for $C_{25}H_{28}N_2$: C, 84.22; H, 7.92; N, 7.86. Found: C, 84.32; H, 7.89; N, 7.79.

(4*R*,5*R*)-*N*,*N*′-Dibenzyltricyclo[4.3.1.1^{3,8}]undecane-4,5-diamine dihydrochloride (19)

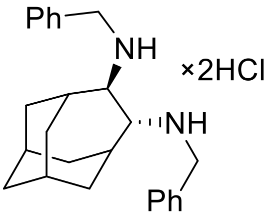

$NaBH_4$ (0.53 g, 14 mmol) was added portionwise to a stirred solution of (4*R*,5*R*)-*N*,*N*′-(tricyclo[4.3.1.1^{3,8}]undecane-4,5-yl)bis(1-phenylmethanimine) (**18**, 0.5 g, 1.4 mmol) in MeOH (50 mL). The reaction mixture was stirred at room temperature for 4 h. The solvent was evaporated, water (100 mL) was added, and the mixture was extracted with CH_2Cl_2 (3 × 30 mL). The combined organic extracts were dried over Na_2SO_4, and the solvent was evaporated. The residue was dissolved in a minimal amount of MeOH, acidified to pH 1 with conc. HCl, and then MTBE was added. The precipitate that

formed was filtered off. Yield of **19** is 0.49 g (81%), colorless crystals, mp 241–251 °C (with decomposition).

IR (ATR): 2924, 2858, 2619, 2453, 1597, 1570, 1469, 1454, 732 cm^{-1}.

Anal. Calcd for $C_{25}H_{34}Cl_2N_2$: C, 69.27; H, 7.91; N, 6.46. Found: C, 69.20; H, 7.96; N, 6.55.

(4*R*,5*R*)-*N*,*N′*-Dibenzyltricyclo[4.3.1.1^{3,8}]undecane-4,5-diamine (19′)

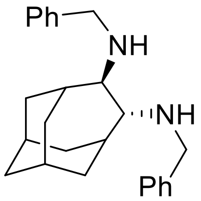

NaOH (90 mg, 2.3 mmol) was added to a stirred solution of (4*R*,5*R*)-*N*,*N′*-dibenzyltricyclo[4.3.1.1^{3,8}]undecane-4,5-diamine dihydrochloride (**19**, 0.28 g, 0.65 mmol) in water (10 mL) and CH_2Cl_2 (5 mL). The reaction mixture was stirred for 15 min. The organic layer was separated, and the aqueous layer was extracted with CH_2Cl_2 (4 × 5 mL). The combined organic extracts were dried over Na_2SO_4, and the solvent was evaporated. Yield of **19′** is 0.19 g (82%), colorless oil, $[\alpha]_D^{25}$= +110.0° (*c* 0.1, $CHCl_3$).

IR (ATR): 3300, 3028, 2904, 2846, 1600, 1448, 1352, 1140, 1028, 730, 694 cm^{-1}.

Henry reaction

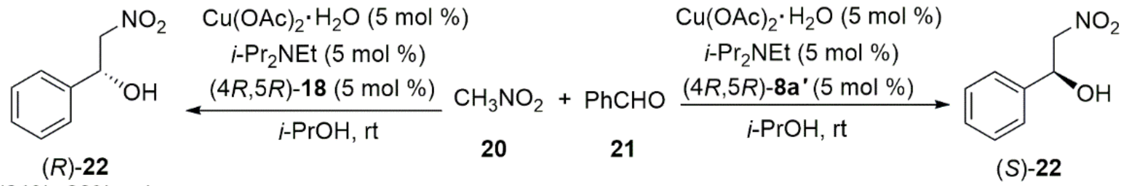

(*R*)-**22** (61%, 60% *ee*)   (*S*)-**22** (52%, 36% *ee*)

A mixture of $Cu(OAc)_2 \cdot H_2O$ (9.8 mg, 0.049 mmol) and the corresponding ligand (0.049 mmol) in iPrOH (3 mL) was sonicated for 15 min. Then, nitromethane (**20**, 0.29 g, 4.85 mmol), $(iPr)_2NEt$ (6.3 mg, 0.049 mmol), and benzaldehyde (**21**, 0.1 g, 0.98 mmol) were added. The reaction mixture was stirred for 18 h at room temperature. The solvent was evaporated under reduced pressure, and the residue was purified by column chromatography (petroleum ether/EtOAc 10:1).

Ligand (4*R*,5*R*)-**8a′** was used in an amount of 8.8 mg (5 mol %), yield of (*S*)-2-nitro-1-phenylethanol (**22**) is 85 mg (52%), 36% ee, colorless oil.

Ligand (4*R*,5*R*)-**18** was used in an amount of 17.5 mg (5 mol %), yield of (*R*)-2-nitro-1-phenylethanol (**22**) is 100 mg (61%), 60% ee, colorless oil.

HPLC analysis (ChiralPAK AD-3 column; hexane/2-propanol 98:2; flow rate 1.2 mL/min; wavelength 210 nm): t_R = 30.2 min (*R*)-**22**, 31.6 min (*S*)-**22**.

Michael reaction

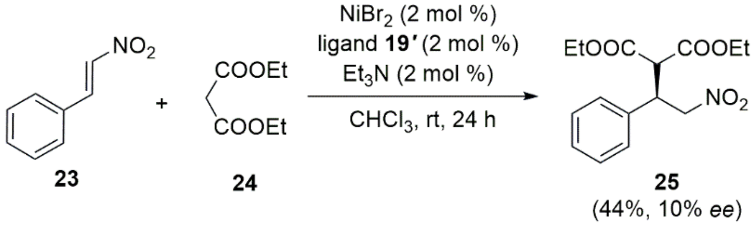

To a solution of β-nitrostyrene (**23**, 0.1 g, 0.69 mmol) and diethyl malonate (**24**, 0.1 g, 0.69 mmol) in $CHCl_3$ (4 mL) was added a mixture of ligand **19´** (5 mg, 0.014 mmol), $NiBr_2$ (3.1 mg, 0.014 mmol), and Et_3N (1.4 mg, 0.014 mmol) in $CHCl_3$ (0.5 mL). The reaction mixture was stirred at room temperature for 24 h, and then the solvent was evaporated under reduced pressure. The residue was purified by column chromatography ($CHCl_3$). Yield of (*R*)-diethyl-(2-nitro-1-phenylethyl)malonate (**25**) 88 mg (44%), 10% ee.

HPLC analysis (ChiralPAK AD-3 column; hexane/2-propanol 92:8; flow rate 1.2 mL/min; wavelength 210 nm): t_R = 14.4 min (*R*)-**25**, 35.3 min (*S*)-**25**.

3. ^{1}H NMR and ^{13}C NMR spectra for synthesized compounds

3.1. NMR spectra of 5-chlorotricyclo[4.3.1.13,8]undecane-4-one oxime (**3**)

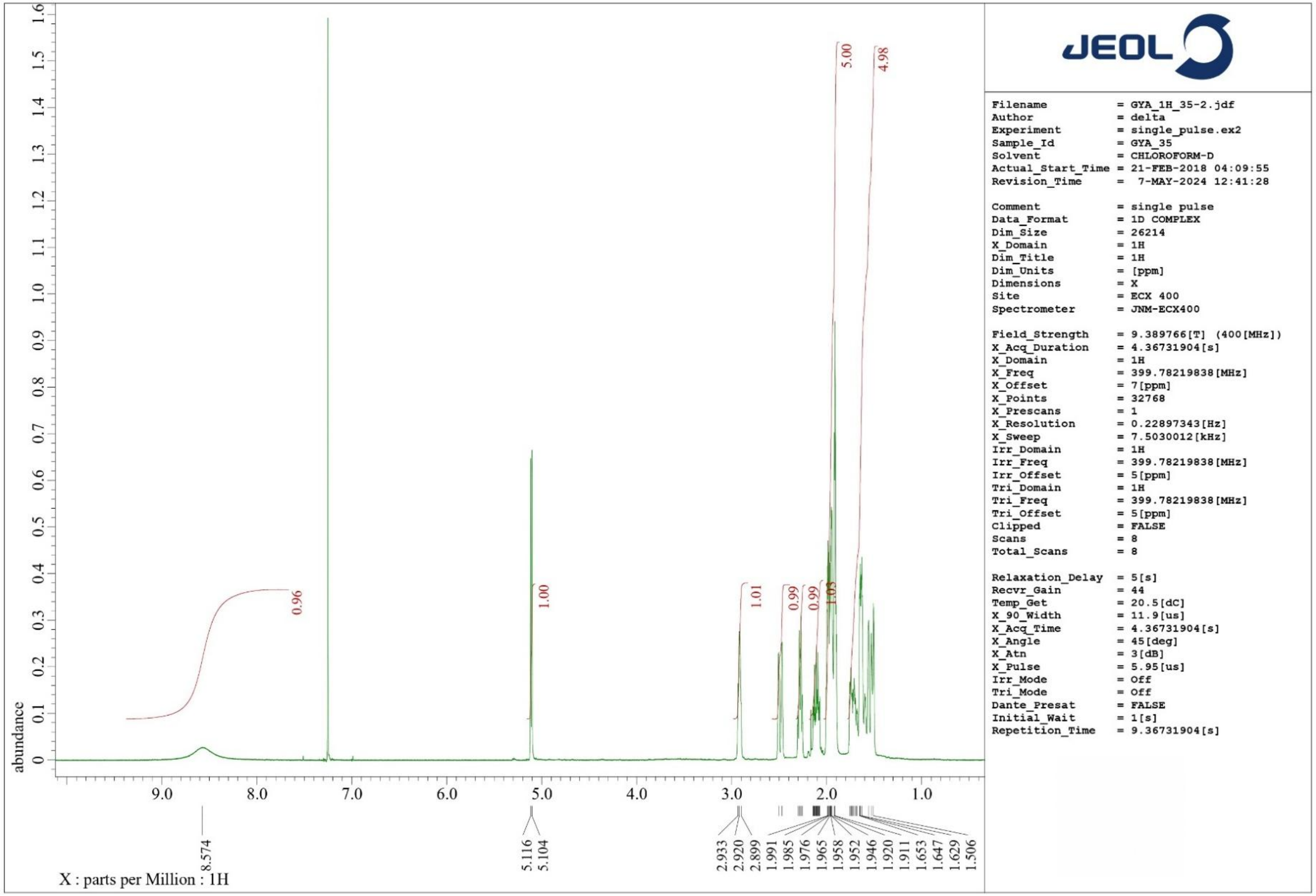

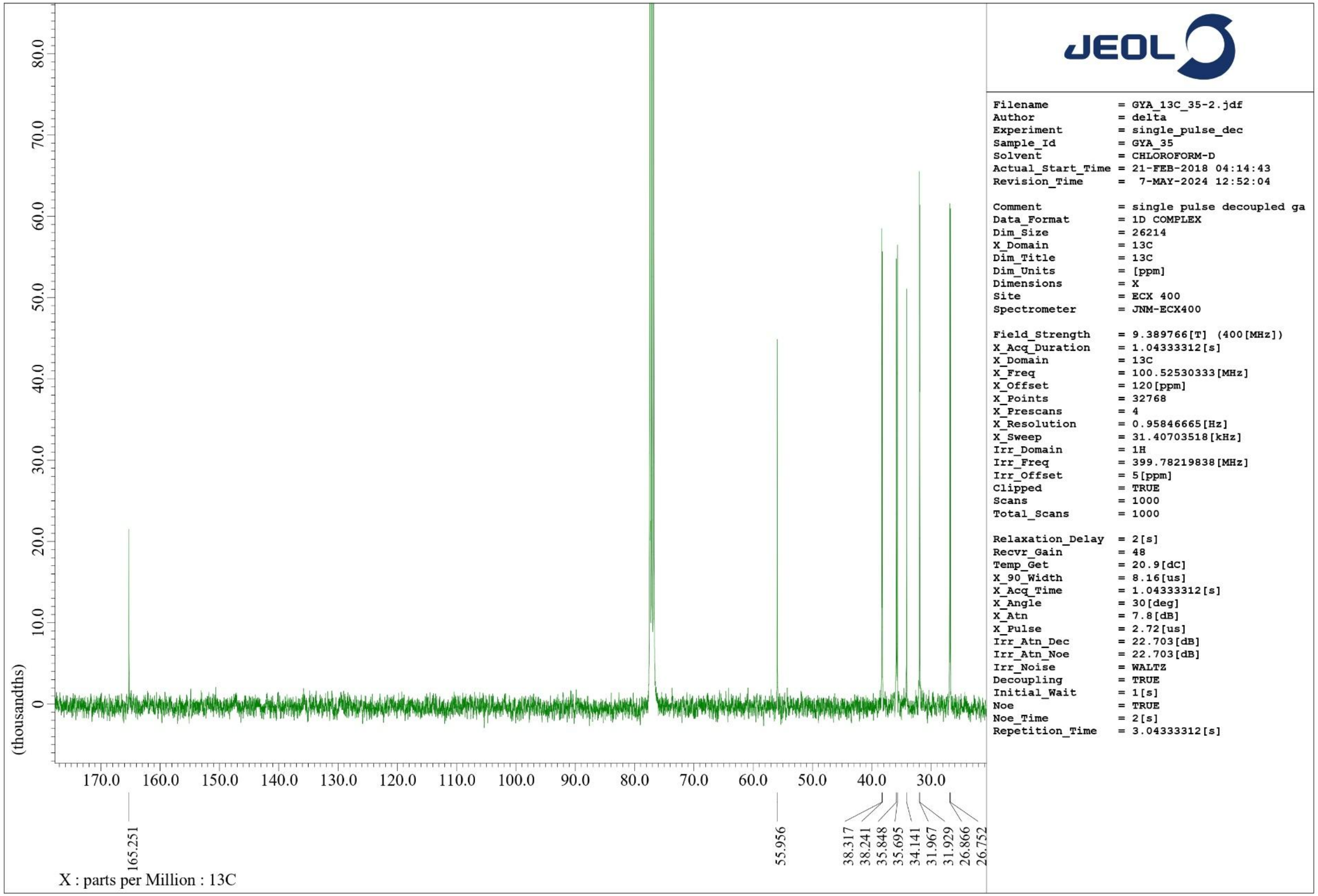
JEOL
Filename = GYA_13C_35-2.jdf
Author = delta
Experiment = single_pulse_dec
Sample_Id = GYA_35
Solvent = CHLOROFORM-D
Actual_Start_Time = 21-FEB-2018 04:14:43
Revision_Time = 7-MAY-2024 12:52:04
Comment = single pulse decoupled ga
Data_Format = 1D COMPLEX
Dim_Size = 26214
X_Domain = 13C
Dim_Title = 13C
Dim_Units = [ppm]
Dimensions = X
Site = ECX 400
Spectrometer = JNM-ECX400
Field_Strength = 9.389766[T] (400[MHz])
X_Acq_Duration = 1.04333312[s]
X_Domain = 13C
X_Freq = 100.52530333[MHz]
X_Offset = 120[ppm]
X_Points = 32768
X_Prescans = 4
X_Resolution = 0.95846665[Hz]
X_Sweep = 31.40703518[kHz]
Irr_Domain = 1H
Irr_Freq = 399.78219838[MHz]
Irr_Offset = 5[ppm]
Clipped = TRUE
Scans = 1000
Total_Scans = 1000
Relaxation_Delay = 2[s]
Recvr_Gain = 48
Temp_Get = 20.9[dC]
X_90_Width = 8.16[us]
X_Acq_Time = 1.04333312[s]
X_Angle = 30[deg]
X_Atn = 7.8[dB]
X_Pulse = 2.72[us]
Irr_Atn_Dec = 22.703[dB]
Irr_Atn_Noe = 22.703[dB]
Irr_Noise = WALTZ
Decoupling = TRUE
Initial_Wait = 1[s]
Noe = TRUE
Noe_Time = 2[s]
Repetition_Time = 3.04333312[s]
(thousandths)
80.0
70.0
60.0
50.0
40.0
30.0
20.0
10.0
0
170.0 160.0 150.0 140.0 130.0 120.0 110.0 100.0 90.0 80.0 70.0 60.0 50.0 40.0 30.0
165.251
55.956
38.317
38.241
35.848
35.695
34.141
31.967
31.929
26.866
26.752
X : parts per Million : 13C

3.2. NMR spectra of 5-azidotricyclo[4.3.1.1^{3,8}]undecane-4-one oxime (**4**)

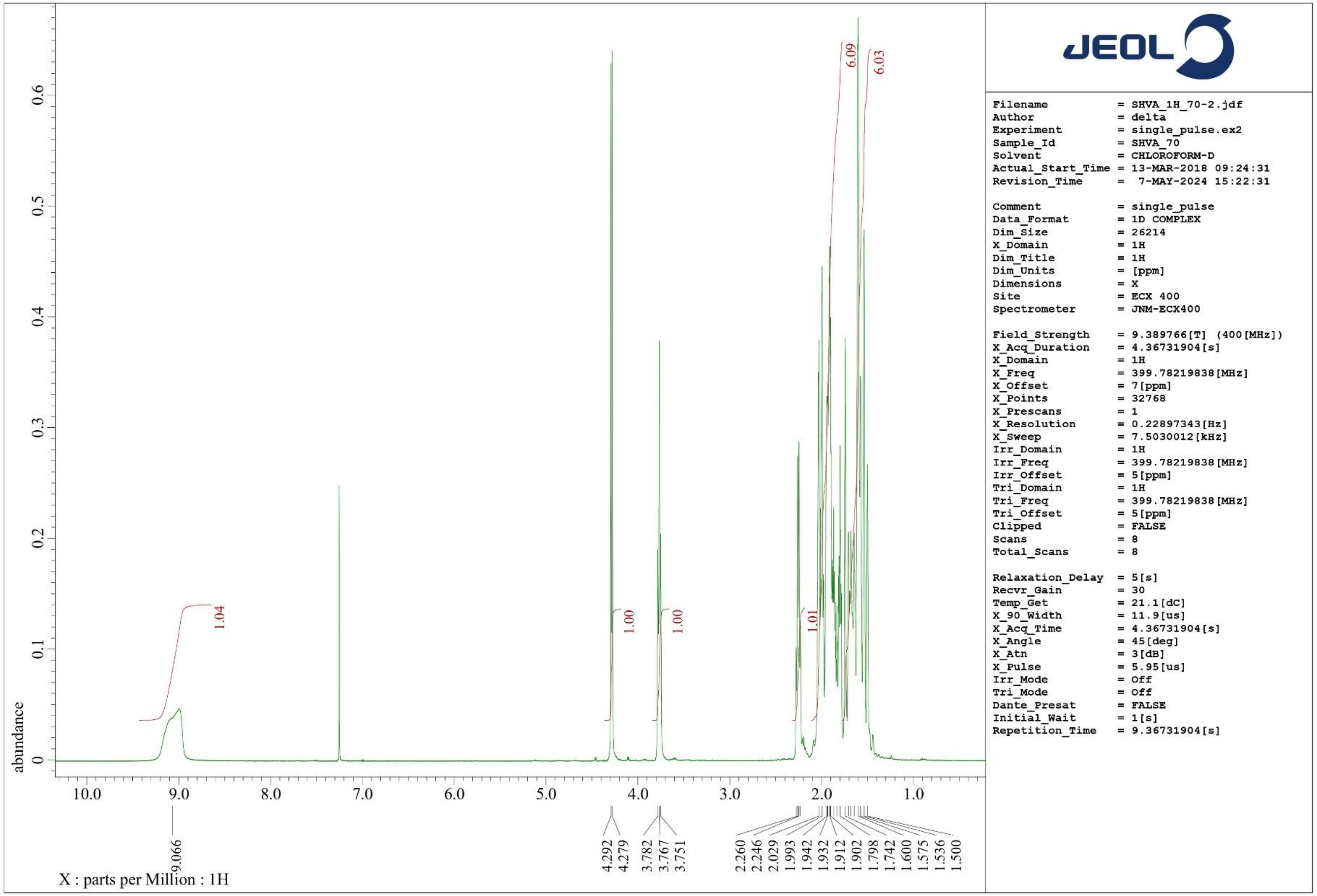

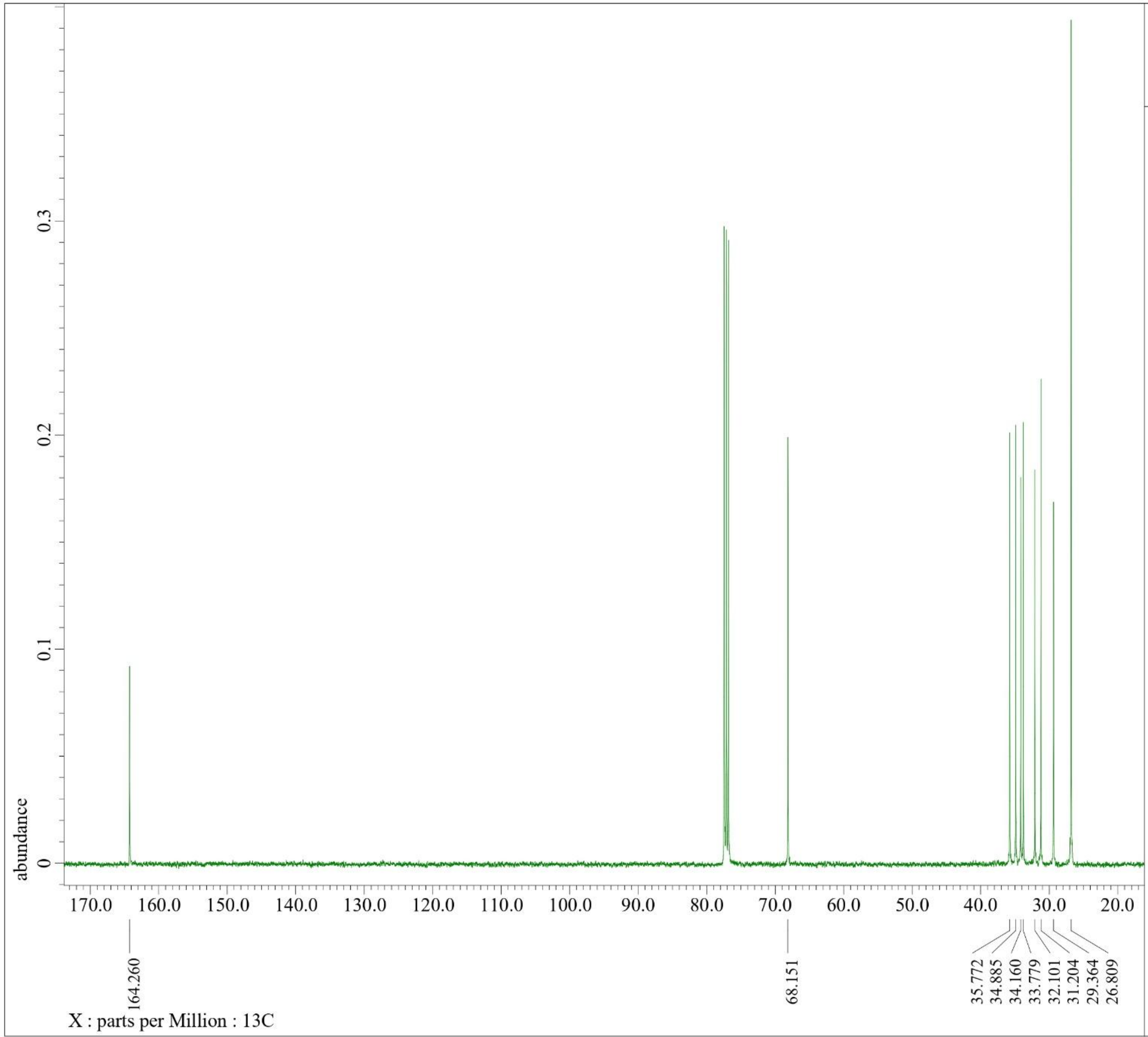


```
Filename          = SHVA_13C_70-2.jdf
Author            = delta
Experiment        = single_pulse_dec
Sample_Id         = SHVA_70
Solvent           = CHLOROFORM-D
Actual_Start_Time = 13-MAR-2018 09:28:13
Revision_Time     =  7-MAY-2024 15:19:29

Comment           = single pulse decoupled ga
Data_Format       = 1D COMPLEX
Dim_Size          = 26214
X_Domain          = 13C
Dim_Title         = 13C
Dim_Units         = [ppm]
Dimensions        = X
Site              = ECX 400
Spectrometer      = JNM-ECX400

Field_Strength    = 9.389766[T] (400[MHz])
X_Acq_Duration    = 1.04333312[s]
X_Domain          = 13C
X_Freq            = 100.52530333[MHz]
X_Offset          = 120[ppm]
X_Points          = 32768
X_Prescans        = 4
X_Resolution      = 0.95846665[Hz]
X_Sweep           = 31.40703518[kHz]
Irr_Domain        = 1H
Irr_Freq          = 399.78219838[MHz]
Irr_Offset        = 5[ppm]
Clipped           = FALSE
Scans             = 1000
Total_Scans       = 1000

Relaxation_Delay  = 2[s]
Recvr_Gain        = 46
Temp_Get          = 21.1[dC]
X_90_Width        = 8.16[us]
X_Acq_Time        = 1.04333312[s]
X_Angle           = 30[deg]
X_Atn             = 7.8[dB]
X_Pulse           = 2.72[us]
Irr_Atn_Dec       = 22.703[dB]
Irr_Atn_Noe       = 22.703[dB]
Irr_Noise         = WALTZ
Decoupling        = TRUE
Initial_Wait      = 1[s]
Noe               = TRUE
Noe_Time          = 2[s]
Repetition_Time   = 3.04333312[s]
```

3.3. NMR spectra of 5-(benzylamino)tricyclo[4.3.1.1^{3,8}]undecan-4-one oxime (**5**)

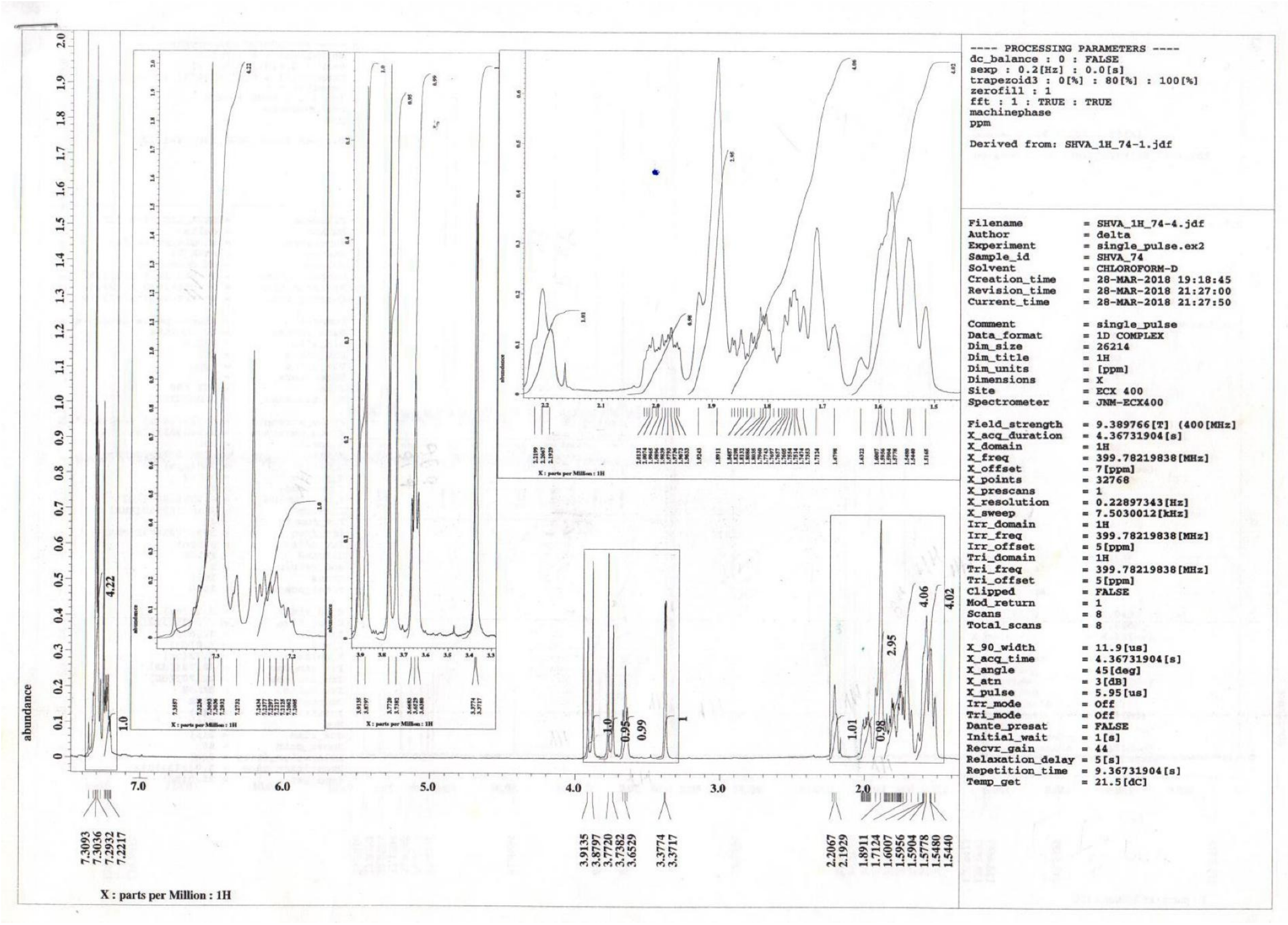

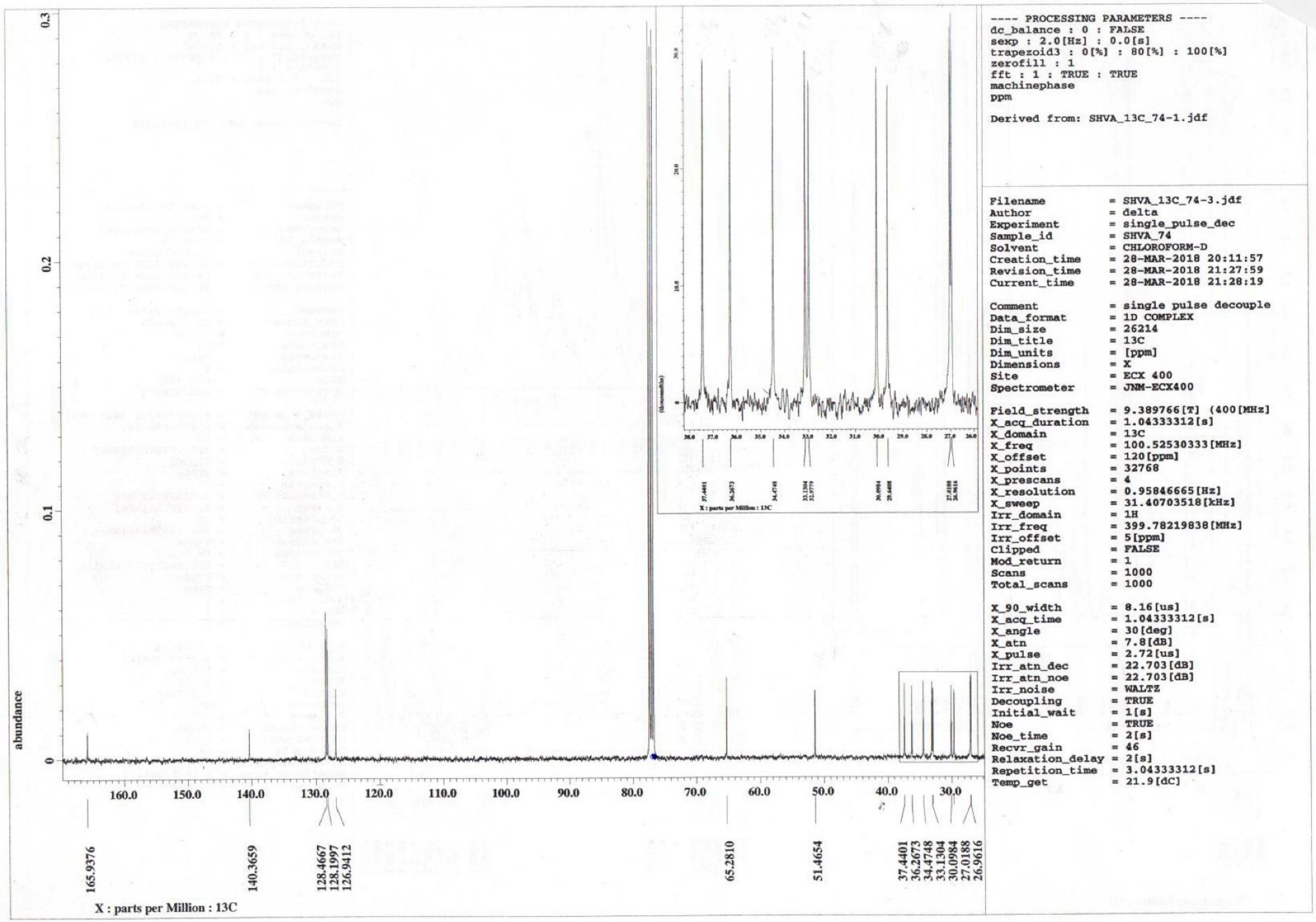
---- PROCESSING PARAMETERS ----
dc_balance : 0 : FALSE
sexp : 2.0[Hz] : 0.0[s]
trapezoid3 : 0[%] : 80[%] : 100[%]
zerofill : 1
fft : 1 : TRUE : TRUE
machinephase
ppm
Derived from: SHVA_13C_74-1.jdf
Filename = SHVA_13C_74-3.jdf
Author = delta
Experiment = single_pulse_dec
Sample_id = SHVA_74
Solvent = CHLOROFORM-D
Creation_time = 28-MAR-2018 20:11:57
Revision_time = 28-MAR-2018 21:27:59
Current_time = 28-MAR-2018 21:28:19
Comment = single pulse decouple
Data_format = 1D COMPLEX
Dim_size = 26214
Dim_title = 13C
Dim_units = [ppm]
Dimensions = X
Site = ECX 400
Spectrometer = JNM-ECX400
Field_strength = 9.389766[T] (400[MHz]
X_acq_duration = 1.04333312[s]
X_domain = 13C
X_freq = 100.52530333[MHz]
X_offset = 120[ppm]
X_points = 32768
X_prescans = 4
X_resolution = 0.95846665[Hz]
X_sweep = 31.40703518[kHz]
Irr_domain = 1H
Irr_freq = 399.78219838[MHz]
Irr_offset = 5[ppm]
Clipped = FALSE
Mod_return = 1
Scans = 1000
Total_scans = 1000
X_90_width = 8.16[us]
X_acq_time = 1.04333312[s]
X_angle = 30[deg]
X_atn = 7.8[dB]
X_pulse = 2.72[us]
Irr_atn_dec = 22.703[dB]
Irr_atn_noe = 22.703[dB]
Irr_noise = WALTZ
Decoupling = TRUE
Initial_wait = 1[s]
Noe = TRUE
Noe_time = 2[s]
Recvr_gain = 46
Relaxation_delay = 2[s]
Repetition_time = 3.04333312[s]
Temp_get = 21.9[dC]
abundance
0.3
0.2
0.1
0
160.0
150.0
140.0
130.0
120.0
110.0
100.0
90.0
80.0
70.0
60.0
50.0
40.0
30.0
165.9376
140.3659
128.4667
128.1997
126.9412
65.2810
51.4654
37.4401
36.2673
34.4748
33.1304
30.0984
27.0188
26.9616
X : parts per Million : 13C

3.4. NMR spectra of tricyclo[4.3.1.1^{3,8}]undecane-4-amine hydrochloride (**6**)

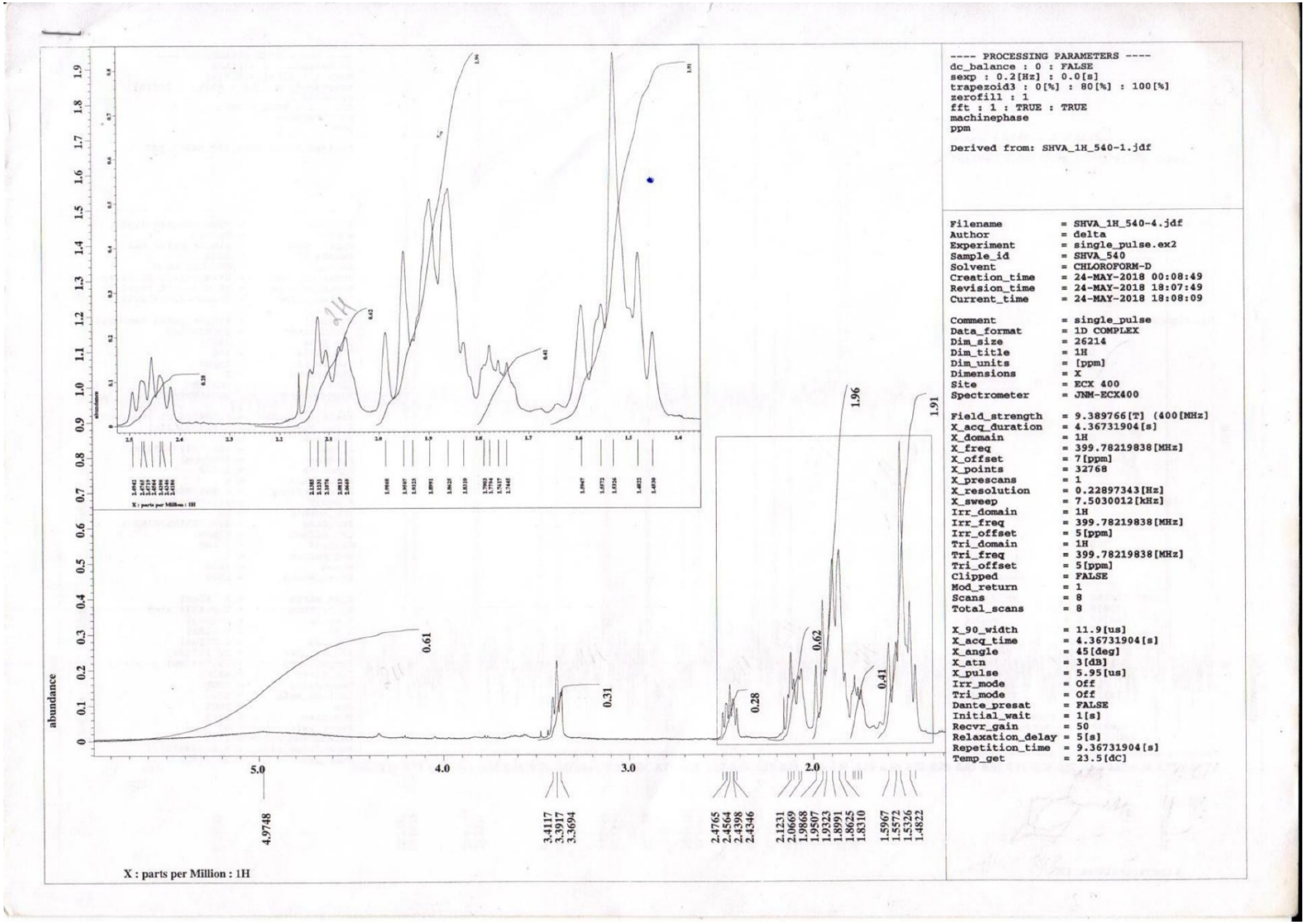

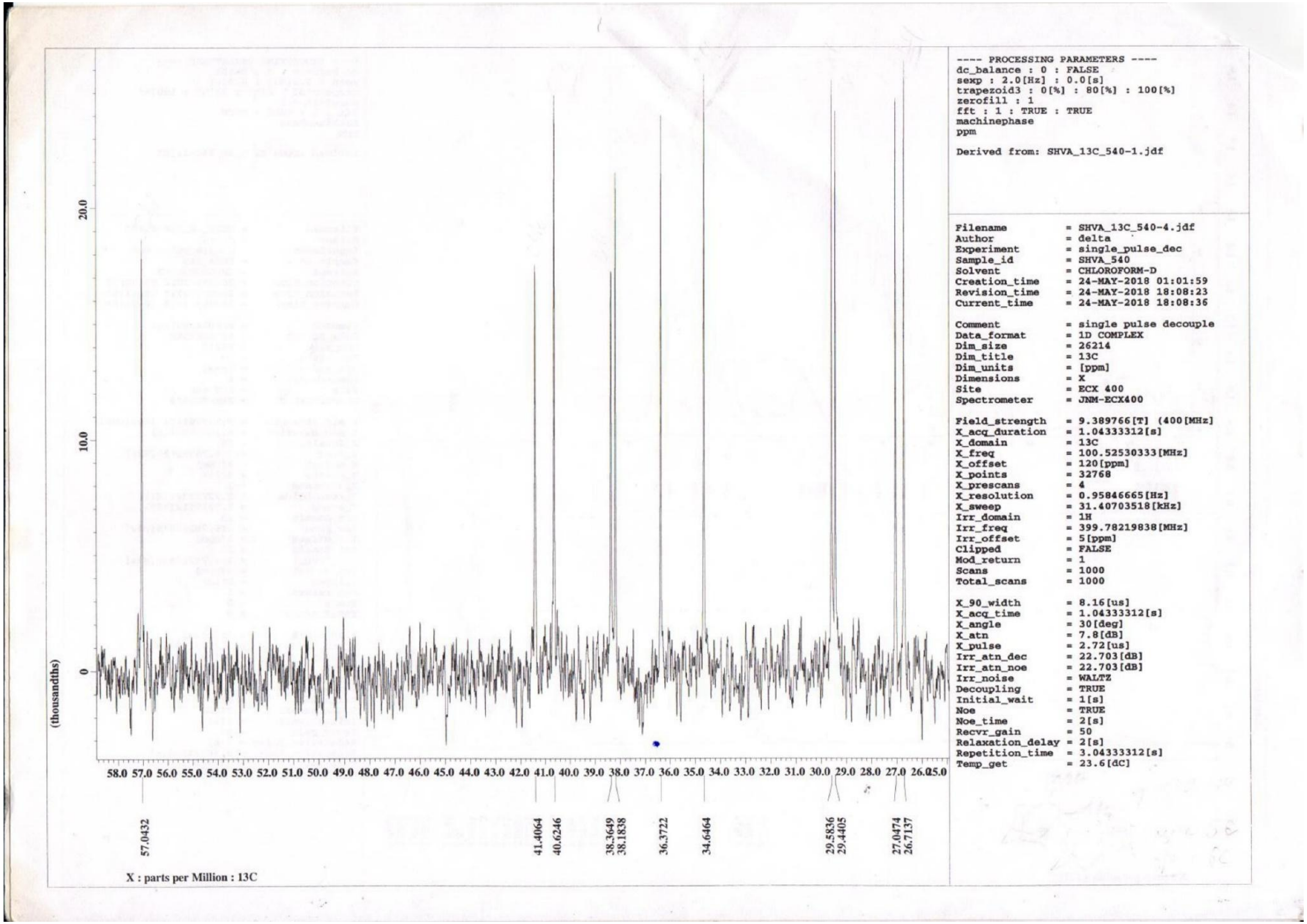
---- PROCESSING PARAMETERS ----
dc_balance : 0 : FALSE
sexp : 2.0[Hz] : 0.0[s]
trapezoid3 : 0[%] : 80[%] : 100[%]
zerofill : 1
fft : 1 : TRUE : TRUE
machinephase
ppm
Derived from: SHVA_13C_540-1.jdf
Filename = SHVA_13C_540-4.jdf
Author = delta
Experiment = single_pulse_dec
Sample_id = SHVA_540
Solvent = CHLOROFORM-D
Creation_time = 24-MAY-2018 01:01:59
Revision_time = 24-MAY-2018 18:08:23
Current_time = 24-MAY-2018 18:08:36
Comment = single pulse decouple
Data_format = 1D COMPLEX
Dim_size = 26214
Dim_title = 13C
Dim_units = [ppm]
Dimensions = X
Site = ECX 400
Spectrometer = JNM-ECX400
Field_strength = 9.389766[T] (400[MHz]
X_acq_duration = 1.04333312[s]
X_domain = 13C
X_freq = 100.52530333[MHz]
X_offset = 120[ppm]
X_points = 32768
X_prescans = 4
X_resolution = 0.95846665[Hz]
X_sweep = 31.40703518[kHz]
Irr_domain = 1H
Irr_freq = 399.78219838[MHz]
Irr_offset = 5[ppm]
Clipped = FALSE
Mod_return = 1
Scans = 1000
Total_scans = 1000
X_90_width = 8.16[us]
X_acq_time = 1.04333312[s]
X_angle = 30[deg]
X_atn = 7.8[dB]
X_pulse = 2.72[us]
Irr_atn_dec = 22.703[dB]
Irr_atn_noe = 22.703[dB]
Irr_noise = WALTZ
Decoupling = TRUE
Initial_wait = 1[s]
Noe = TRUE
Noe_time = 2[s]
Recvr_gain = 50
Relaxation_delay = 2[s]
Repetition_time = 3.04333312[s]
Temp_get = 23.6[dC]
(thousandths)
20.0
10.0
0
58.0 57.0 56.0 55.0 54.0 53.0 52.0 51.0 50.0 49.0 48.0 47.0 46.0 45.0 44.0 43.0 42.0 41.0 40.0 39.0 38.0 37.0 36.0 35.0 34.0 33.0 32.0 31.0 30.0 29.0 28.0 27.0 26.0 25.0
57.0432
41.4064
40.6246
38.3649
38.1838
36.3722
34.6464
29.5836
29.4405
27.0474
26.7137
X : parts per Million : 13C

3.5. NMR spectra of 5-azidotricyclo[4.3.1.1^{3,8}]undecan-4-amine hydrochloride (**7**)

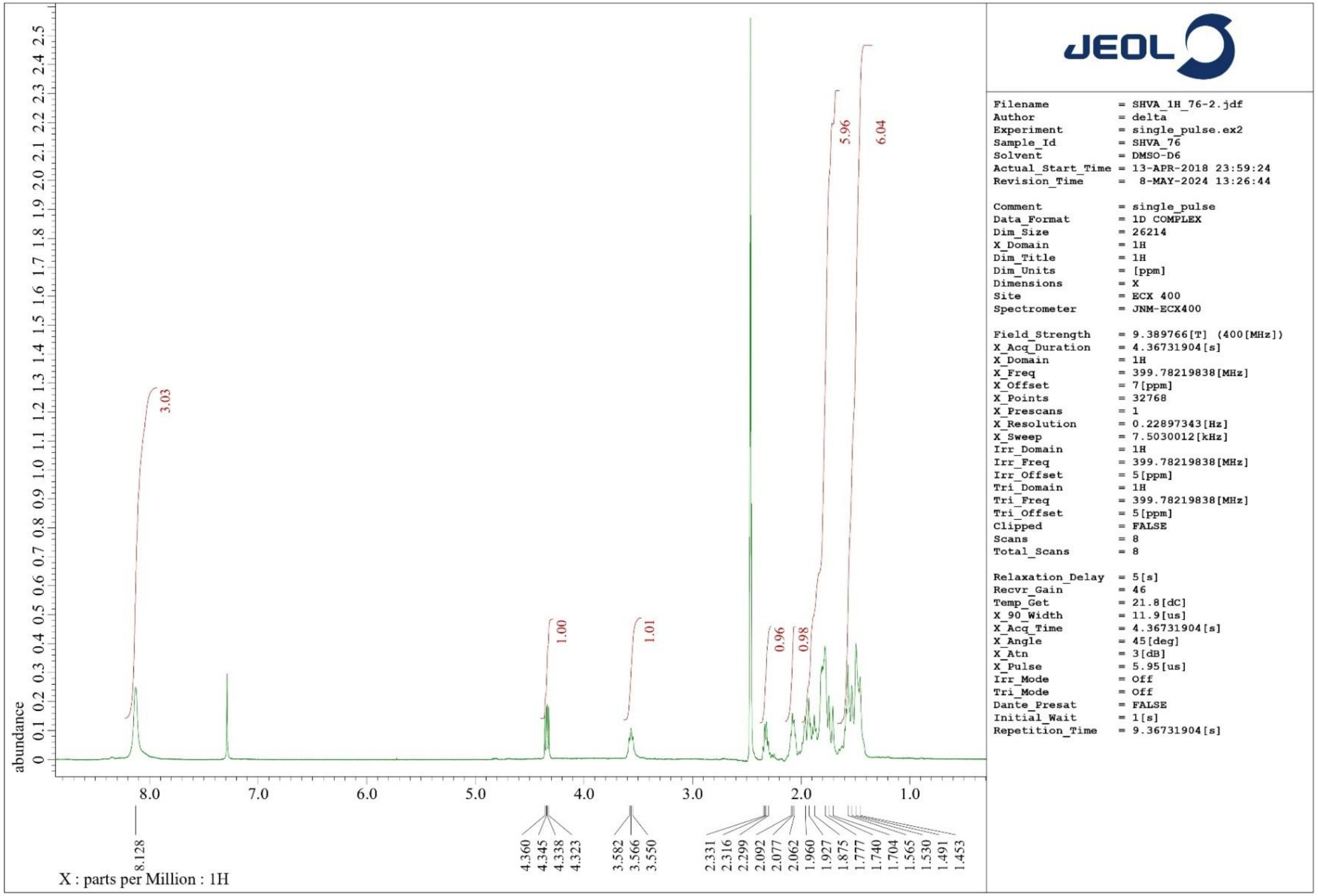

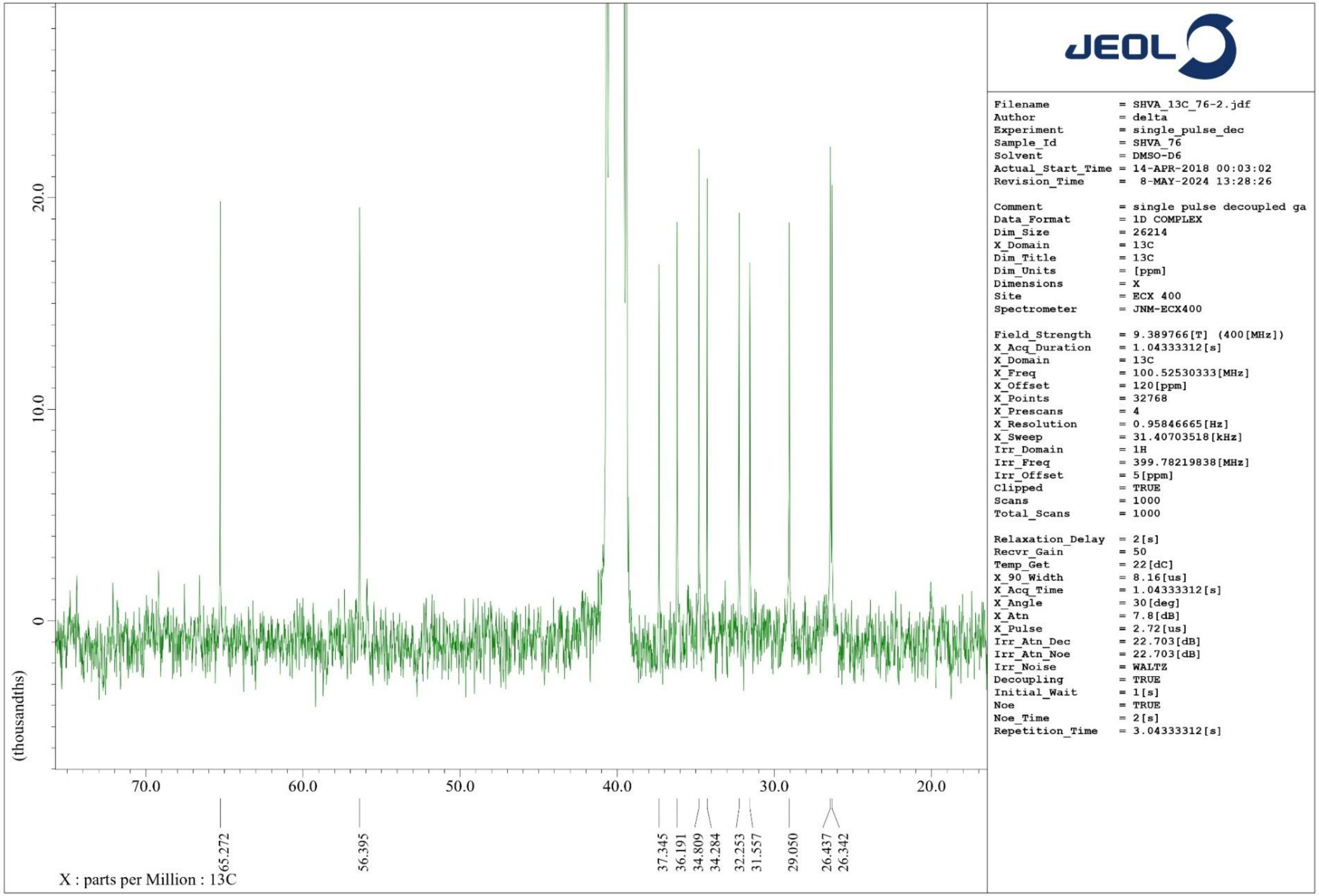
JEOL
Filename = SHVA_13C_76-2.jdf
Author = delta
Experiment = single_pulse_dec
Sample_Id = SHVA_76
Solvent = DMSO-D6
Actual_Start_Time = 14-APR-2018 00:03:02
Revision_Time = 8-MAY-2024 13:28:26
Comment = single pulse decoupled ga
Data_Format = 1D COMPLEX
Dim_Size = 26214
X_Domain = 13C
Dim_Title = 13C
Dim_Units = [ppm]
Dimensions = X
Site = ECX 400
Spectrometer = JNM-ECX400
Field_Strength = 9.389766[T] (400[MHz])
X_Acq_Duration = 1.04333312[s]
X_Domain = 13C
X_Freq = 100.52530333[MHz]
X_Offset = 120[ppm]
X_Points = 32768
X_Prescans = 4
X_Resolution = 0.95846665[Hz]
X_Sweep = 31.40703518[kHz]
Irr_Domain = 1H
Irr_Freq = 399.78219838[MHz]
Irr_Offset = 5[ppm]
Clipped = TRUE
Scans = 1000
Total_Scans = 1000
Relaxation_Delay = 2[s]
Recvr_Gain = 50
Temp_Get = 22[dC]
X_90_Width = 8.16[us]
X_Acq_Time = 1.04333312[s]
X_Angle = 30[deg]
X_Atn = 7.8[dB]
X_Pulse = 2.72[us]
Irr_Atn_Dec = 22.703[dB]
Irr_Atn_Noe = 22.703[dB]
Irr_Noise = WALTZ
Decoupling = TRUE
Initial_Wait = 1[s]
Noe = TRUE
Noe_Time = 2[s]
Repetition_Time = 3.04333312[s]
(thousandths)
20.0
10.0
0
70.0
60.0
50.0
40.0
30.0
20.0
65.272
56.395
37.345
36.191
34.809
34.284
32.253
31.557
29.050
26.437
26.342
X : parts per Million : 13C

3.6. NMR spectra of *trans*/*cis*-4,5-diaminohomoadamantane dihydrochloride **8a,b**

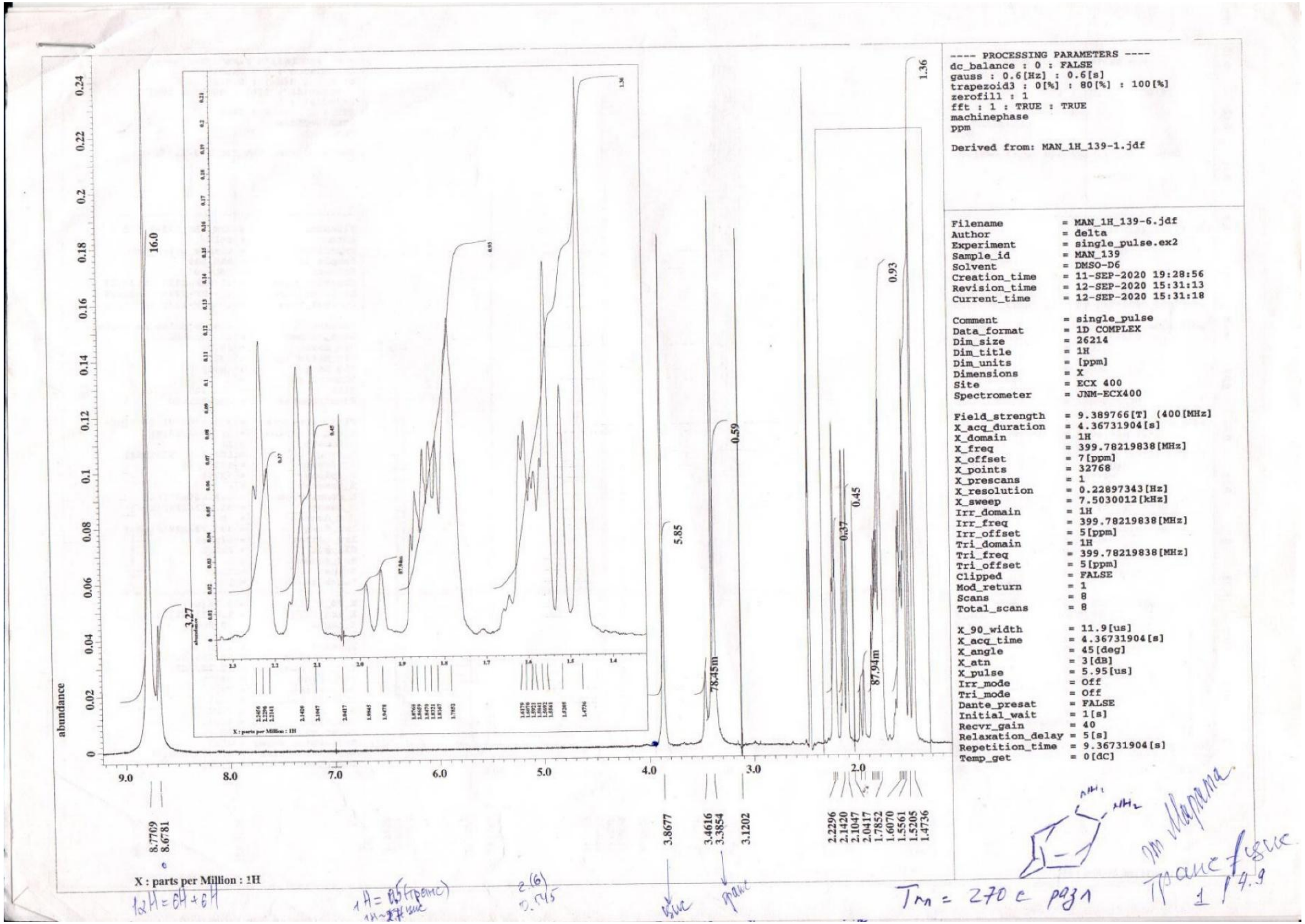

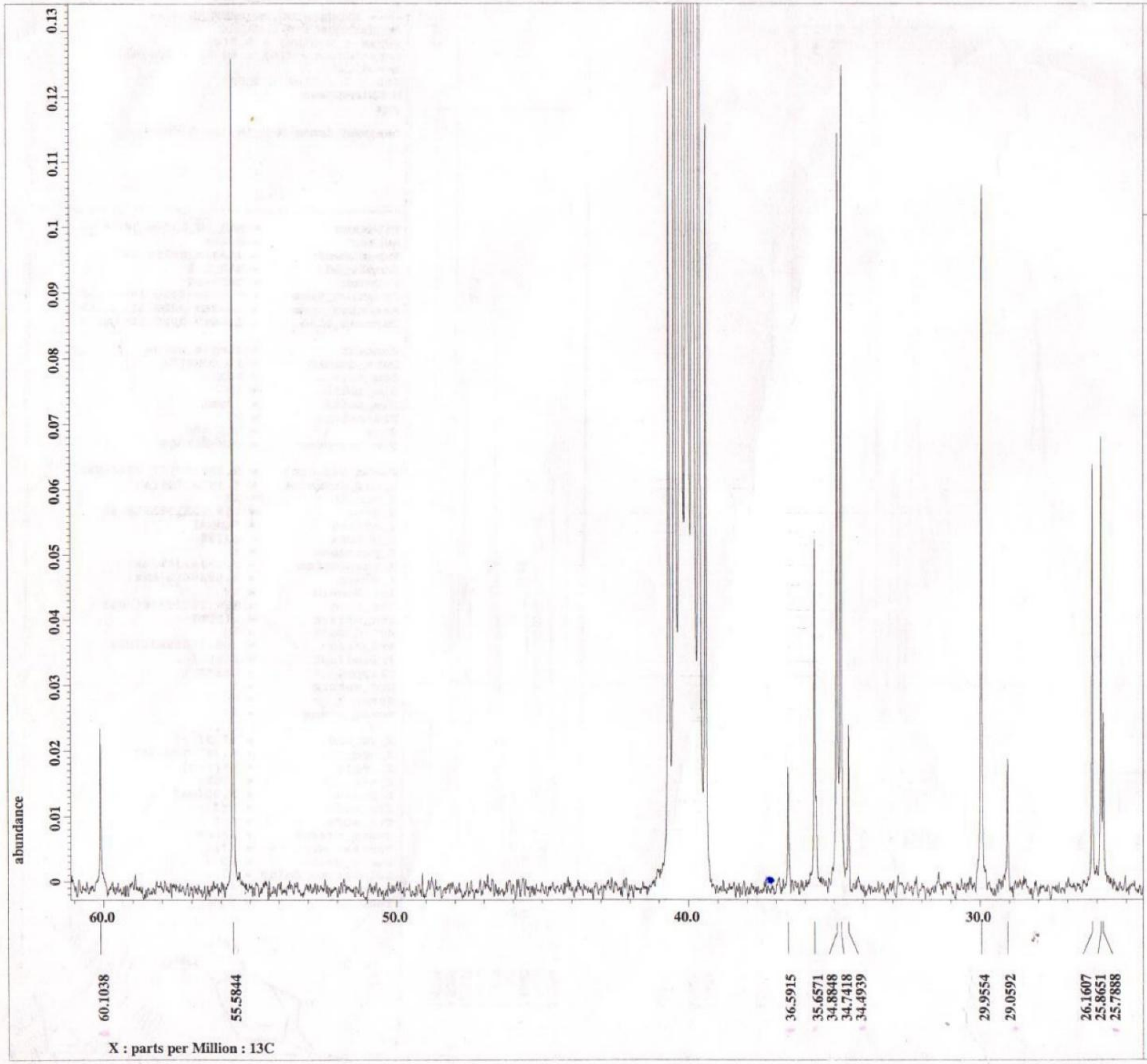


```
---- PROCESSING PARAMETERS ----
dc_balance : 0 : FALSE
sexp : 2.0[Hz] : 0.0[s]
trapezoid3 : 0[%] : 80[%] : 100[%]
zerofill : 1
fft : 1 : TRUE : TRUE
machinephase
ppm

Derived from: MAN_13C_139-1.jdf

Filename           = MAN_13C_139-3.jdf
Author             = delta
Experiment         = single_pulse_dec
Sample_id          = MAN_139
Solvent            = DMSO-D6
Creation_time      = 11-SEP-2020 20:22:57
Revision_time      = 12-SEP-2020 15:31:39
Current_time       = 12-SEP-2020 15:31:54

Comment            = single pulse decouple
Data_format        = 1D COMPLEX
Dim_size           = 26214
Dim_title          = 13C
Dim_units          = [ppm]
Dimensions         = X
Site               = ECX 400
Spectrometer       = JNM-ECX400

Field_strength     = 9.389766[T] (400[MHz]
X_acq_duration     = 1.04333312[s]
X_domain           = 13C
X_freq             = 100.52530333[MHz]
X_offset           = 120[ppm]
X_points           = 32768
X_prescans         = 4
X_resolution       = 0.95846665[Hz]
X_sweep            = 31.40703518[kHz]
Irr_domain         = 1H
Irr_freq           = 399.78219838[MHz]
Irr_offset         = 5[ppm]
Clipped            = FALSE
Mod_return         = 1
Scans              = 1000
Total_scans        = 1000

X_90_width         = 8.16[us]
X_acq_time         = 1.04333312[s]
X_angle            = 30[deg]
X_atn              = 7.8[dB]
X_pulse            = 2.72[us]
Irr_atn_dec        = 22.703[dB]
Irr_atn_noe        = 22.703[dB]
Irr_noise          = WALTZ
Decoupling         = TRUE
Initial_wait       = 1[s]
Noe                = TRUE
Noe_time           = 2[s]
Recvr_gain         = 48
Relaxation_delay   = 2[s]
Repetition_time    = 3.04333312[s]
Temp_get           = 0[dC]
```

3.7. NMR spectra of *trans*-4,5-diaminohomoadamantane dihydrochloride (**8a**)

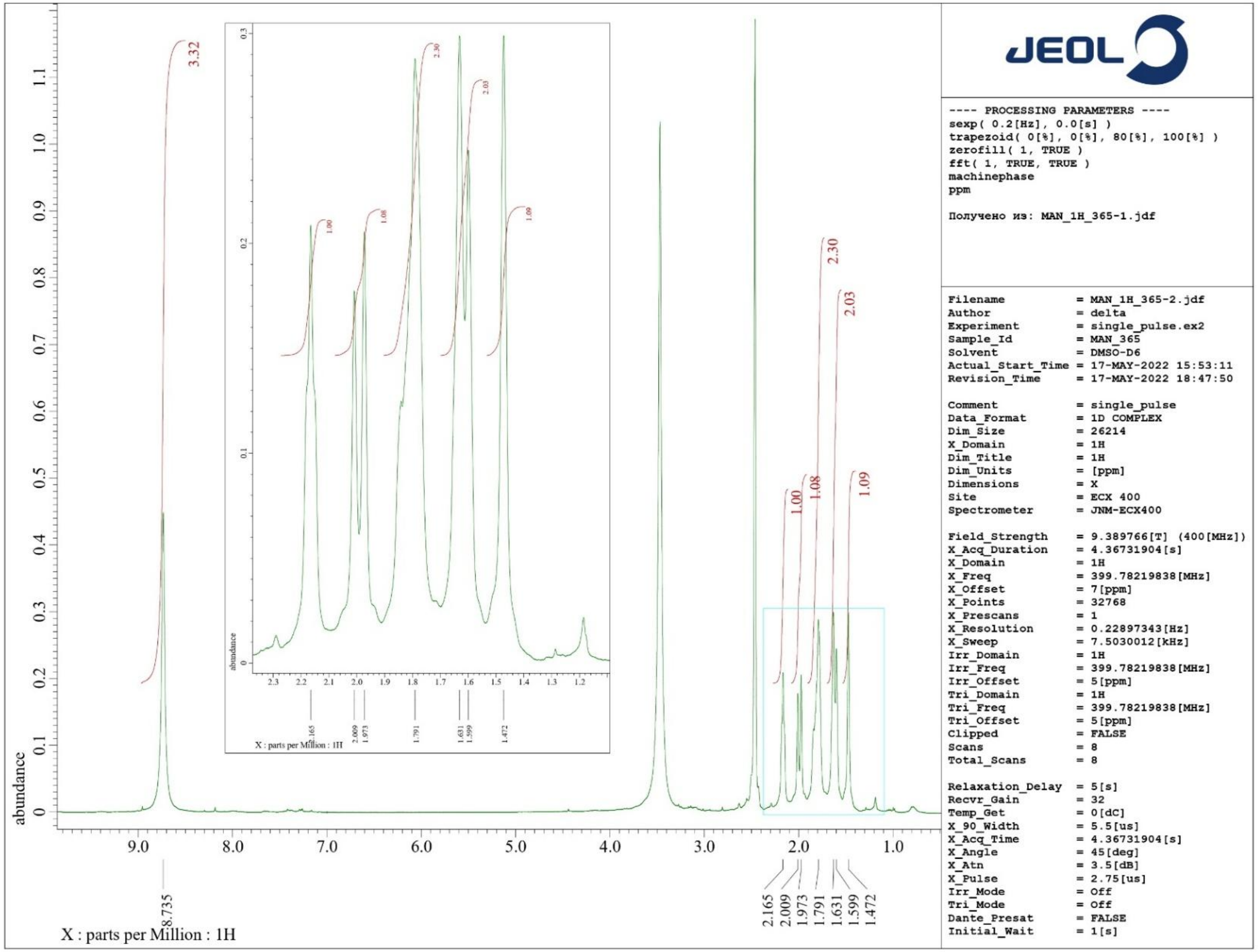

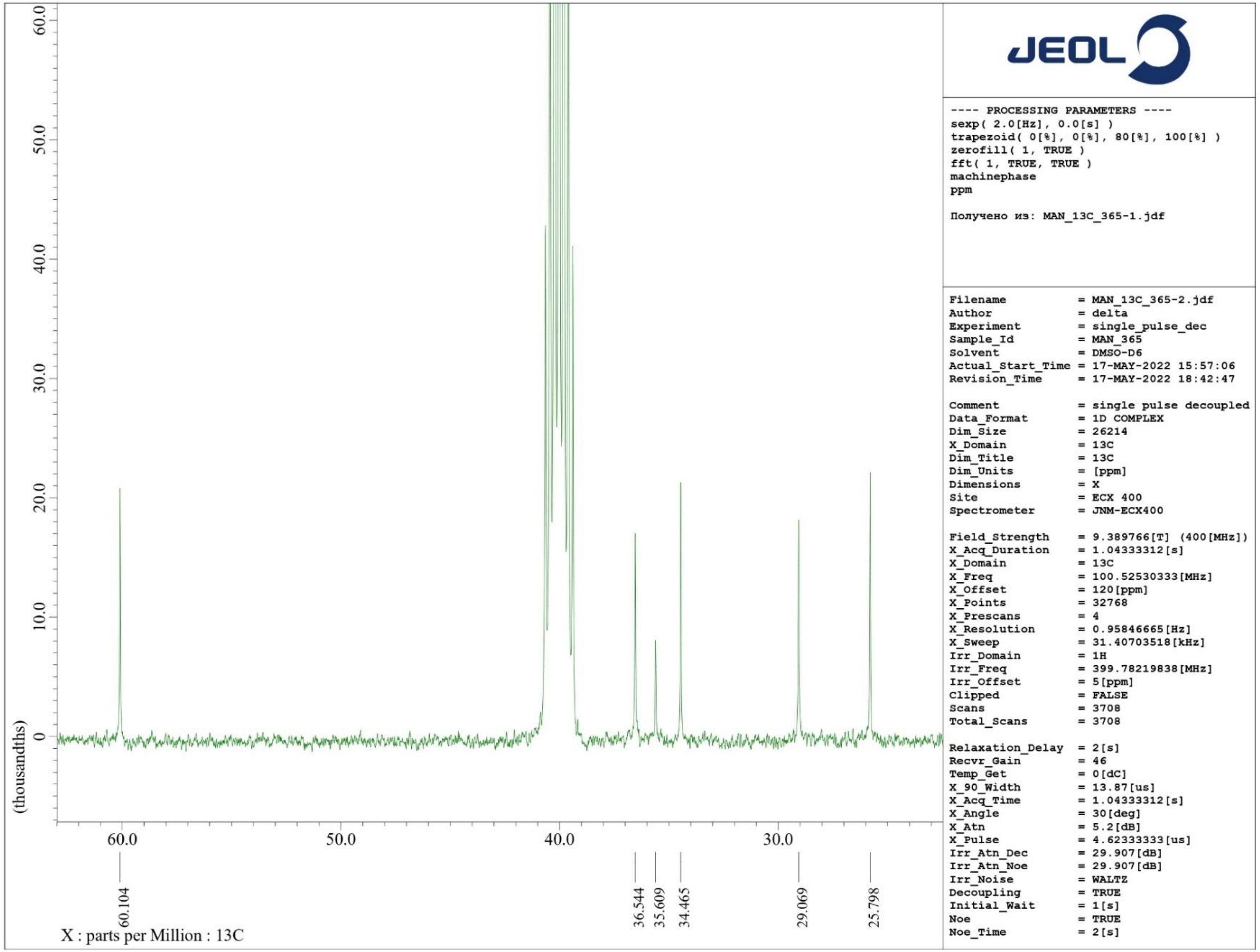


```
---- PROCESSING PARAMETERS ----
sexp( 2.0[Hz], 0.0[s] )
trapezoid( 0[%], 0[%], 80[%], 100[%] )
zerofill( 1, TRUE )
fft( 1, TRUE, TRUE )
machinephase
ppm

Получено из: MAN_13C_365-1.jdf
```

```
Filename           = MAN_13C_365-2.jdf
Author             = delta
Experiment         = single_pulse_dec
Sample_Id          = MAN_365
Solvent            = DMSO-D6
Actual_Start_Time  = 17-MAY-2022 15:57:06
Revision_Time      = 17-MAY-2022 18:42:47

Comment            = single pulse decoupled
Data_Format        = 1D COMPLEX
Dim_Size           = 26214
X_Domain           = 13C
Dim_Title          = 13C
Dim_Units          = [ppm]
Dimensions         = X
Site               = ECX 400
Spectrometer       = JNM-ECX400

Field_Strength     = 9.389766[T] (400[MHz])
X_Acq_Duration     = 1.04333312[s]
X_Domain           = 13C
X_Freq             = 100.52530333[MHz]
X_Offset           = 120[ppm]
X_Points           = 32768
X_Prescans         = 4
X_Resolution       = 0.95846665[Hz]
X_Sweep            = 31.40703518[kHz]
Irr_Domain         = 1H
Irr_Freq           = 399.78219838[MHz]
Irr_Offset         = 5[ppm]
Clipped            = FALSE
Scans              = 3708
Total_Scans        = 3708

Relaxation_Delay   = 2[s]
Recvr_Gain         = 46
Temp_Get           = 0[dC]
X_90_Width         = 13.87[us]
X_Acq_Time         = 1.04333312[s]
X_Angle            = 30[deg]
X_Atn              = 5.2[dB]
X_Pulse            = 4.62333333[us]
Irr_Atn_Dec        = 29.907[dB]
Irr_Atn_Noe        = 29.907[dB]
Irr_Noise          = WALTZ
Decoupling         = TRUE
Initial_Wait       = 1[s]
Noe                = TRUE
Noe_Time           = 2[s]
```

3.8. NMR spectra of *tert*-butyl 5-azatetracyclo[5.3.1.1^{3,9}.0^{4,6}]dodecane-5-carboxylate (**10**)

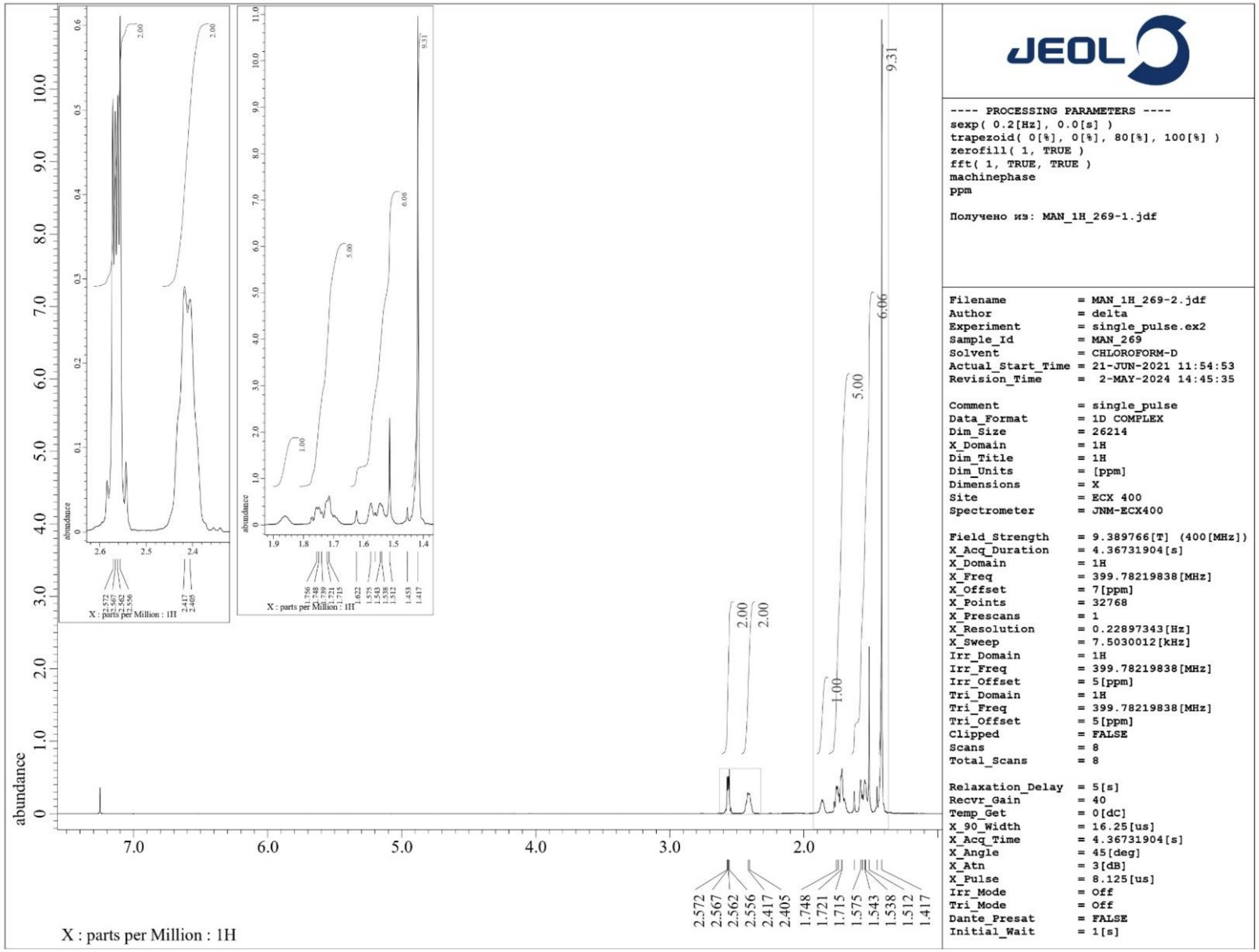

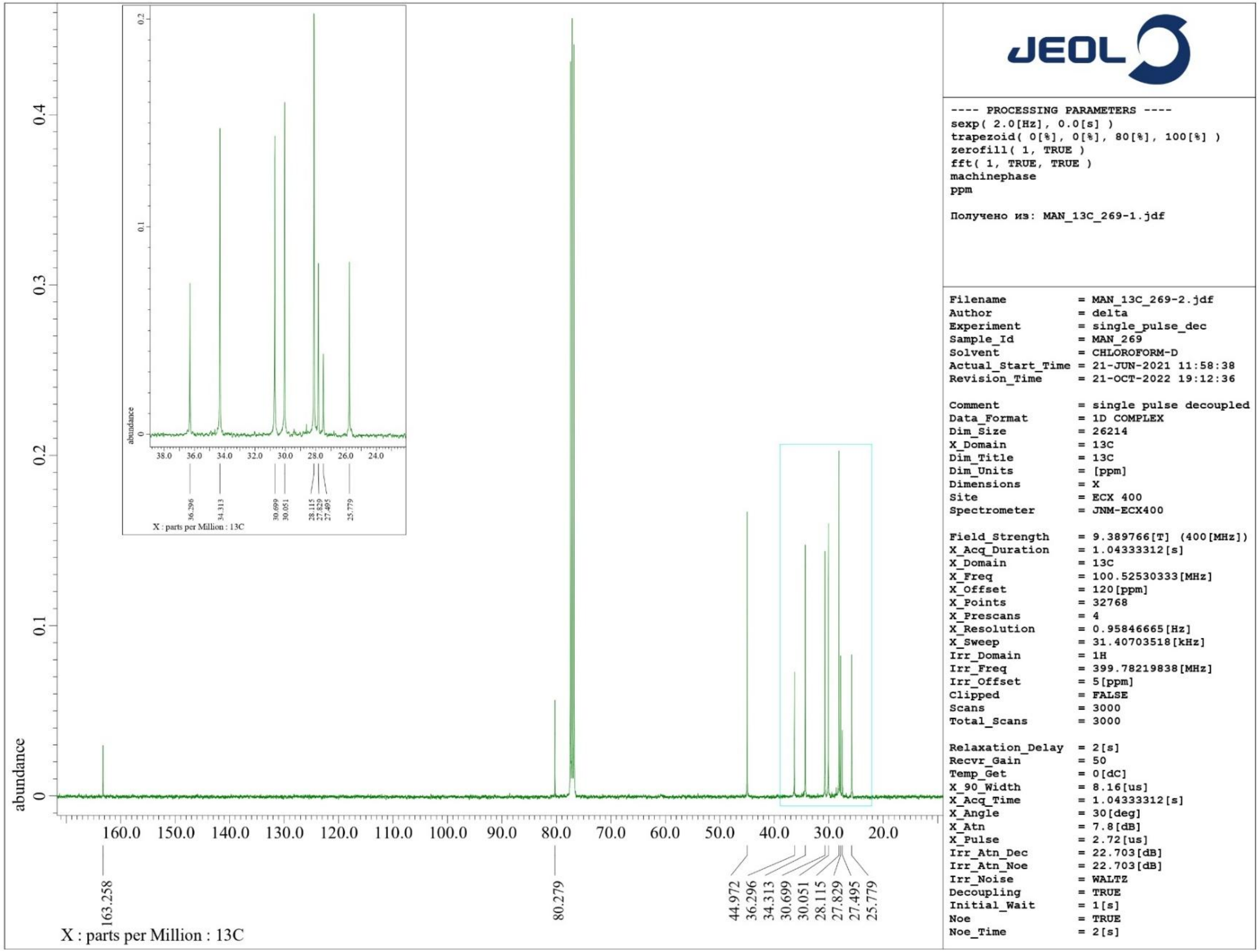
abundance
0
0.1
0.2
0.3
0.4
160.0 150.0 140.0 130.0 120.0 110.0 100.0 90.0 80.0 70.0 60.0 50.0 40.0 30.0 20.0
163.258
80.279
44.972
36.296
34.313
30.699
30.051
28.115
27.829
27.495
25.779
X : parts per Million : 13C
38.0 36.0 34.0 32.0 30.0 28.0 26.0 24.0
X : parts per Million : 13C
JEOL
---- PROCESSING PARAMETERS ----
sexp(2.0[Hz], 0.0[s])
trapezoid(0[%], 0[%], 80[%], 100[%])
zerofill(1, TRUE)
fft(1, TRUE, TRUE)
machinephase
ppm
Получено из: MAN_13C_269-1.jdf
Filename = MAN_13C_269-2.jdf
Author = delta
Experiment = single_pulse_dec
Sample_Id = MAN_269
Solvent = CHLOROFORM-D
Actual_Start_Time = 21-JUN-2021 11:58:38
Revision_Time = 21-OCT-2022 19:12:36
Comment = single pulse decoupled
Data_Format = 1D COMPLEX
Dim_Size = 26214
X_Domain = 13C
Dim_Title = 13C
Dim_Units = [ppm]
Dimensions = X
Site = ECX 400
Spectrometer = JNM-ECX400
Field_Strength = 9.389766[T] (400[MHz])
X_Acq_Duration = 1.04333312[s]
X_Domain = 13C
X_Freq = 100.52530333[MHz]
X_Offset = 120[ppm]
X_Points = 32768
X_Prescans = 4
X_Resolution = 0.95846665[Hz]
X_Sweep = 31.40703518[kHz]
Irr_Domain = 1H
Irr_Freq = 399.78219838[MHz]
Irr_Offset = 5[ppm]
Clipped = FALSE
Scans = 3000
Total_Scans = 3000
Relaxation_Delay = 2[s]
Recvr_Gain = 50
Temp_Get = 0[dC]
X_90_Width = 8.16[us]
X_Acq_Time = 1.04333312[s]
X_Angle = 30[deg]
X_Atn = 7.8[dB]
X_Pulse = 2.72[us]
Irr_Atn_Dec = 22.703[dB]
Irr_Atn_Noe = 22.703[dB]
Irr_Noise = WALTZ
Decoupling = TRUE
Initial_Wait = 1[s]
Noe = TRUE
Noe_Time = 2[s]

3.9. NMR spectra of *cis*-5-*p*-tosyl-5-azatetracyclo[5.3.1.1^{3,9}.0^{4,6}]dodecane (**11**)

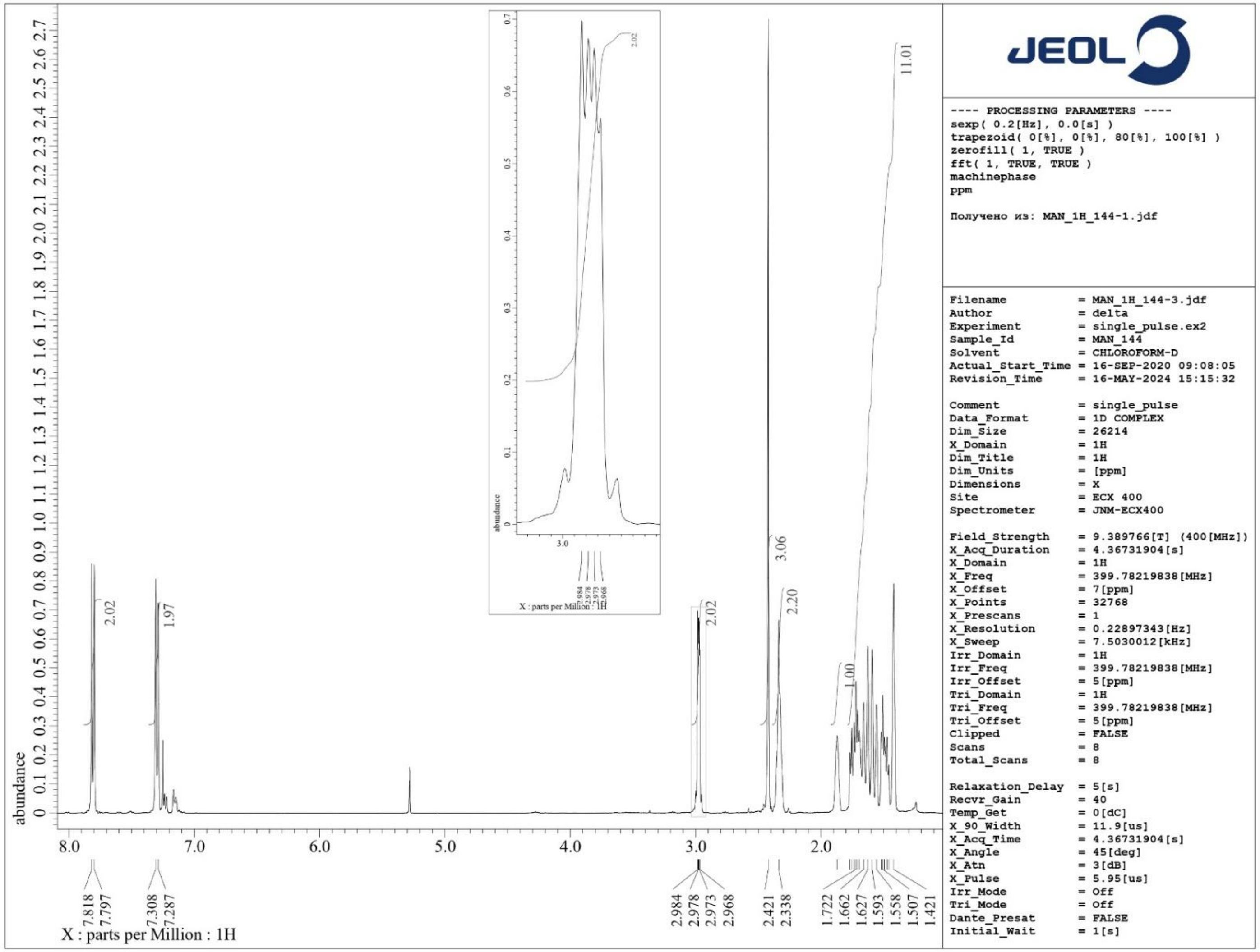

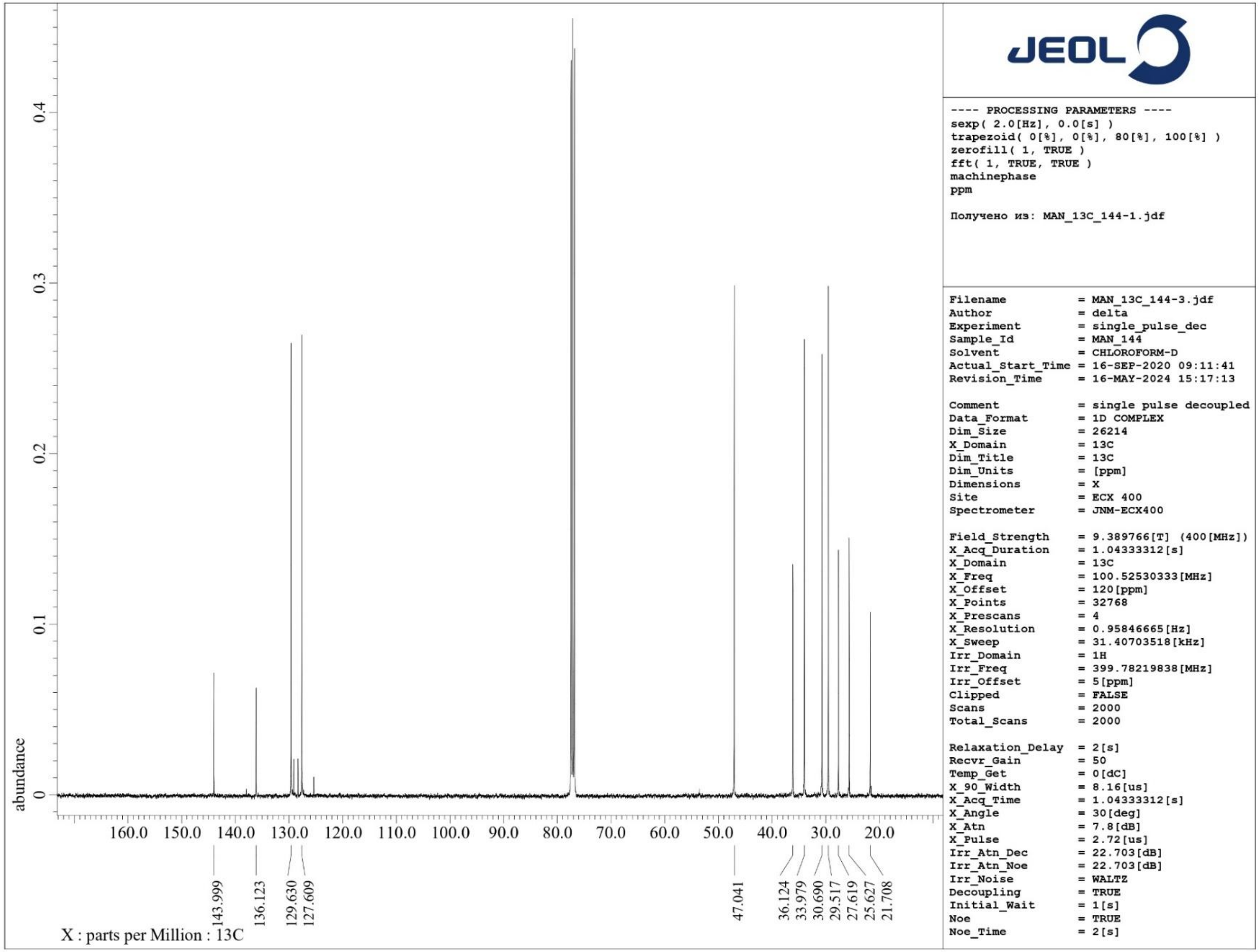
JEOL
---- PROCESSING PARAMETERS ----
sexp(2.0[Hz], 0.0[s])
trapezoid(0[%], 0[%], 80[%], 100[%])
zerofill(1, TRUE)
fft(1, TRUE, TRUE)
machinephase
ppm
Получено из: MAN_13C_144-1.jdf
Filename = MAN_13C_144-3.jdf
Author = delta
Experiment = single_pulse_dec
Sample_Id = MAN_144
Solvent = CHLOROFORM-D
Actual_Start_Time = 16-SEP-2020 09:11:41
Revision_Time = 16-MAY-2024 15:17:13
Comment = single pulse decoupled
Data_Format = 1D COMPLEX
Dim_Size = 26214
X_Domain = 13C
Dim_Title = 13C
Dim_Units = [ppm]
Dimensions = X
Site = ECX 400
Spectrometer = JNM-ECX400
Field_Strength = 9.389766[T] (400[MHz])
X_Acq_Duration = 1.04333312[s]
X_Domain = 13C
X_Freq = 100.52530333[MHz]
X_Offset = 120[ppm]
X_Points = 32768
X_Prescans = 4
X_Resolution = 0.95846665[Hz]
X_Sweep = 31.40703518[kHz]
Irr_Domain = 1H
Irr_Freq = 399.78219838[MHz]
Irr_Offset = 5[ppm]
Clipped = FALSE
Scans = 2000
Total_Scans = 2000
Relaxation_Delay = 2[s]
Recvr_Gain = 50
Temp_Get = 0[dC]
X_90_Width = 8.16[us]
X_Acq_Time = 1.04333312[s]
X_Angle = 30[deg]
X_Atn = 7.8[dB]
X_Pulse = 2.72[us]
Irr_Atn_Dec = 22.703[dB]
Irr_Atn_Noe = 22.703[dB]
Irr_Noise = WALTZ
Decoupling = TRUE
Initial_Wait = 1[s]
Noe = TRUE
Noe_Time = 2[s]
abundance
0.4
0.3
0.2
0.1
0
160.0 150.0 140.0 130.0 120.0 110.0 100.0 90.0 80.0 70.0 60.0 50.0 40.0 30.0 20.0
143.999
136.123
129.630
127.609
47.041
36.124
33.979
30.690
29.517
27.619
25.627
21.708
X : parts per Million : 13C

3.10. NMR spectra of *cis*-5-((trifluoromethyl)sulfonyl)-5-azatetracyclo[5.3.1.1^{3,9}.0^{4,6}]dodecane (**12**)

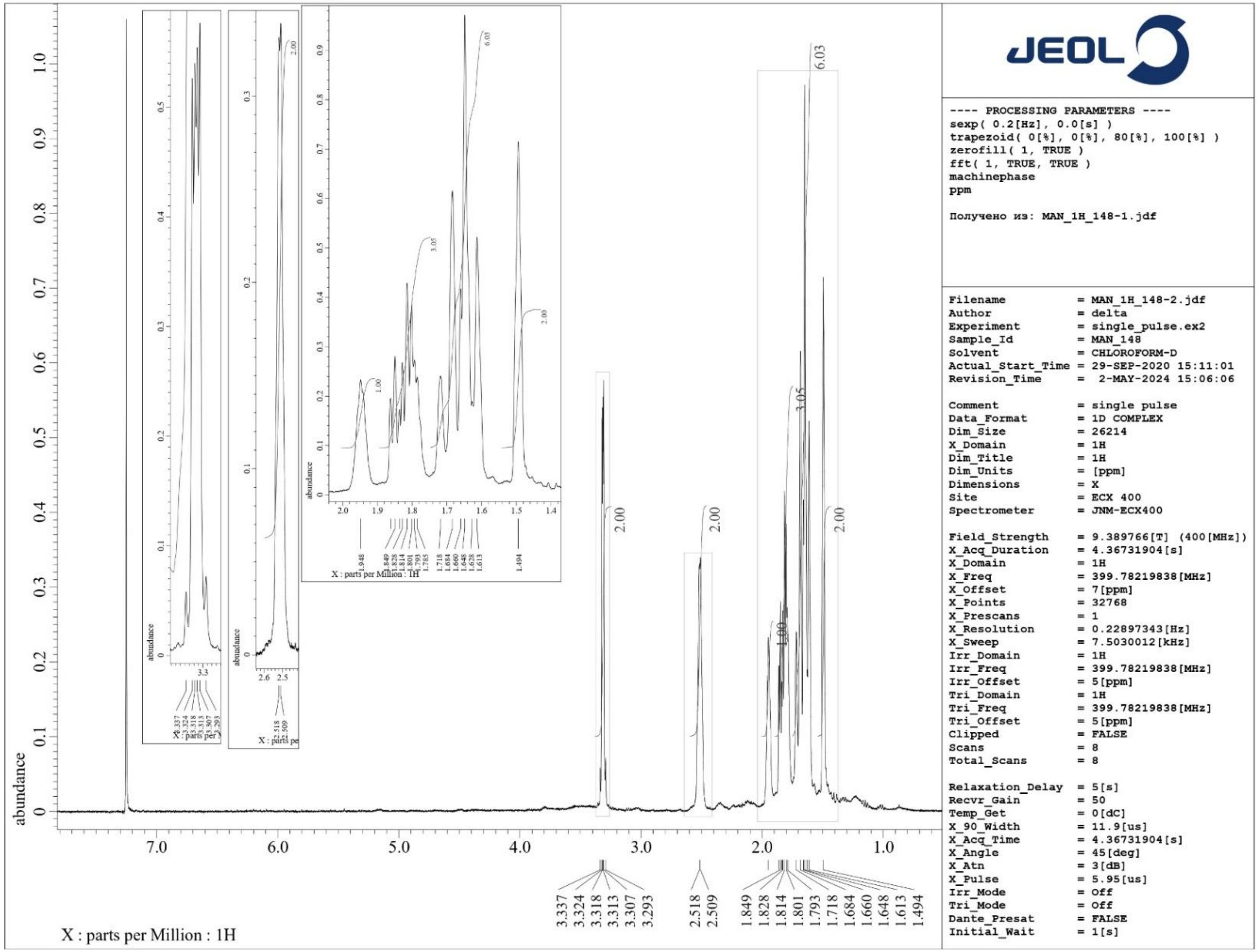

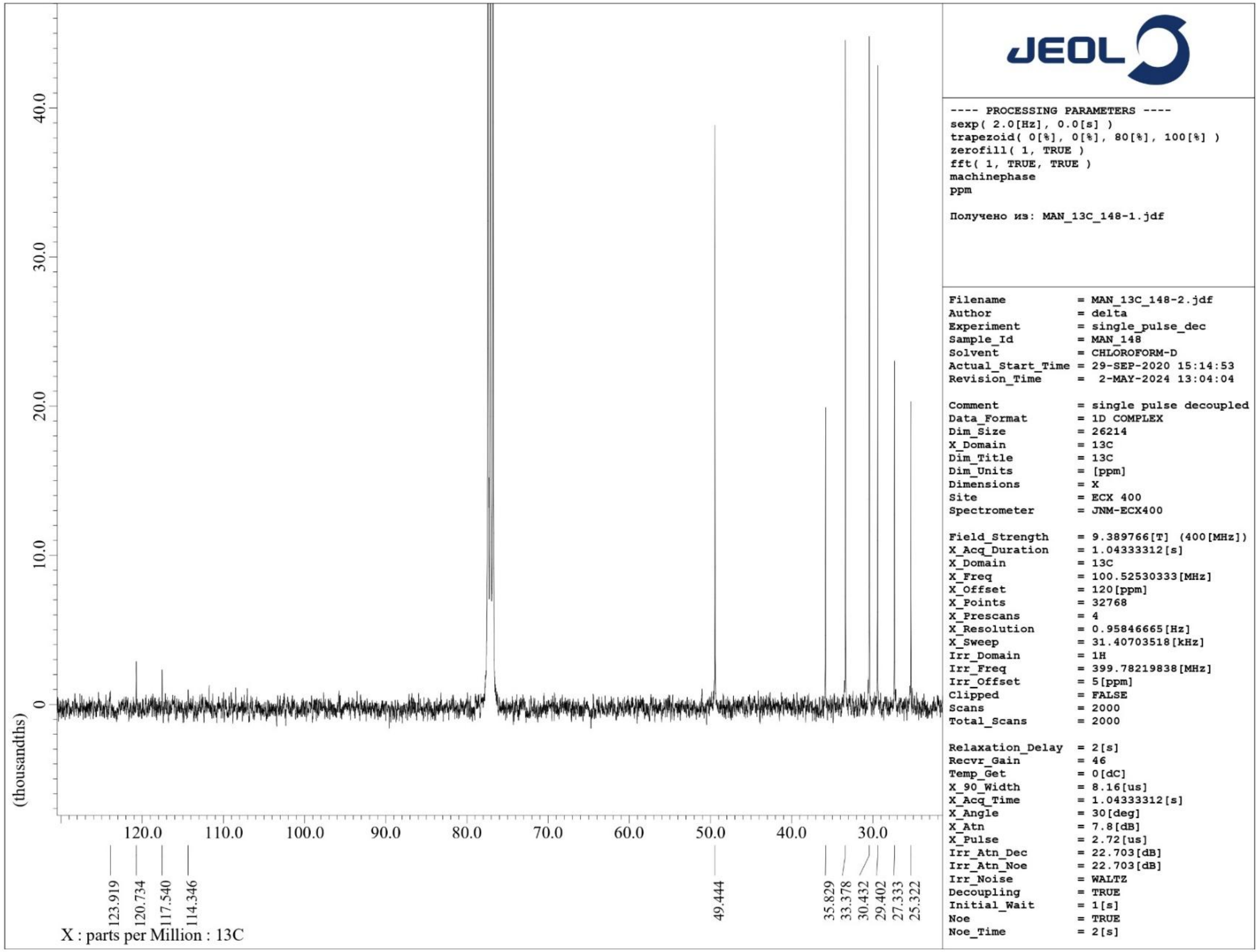

JEOL
---- PROCESSING PARAMETERS ----
sexp(2.0[Hz], 0.0[s])
trapezoid(0[%], 0[%], 80[%], 100[%])
zerofill(1, TRUE)
fft(1, TRUE, TRUE)
machinephase
ppm
Получено из: MAN_13C_148-1.jdf
Filename = MAN_13C_148-2.jdf
Author = delta
Experiment = single_pulse_dec
Sample_Id = MAN_148
Solvent = CHLOROFORM-D
Actual_Start_Time = 29-SEP-2020 15:14:53
Revision_Time = 2-MAY-2024 13:04:04
Comment = single pulse decoupled
Data_Format = 1D COMPLEX
Dim_Size = 26214
X_Domain = 13C
Dim_Title = 13C
Dim_Units = [ppm]
Dimensions = X
Site = ECX 400
Spectrometer = JNM-ECX400
Field_Strength = 9.389766[T] (400[MHz])
X_Acq_Duration = 1.04333312[s]
X_Domain = 13C
X_Freq = 100.52530333[MHz]
X_Offset = 120[ppm]
X_Points = 32768
X_Prescans = 4
X_Resolution = 0.95846665[Hz]
X_Sweep = 31.40703518[kHz]
Irr_Domain = 1H
Irr_Freq = 399.78219838[MHz]
Irr_Offset = 5[ppm]
Clipped = FALSE
Scans = 2000
Total_Scans = 2000
Relaxation_Delay = 2[s]
Recvr_Gain = 46
Temp_Get = 0[dC]
X_90_Width = 8.16[us]
X_Acq_Time = 1.04333312[s]
X_Angle = 30[deg]
X_Atn = 7.8[dB]
X_Pulse = 2.72[us]
Irr_Atn_Dec = 22.703[dB]
Irr_Atn_Noe = 22.703[dB]
Irr_Noise = WALTZ
Decoupling = TRUE
Initial_Wait = 1[s]
Noe = TRUE
Noe_Time = 2[s]
40.0
30.0
20.0
10.0
0
(thousandths)
120.0
110.0
100.0
90.0
80.0
70.0
60.0
50.0
40.0
30.0
123.919
120.734
117.540
114.346
49.444
35.829
33.378
30.432
29.402
27.333
25.322
X : parts per Million : 13C

3.11. NMR spectra of *trans*/*cis*-*N*-(5-azidotricyclo[4.3.1.1^{3,8}]undecan-4-yl)-4-methylbenzenesulfonamide (**13a,b**)

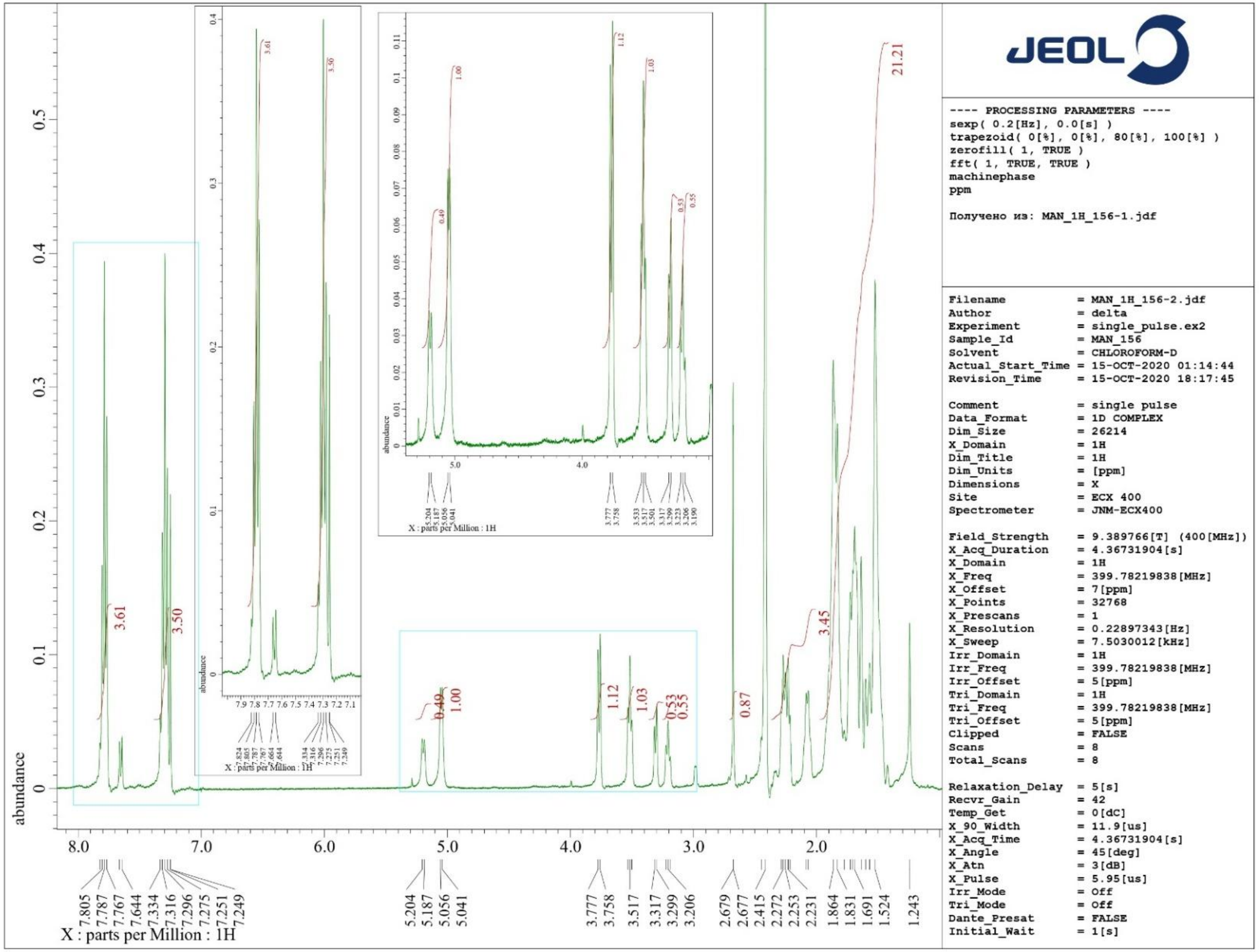

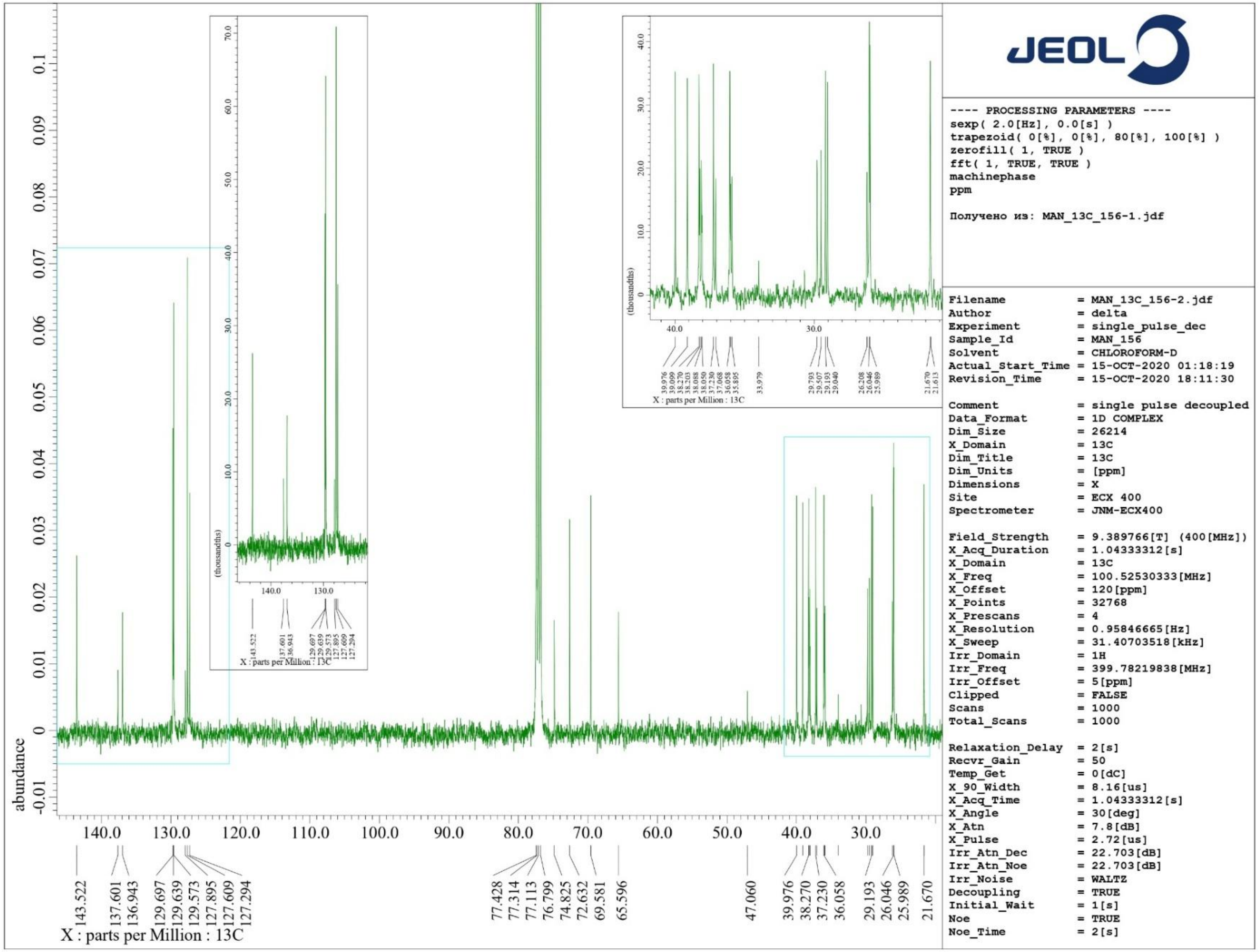
JEOL
---- PROCESSING PARAMETERS ----
sexp(2.0[Hz], 0.0[s])
trapezoid(0[%], 0[%], 80[%], 100[%])
zerofill(1, TRUE)
fft(1, TRUE, TRUE)
machinephase
ppm
Получено из: MAN_13C_156-1.jdf
Filename = MAN_13C_156-2.jdf
Author = delta
Experiment = single_pulse_dec
Sample_Id = MAN_156
Solvent = CHLOROFORM-D
Actual_Start_Time = 15-OCT-2020 01:18:19
Revision_Time = 15-OCT-2020 18:11:30
Comment = single pulse decoupled
Data_Format = 1D COMPLEX
Dim_Size = 26214
X_Domain = 13C
Dim_Title = 13C
Dim_Units = [ppm]
Dimensions = X
Site = ECX 400
Spectrometer = JNM-ECX400
Field_Strength = 9.389766[T] (400[MHz])
X_Acq_Duration = 1.04333312[s]
X_Domain = 13C
X_Freq = 100.52530333[MHz]
X_Offset = 120[ppm]
X_Points = 32768
X_Prescans = 4
X_Resolution = 0.95846665[Hz]
X_Sweep = 31.40703518[kHz]
Irr_Domain = 1H
Irr_Freq = 399.78219838[MHz]
Irr_Offset = 5[ppm]
Clipped = FALSE
Scans = 1000
Total_Scans = 1000
Relaxation_Delay = 2[s]
Recvr_Gain = 50
Temp_Get = 0[dC]
X_90_Width = 8.16[us]
X_Acq_Time = 1.04333312[s]
X_Angle = 30[deg]
X_Atn = 7.8[dB]
X_Pulse = 2.72[us]
Irr_Atn_Dec = 22.703[dB]
Irr_Atn_Noe = 22.703[dB]
Irr_Noise = WALTZ
Decoupling = TRUE
Initial_Wait = 1[s]
Noe = TRUE
Noe_Time = 2[s]
abundance
143.522
137.601
136.943
129.697
129.639
129.573
127.895
127.609
127.294
77.428
77.314
77.113
76.799
74.825
72.632
69.581
65.596
47.060
39.976
38.270
37.230
36.058
29.193
26.046
25.989
21.670
X : parts per Million : 13C

3.12. NMR spectra of *trans*-*N*-(5-azidotricyclo[4.3.1.1^{3,8}]undecan-4-yl)-1,1,1-trifluoromethanesulfonamide (**14**)

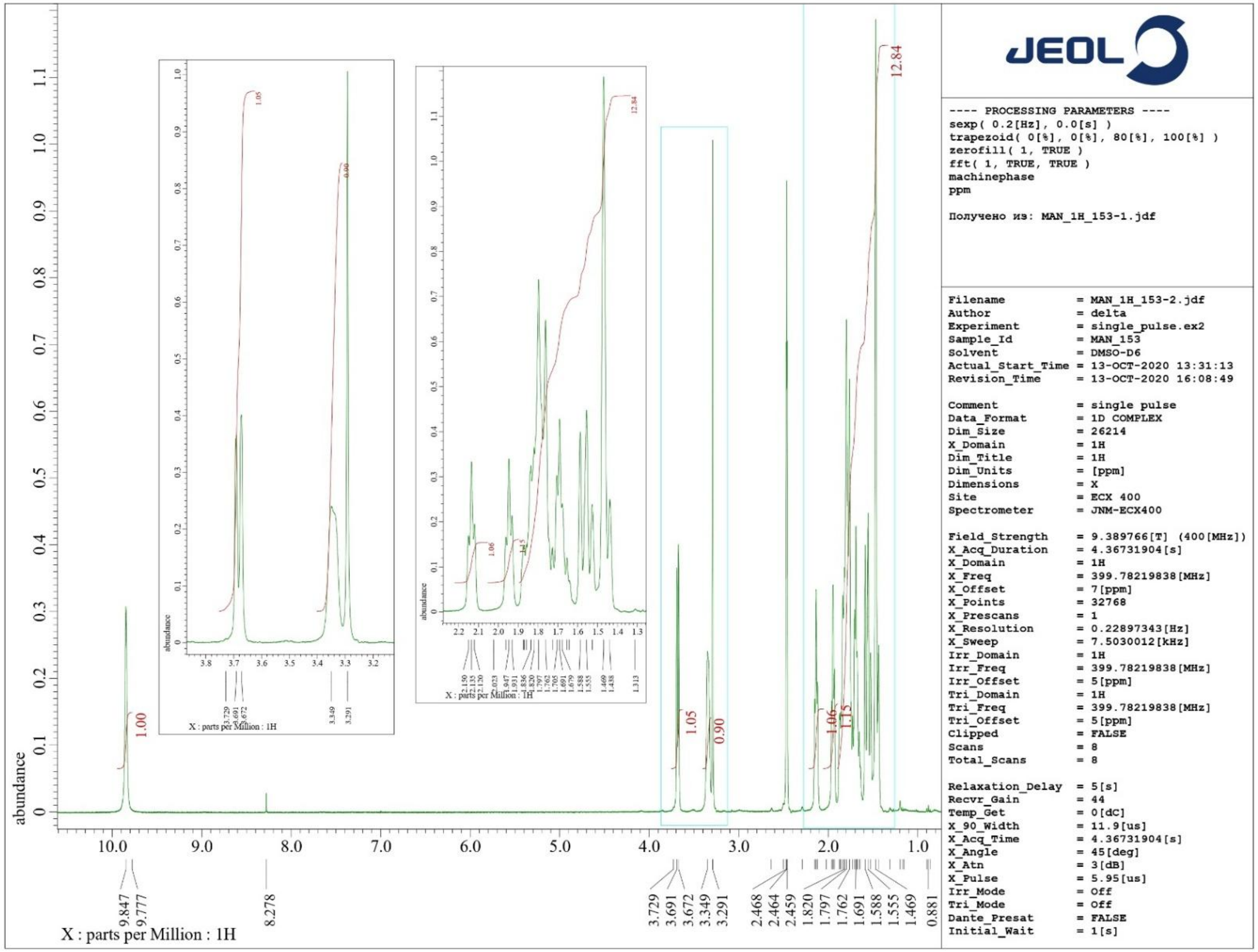

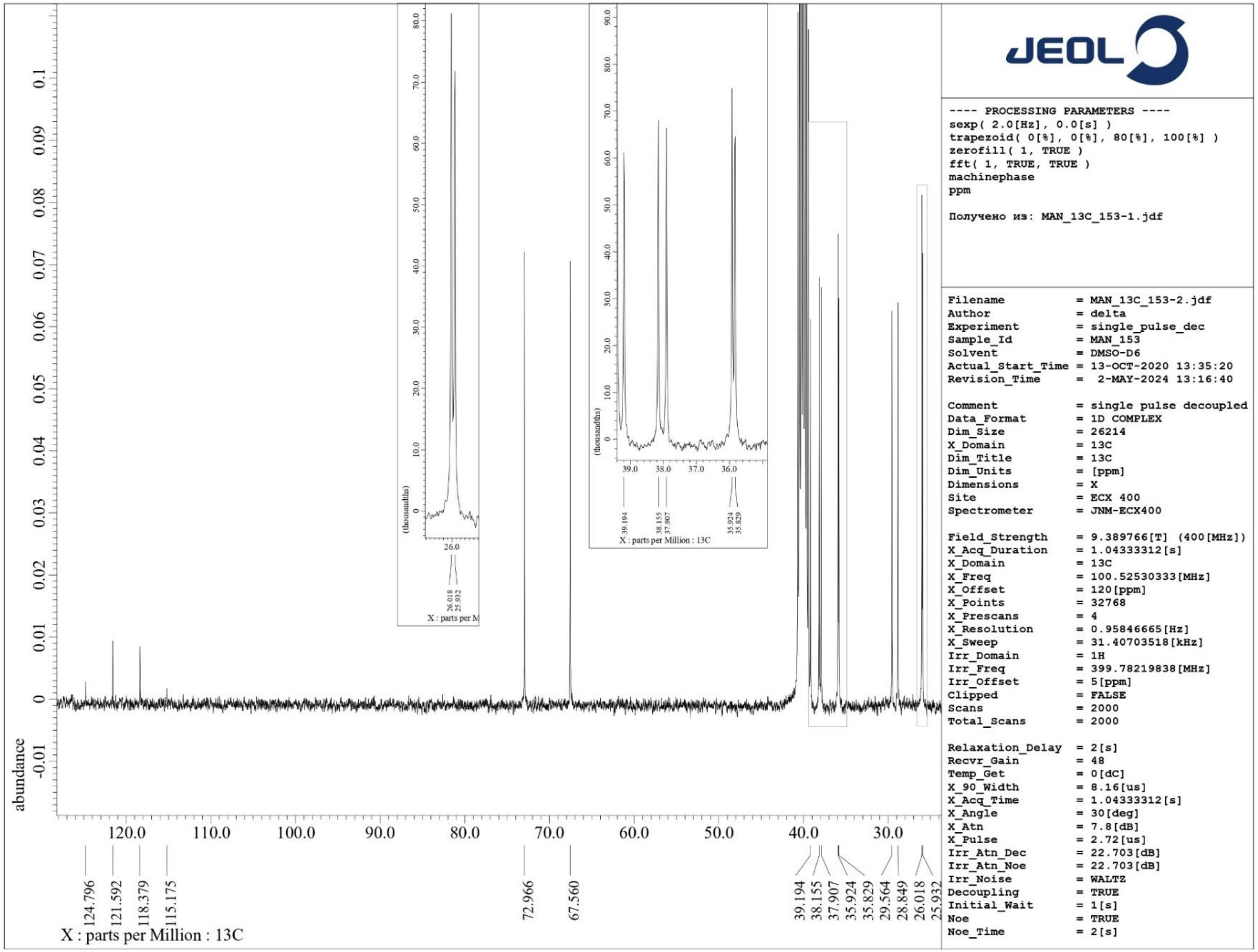
JEOL
---- PROCESSING PARAMETERS ----
sexp(2.0[Hz], 0.0[s])
trapezoid(0[%], 0[%], 80[%], 100[%])
zerofill(1, TRUE)
fft(1, TRUE, TRUE)
machinephase
ppm
Получено из: MAN_13C_153-1.jdf
Filename = MAN_13C_153-2.jdf
Author = delta
Experiment = single_pulse_dec
Sample_Id = MAN_153
Solvent = DMSO-D6
Actual_Start_Time = 13-OCT-2020 13:35:20
Revision_Time = 2-MAY-2024 13:16:40
Comment = single pulse decoupled
Data_Format = 1D COMPLEX
Dim_Size = 26214
X_Domain = 13C
Dim_Title = 13C
Dim_Units = [ppm]
Dimensions = X
Site = ECX 400
Spectrometer = JNM-ECX400
Field_Strength = 9.389766[T] (400[MHz])
X_Acq_Duration = 1.04333312[s]
X_Domain = 13C
X_Freq = 100.52530333[MHz]
X_Offset = 120[ppm]
X_Points = 32768
X_Prescans = 4
X_Resolution = 0.95846665[Hz]
X_Sweep = 31.40703518[kHz]
Irr_Domain = 1H
Irr_Freq = 399.78219838[MHz]
Irr_Offset = 5[ppm]
Clipped = FALSE
Scans = 2000
Total_Scans = 2000
Relaxation_Delay = 2[s]
Recvr_Gain = 48
Temp_Get = 0[dC]
X_90_Width = 8.16[us]
X_Acq_Time = 1.04333312[s]
X_Angle = 30[deg]
X_Atn = 7.8[dB]
X_Pulse = 2.72[us]
Irr_Atn_Dec = 22.703[dB]
Irr_Atn_Noe = 22.703[dB]
Irr_Noise = WALTZ
Decoupling = TRUE
Initial_Wait = 1[s]
Noe = TRUE
Noe_Time = 2[s]
abundance
X : parts per Million : 13C
124.796
121.592
118.379
115.175
72.966
67.560
39.194
38.155
37.907
35.924
35.829
29.564
28.849
26.018
25.932

3.13. NMR spectra of *trans*-*N*-(5-aminotricyclo[4.3.1.1^{3,8}]undecan-4-yl)-1,1,1-trifluoromethanesulfonamide (**15**)

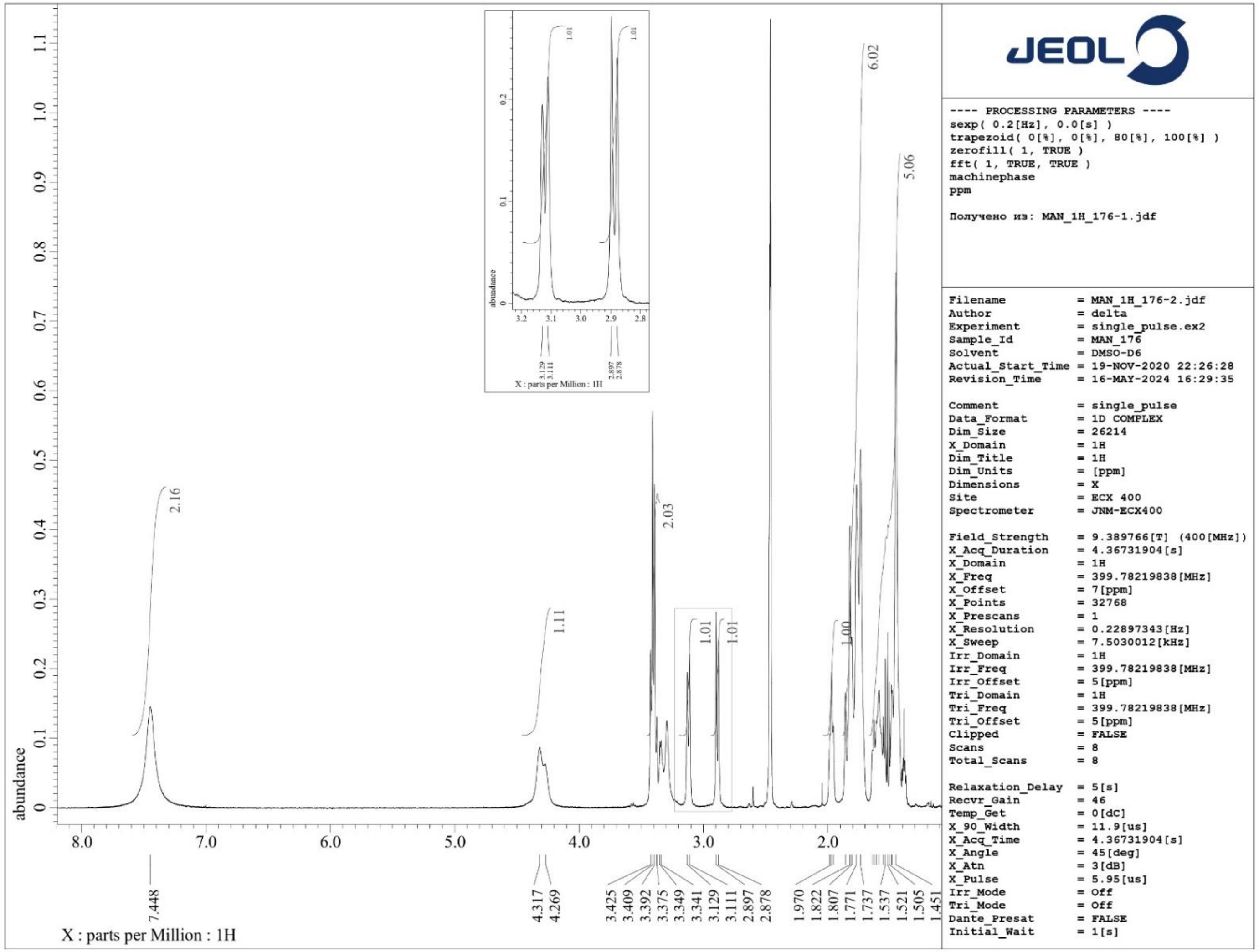

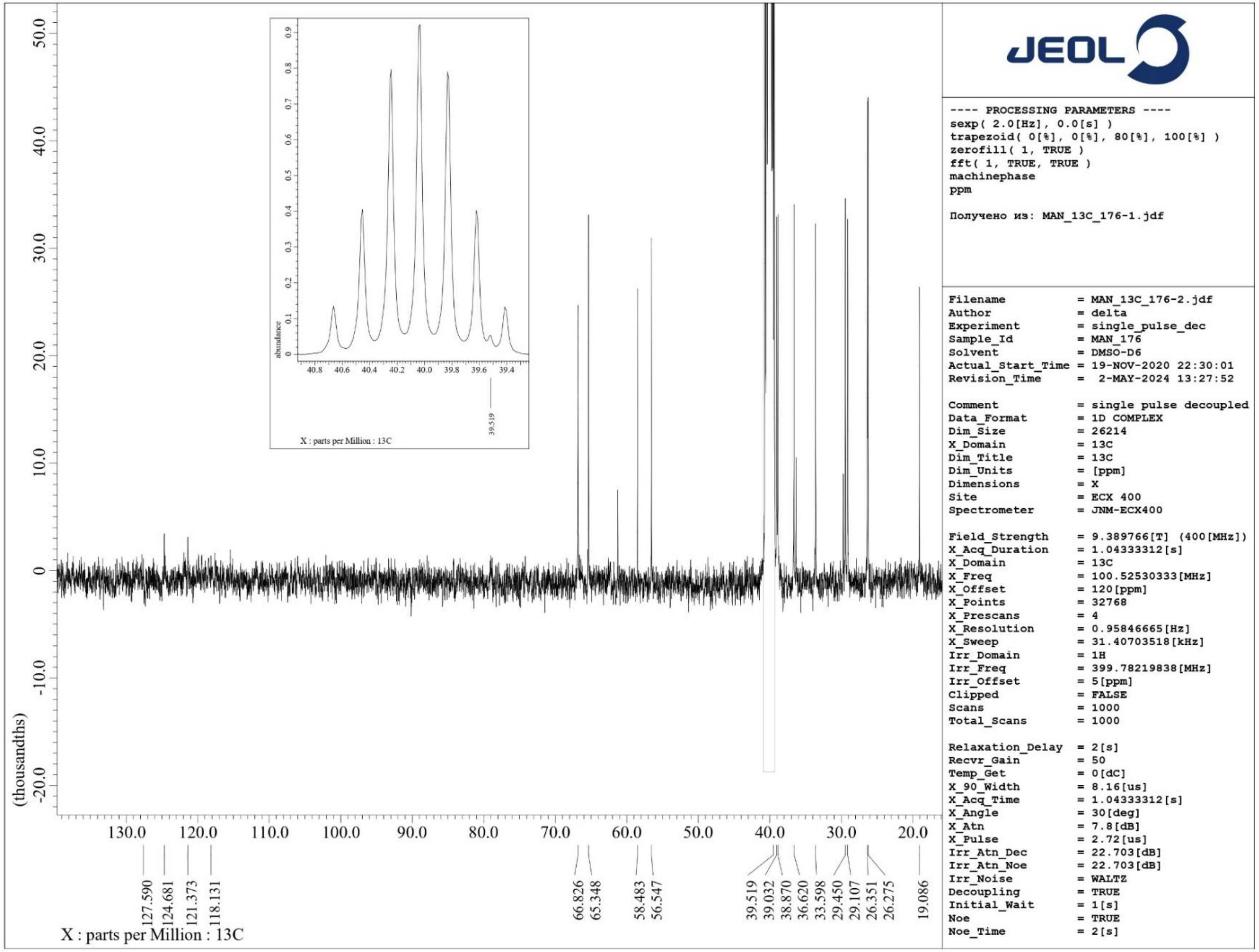

50.0
40.0
30.0
20.0
10.0
0
-10.0
-20.0
(thousandths)
130.0 120.0 110.0 100.0 90.0 80.0 70.0 60.0 50.0 40.0 30.0 20.0
127.590
124.681
121.373
118.131
66.826
65.348
58.483
56.547
39.519
39.032
38.870
36.620
33.598
29.450
29.107
26.351
26.275
19.086
X : parts per Million : 13C
abundance
40.8 40.6 40.4 40.2 40.0 39.8 39.6 39.4
X : parts per Million : 13C
---- PROCESSING PARAMETERS ----
sexp(2.0[Hz], 0.0[s])
trapezoid(0[%], 0[%], 80[%], 100[%])
zerofill(1, TRUE)
fft(1, TRUE, TRUE)
machinephase
ppm
Получено из: MAN_13C_176-1.jdf
Filename = MAN_13C_176-2.jdf
Author = delta
Experiment = single_pulse_dec
Sample_Id = MAN_176
Solvent = DMSO-D6
Actual_Start_Time = 19-NOV-2020 22:30:01
Revision_Time = 2-MAY-2024 13:27:52
Comment = single pulse decoupled
Data_Format = 1D COMPLEX
Dim_Size = 26214
X_Domain = 13C
Dim_Title = 13C
Dim_Units = [ppm]
Dimensions = X
Site = ECX 400
Spectrometer = JNM-ECX400
Field_Strength = 9.389766[T] (400[MHz])
X_Acq_Duration = 1.04333312[s]
X_Domain = 13C
X_Freq = 100.52530333[MHz]
X_Offset = 120[ppm]
X_Points = 32768
X_Prescans = 4
X_Resolution = 0.95846665[Hz]
X_Sweep = 31.40703518[kHz]
Irr_Domain = 1H
Irr_Freq = 399.78219838[MHz]
Irr_Offset = 5[ppm]
Clipped = FALSE
Scans = 1000
Total_Scans = 1000
Relaxation_Delay = 2[s]
Recvr_Gain = 50
Temp_Get = 0[dC]
X_90_Width = 8.16[us]
X_Acq_Time = 1.04333312[s]
X_Angle = 30[deg]
X_Atn = 7.8[dB]
X_Pulse = 2.72[us]
Irr_Atn_Dec = 22.703[dB]
Irr_Atn_Noe = 22.703[dB]
Irr_Noise = WALTZ
Decoupling = TRUE
Initial_Wait = 1[s]
Noe = TRUE
Noe_Time = 2[s]

3.14. NMR spectra of *trans*-decahydro-4,8:6,10-dimethanocyclonona[*d*]imidazole-2(1*H*)-thione (**16**)

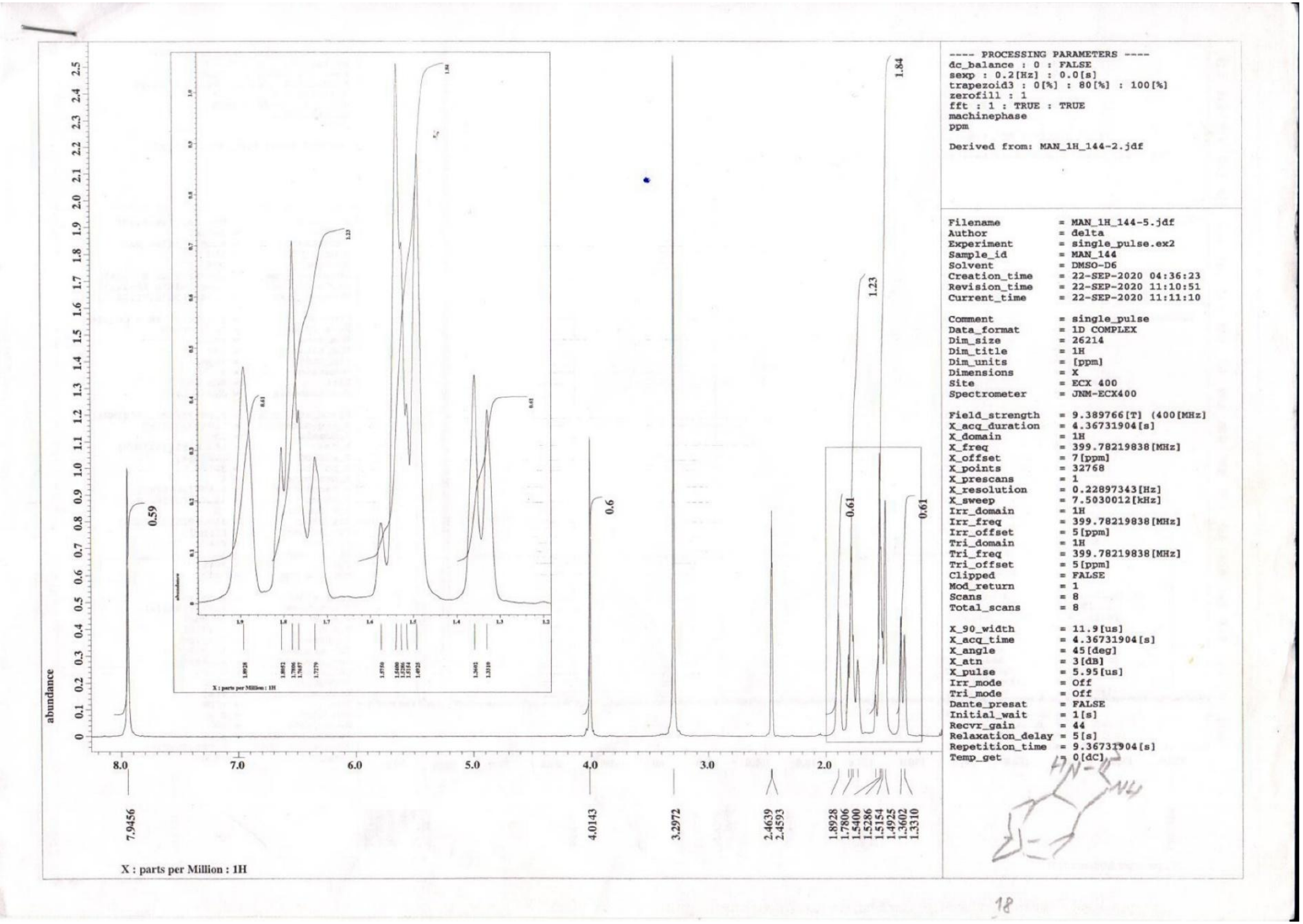

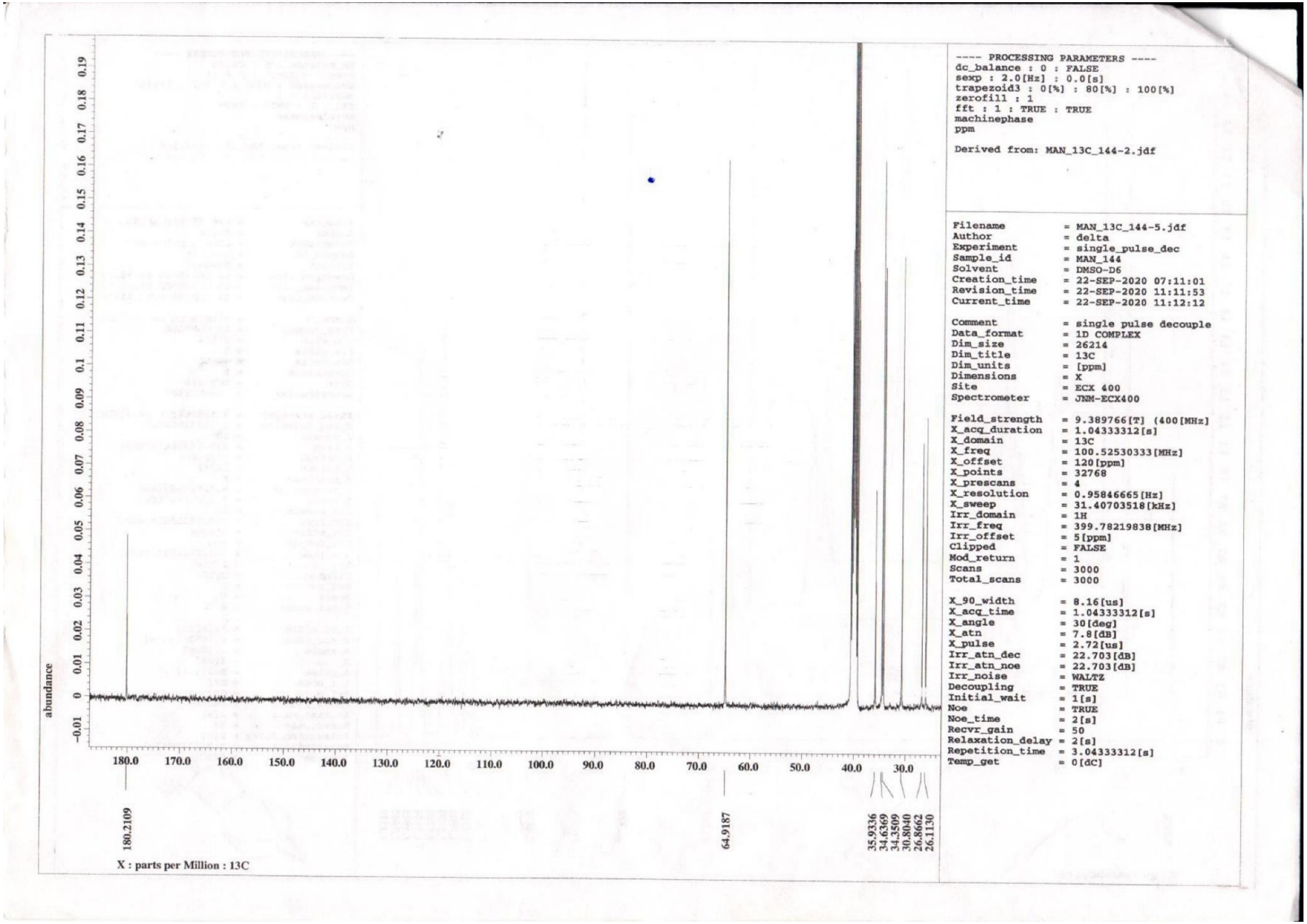
---- PROCESSING PARAMETERS ----
dc_balance : 0 : FALSE
sexp : 2.0[Hz] : 0.0[s]
trapezoid3 : 0[%] : 80[%] : 100[%]
zerofill : 1
fft : 1 : TRUE : TRUE
machinephase
ppm
Derived from: MAN_13C_144-2.jdf
Filename = MAN_13C_144-5.jdf
Author = delta
Experiment = single_pulse_dec
Sample_id = MAN_144
Solvent = DMSO-D6
Creation_time = 22-SEP-2020 07:11:01
Revision_time = 22-SEP-2020 11:11:53
Current_time = 22-SEP-2020 11:12:12
Comment = single pulse decouple
Data_format = 1D COMPLEX
Dim_size = 26214
Dim_title = 13C
Dim_units = [ppm]
Dimensions = X
Site = ECX 400
Spectrometer = JNM-ECX400
Field_strength = 9.389766[T] (400[MHz]
X_acq_duration = 1.04333312[s]
X_domain = 13C
X_freq = 100.52530333[MHz]
X_offset = 120[ppm]
X_points = 32768
X_prescans = 4
X_resolution = 0.95846665[Hz]
X_sweep = 31.40703518[kHz]
Irr_domain = 1H
Irr_freq = 399.78219838[MHz]
Irr_offset = 5[ppm]
Clipped = FALSE
Mod_return = 1
Scans = 3000
Total_scans = 3000
X_90_width = 8.16[us]
X_acq_time = 1.04333312[s]
X_angle = 30[deg]
X_atn = 7.8[dB]
X_pulse = 2.72[us]
Irr_atn_dec = 22.703[dB]
Irr_atn_noe = 22.703[dB]
Irr_noise = WALTZ
Decoupling = TRUE
Initial_wait = 1[s]
Noe = TRUE
Noe_time = 2[s]
Recvr_gain = 50
Relaxation_delay = 2[s]
Repetition_time = 3.04333312[s]
Temp_get = 0[dC]
abundance
X : parts per Million : 13C
180.2109
64.9187
35.9336
34.6369
34.3509
30.8040
26.8662
26.1130

3.15. NMR spectra of *rac*-2,3-diphenyl-4*a*,6,7,8,9,10,11,11*a*-octahydro-5*H*-5,9:7,11-dimethanocyclonona[*b*]pyrazine (**17**)

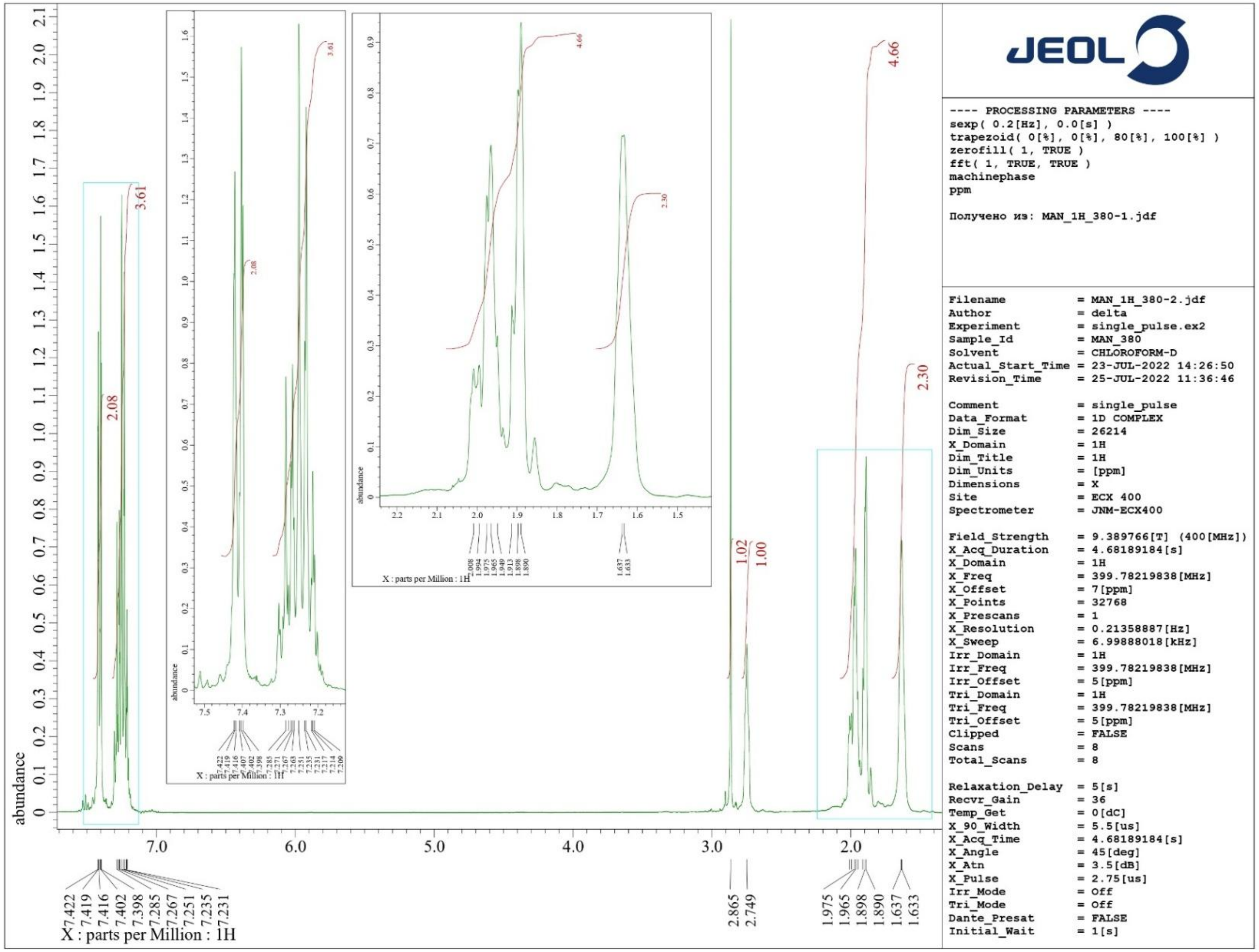

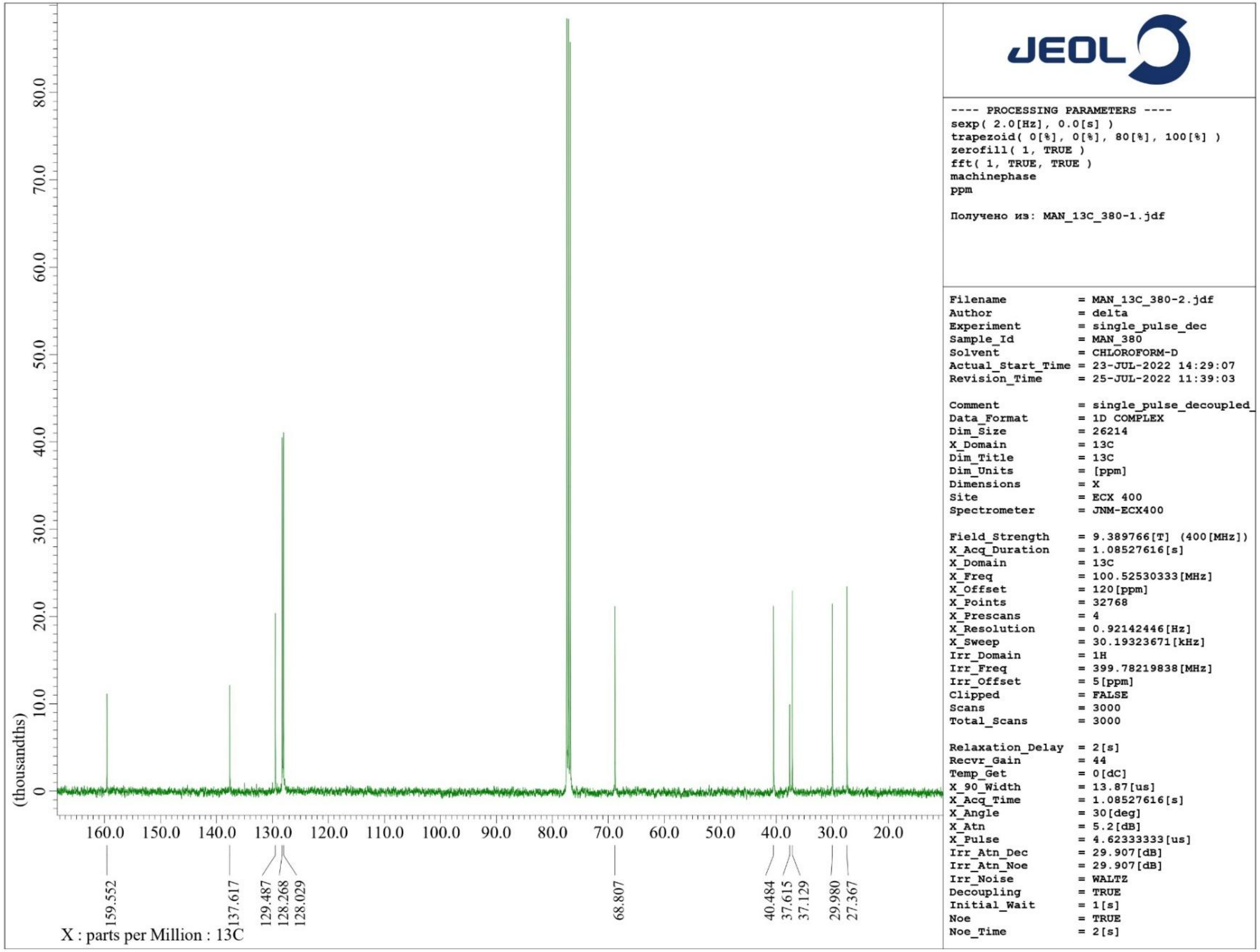


```
---- PROCESSING PARAMETERS ----
sexp( 2.0[Hz], 0.0[s] )
trapezoid( 0[%], 0[%], 80[%], 100[%] )
zerofill( 1, TRUE )
fft( 1, TRUE, TRUE )
machinephase
ppm

Получено из: MAN_13C_380-1.jdf

Filename           = MAN_13C_380-2.jdf
Author             = delta
Experiment         = single_pulse_dec
Sample_Id          = MAN_380
Solvent            = CHLOROFORM-D
Actual_Start_Time  = 23-JUL-2022 14:29:07
Revision_Time      = 25-JUL-2022 11:39:03

Comment            = single_pulse_decoupled_
Data_Format        = 1D COMPLEX
Dim_Size           = 26214
X_Domain           = 13C
Dim_Title          = 13C
Dim_Units          = [ppm]
Dimensions         = X
Site               = ECX 400
Spectrometer       = JNM-ECX400

Field_Strength     = 9.389766[T] (400[MHz])
X_Acq_Duration     = 1.08527616[s]
X_Domain           = 13C
X_Freq             = 100.52530333[MHz]
X_Offset           = 120[ppm]
X_Points           = 32768
X_Prescans         = 4
X_Resolution       = 0.92142446[Hz]
X_Sweep            = 30.19323671[kHz]
Irr_Domain         = 1H
Irr_Freq           = 399.78219838[MHz]
Irr_Offset         = 5[ppm]
Clipped            = FALSE
Scans              = 3000
Total_Scans        = 3000

Relaxation_Delay   = 2[s]
Recvr_Gain         = 44
Temp_Get           = 0[dC]
X_90_Width         = 13.87[us]
X_Acq_Time         = 1.08527616[s]
X_Angle            = 30[deg]
X_Atn              = 5.2[dB]
X_Pulse            = 4.62333333[us]
Irr_Atn_Dec        = 29.907[dB]
Irr_Atn_Noe        = 29.907[dB]
Irr_Noise          = WALTZ
Decoupling         = TRUE
Initial_Wait       = 1[s]
Noe                = TRUE
Noe_Time           = 2[s]
```

3.16. NMR spectra of (4*R*,5*R*)-*N*,*N*´-(tricyclo[4.3.1.1^{3,8}]undecane-4,5-diyl)bis(1-phenylmethanimine) (**18**)

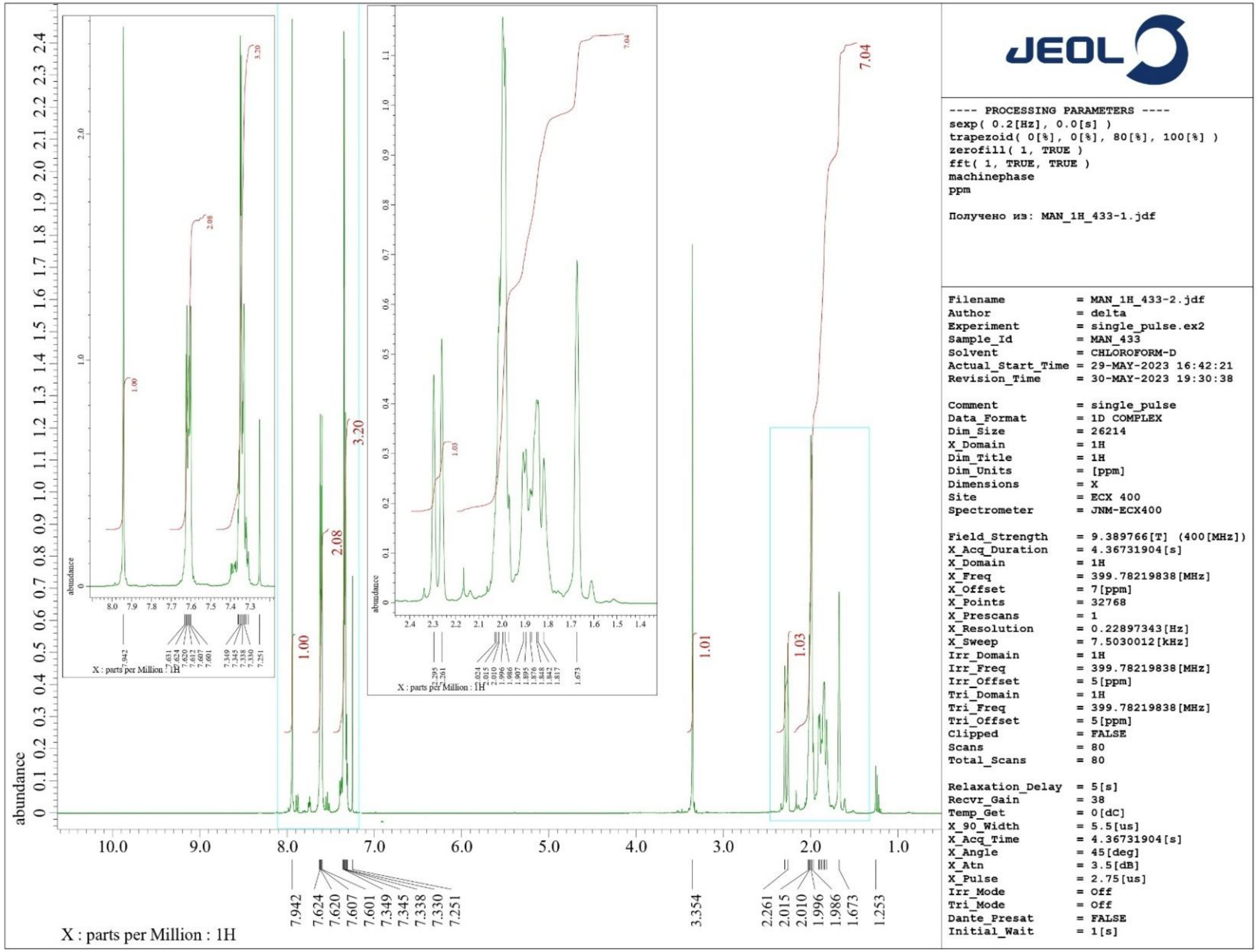

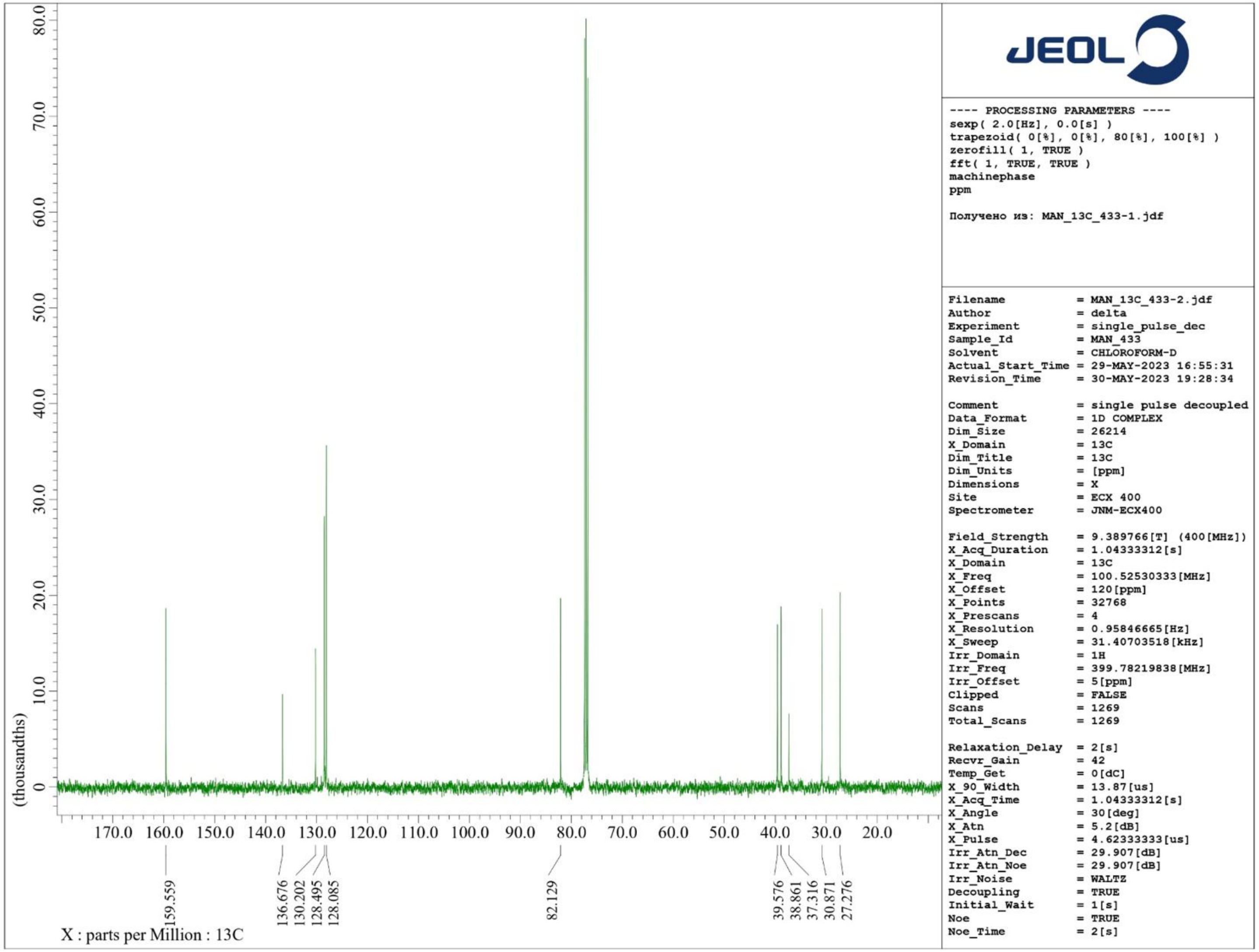
JEOL
---- PROCESSING PARAMETERS ----
sexp(2.0[Hz], 0.0[s])
trapezoid(0[%], 0[%], 80[%], 100[%])
zerofill(1, TRUE)
fft(1, TRUE, TRUE)
machinephase
ppm
Получено из: MAN_13C_433-1.jdf
Filename = MAN_13C_433-2.jdf
Author = delta
Experiment = single_pulse_dec
Sample_Id = MAN_433
Solvent = CHLOROFORM-D
Actual_Start_Time = 29-MAY-2023 16:55:31
Revision_Time = 30-MAY-2023 19:28:34
Comment = single pulse decoupled
Data_Format = 1D COMPLEX
Dim_Size = 26214
X_Domain = 13C
Dim_Title = 13C
Dim_Units = [ppm]
Dimensions = X
Site = ECX 400
Spectrometer = JNM-ECX400
Field_Strength = 9.389766[T] (400[MHz])
X_Acq_Duration = 1.04333312[s]
X_Domain = 13C
X_Freq = 100.52530333[MHz]
X_Offset = 120[ppm]
X_Points = 32768
X_Prescans = 4
X_Resolution = 0.95846665[Hz]
X_Sweep = 31.40703518[kHz]
Irr_Domain = 1H
Irr_Freq = 399.78219838[MHz]
Irr_Offset = 5[ppm]
Clipped = FALSE
Scans = 1269
Total_Scans = 1269
Relaxation_Delay = 2[s]
Recvr_Gain = 42
Temp_Get = 0[dC]
X_90_Width = 13.87[us]
X_Acq_Time = 1.04333312[s]
X_Angle = 30[deg]
X_Atn = 5.2[dB]
X_Pulse = 4.62333333[us]
Irr_Atn_Dec = 29.907[dB]
Irr_Atn_Noe = 29.907[dB]
Irr_Noise = WALTZ
Decoupling = TRUE
Initial_Wait = 1[s]
Noe = TRUE
Noe_Time = 2[s]
(thousandths)
0
10.0
20.0
30.0
40.0
50.0
60.0
70.0
80.0
170.0 160.0 150.0 140.0 130.0 120.0 110.0 100.0 90.0 80.0 70.0 60.0 50.0 40.0 30.0 20.0
159.559
136.676
130.202
128.495
128.085
82.129
39.576
38.861
37.316
30.871
27.276
X : parts per Million : 13C

4. Computational details of specific rotation determination of (4*R*,5*R*)-8a´

The crude geometry of initial diamine was built using Avogadro 1.2.1 [6] software package and proceeded for further optimization in gaussian 09.a [7]. Optimization of geometry was performed by HF/6-31G+(d) method. The initial dihedral angles of C5–C4–N–H(*si*) were set to 170.849, 48.603 and −60.801, keeping dihedral angle C4–C5–N–H(*si*) at approximately 171.5 degrees with further optimization of geometry of received 3 conformers along the C4–N bond. In each of the resulting structures the bond C5–N was scanned in 15 degrees using C4–C5–N–H(*si*) as redundant coordinates. All in 72 conformations were obtained and ranged by their relative energy with respect to the most stable conformer 1_9. The results of geometry scans are presented in Tables S1–S3. From all structures those with the relative energy not more than 10 kJ/mol were chosen for further optimization as fraction in the solution would be the highest among all other conformers. Further geometry optimization of selected molecules with frozen C4–C5–N–H(*si*) dihedrals was performed by CAM-B3LYP/6-311G++(2d,2p) method with consideration of thermochemistry at 298 K and solvation with CH_2Cl_2 in SMD model. Calculation of specific rotation of optimized structures was performed on the same level of theory for 365, 405, 436, 546, 589.3 and 633 nm. The fraction of each conformer was calculated from Boltzman law, using relative energy of each conformer. Total specific rotation at specific wavelength is calculated as a sum through all conformers of products of specific rotation of each conformer at selected wavelength on its fraction:

$$\left[\alpha_{\lambda}^{20}(total)\right] = \sum_{conformers} x(conf) * [\alpha_{\lambda}^{20}(conf)]$$

The results of specific rotation calculation are presented in table 4.

Table S1: Data for C5–NH_2 bond scan with C4–NH_2 conformation with 50.665 degree dihedral angle (C5–C4–N–H(*re*) = −69.519 deg, C5–C4–N–H(*si*) = 170.849 deg)

Conformer name	Dihedral angle C4–C5–N–H(*re*)	Dihedral angle C4–C5–N–H(*si*)	Dihedral angle C4–C5–N-lone pair	Energy HF/6-31G(d), Hartree	Relative energy, kJ/mol
1_1	50.288	-67.320	171.484	-537.096805854	5.87
1_2	63.257	-52.320	-174.532	-537.096182651	7.51
1_3	77.657	-37.321	-159.832	-537.094637178	11.56
1_4	93.842	-22.321	-144.239	-537.093118355	15.55
1_5	111.374	-7.321	-127.973	-537.092763445	16.48
1_6	128.506	7.678	-111.908	-537.094041041	13.13
1_7	143.522	22.678	-96.899	-537.096324272	7.13
1_8	156.879	37.678	-82.720	-537.098384732	1.72
1_9	169.862	52.678	-68.729	-537.099041282	0.00
1_10	-176.658	67.678	-54.489	-537.097770719	3.34
1_11	-161.951	82.678	-39.636	-537.095050626	10.48
1_12	-145.569	97.678	-23.945	-537.092120768	18.17
1_13	-128.001	112.678	-7.661	-537.090265877	23.04
1_14	-110.950	127.678	8.364	-537.089931489	23.92
1_15	-95.513	142.678	23.582	-537.090601312	22.16
1_16	-81.395	157.678	38.141	-537.091394945	20.08

1_17	-67.693	172.678	52.492	-537.091570833	19.61
1_18	-53.560	-172.321	67.059	-537.090802794	21.63
1_19	-37.486	-157.321	82.595	-537.089439123	25.21
1_20	-17.094	-142.321	100.292	-537.088911610	26.60
1_21	1.866	-127.321	117.272	-537.090181142	23.26
1_22	15.439	-112.321	131.559	-537.092296619	17.71
1_23	26.976	-97.321	144.827	-537.094499192	11.93
1_24	38.351	-82.321	158.0154	-537.096173643	7.53

Table S2: Data for C5–NH_2 bond scan with C4–NH_2 conformation with −61.163 degree dihedral angle (C5–C4–N–H(*re*) = 167.440 deg, C5–C4–N–H(*si*) = 48.603 deg)

Conformer name	Dihedral angle C4–C5–N–H(*re*)	Dihedral angle C4–C5–N–H(*si*)	Dihedral angle C4–C5–N-lone pair	Energy HF/6-31G(d), Hartree	Relative energy, kJ/mol
2_1	58.454	-60.981	178.737	-537.094624661	11.60
2_2	73.800	-45.981	-166.090	-537.094276858	12.51
2_3	90.297	-30.981	-150.342	-537.093586245	14.32
2_4	107.319	-15.981	-134.331	-537.093167343	15.42
2_5	123.958	-0.981	-118.512	-537.093475631	14.61
2_6	138.625	14.019	-103.678	-537.094484453	11.96
2_7	151.419	29.019	-89.781	-537.095674893	8.84

2_8	163.588	44.019	-76.197	-537.096358162	7.04
2_9	176.137	59.019	-62.422	-537.096035533	7.89
2_10	-170.096	74.020	-48.038	-537.094740120	11.29
2_11	-154.222	89.019	-32.601	-537.093271971	15.15
2_12	-136.213	104.019	-16.097	-537.092779570	16.44
2_13	-117.920	119.019	0.550	-537.093763168	13.86
2_14	-102.121	134.019	15.949	-537.095785137	8.55
2_15	-88.544	149.019	30.238	-537.097832087	3.17
2_16	-75.762	164.019	44.128	-537.098988203	0.14
2_17	-62.822	179.019	58.098	-537.098688533	0.93
2_18	-49.043	-165.981	72.488	-537.096941873	5.51
2_19	-33.767	-150.981	87.626	-537.094462651	12.02
2_20	-16.973	-135.981	103.523	-537.092419340	17.39
2_21	-0.022	-120.981	119.499	-537.091659008	19.38
2_22	15.702	-105.981	134.860	-537.092139089	18.12
2_23	30.094	-90.981	149.557	-537.093227500	15.26
2_24	44.138	-75.981	164.079	-537.094227250	12.64

Table S3: Data for C5–NH_2 bond scan with C4–NH_2 conformation with 178.953 degree dihedral angle (C5–C4–N–H(*re*) = 58.707 deg, C5–C4–N–H(*si*) = −60.801 deg)

Conformer name	Dihedral angle C4–C5–N–H(*re*)	Dihedral angle C4–C5–N–H(*si*)	Dihedral angle C4–C5–N-lone pair	Energy HF/6-31G(d), Hartree	Relative energy, kJ/mol
3_1	58.718	-60.788	178.965	-537.092853637	16.25
3_2	73.652	-45.788	-166.068	-537.092458951	17.28
3_3	89.905	-30.789	-150.442	-537.091640971	19.43
3_4	107.338	-15.788	-134.225	-537.091130661	20.77
3_5	124.447	-0.788	-118.170	-537.091462134	19.90
3_6	139.160	14.211	-103.314	-537.092545685	17.05
3_7	151.890	29.211	-89.449	-537.093810681	13.73
3_8	163.878	44.212	-75.955	-537.094576899	11.72
3_9	176.377	59.212	-62.206	-537.094346597	12.33
3_10	-169.705	74.212	-47.746	-537.093140073	15.49
3_11	-153.880	89.211	-32.334	-537.091635391	19.44
3_12	-136.093	104.211	-15.941	-537.090861769	21.48
3_13	-117.933	119.212	0.639	-537.091582499	19.58
3_14	-102.005	134.212	16.104	-537.093467608	14.63
3_15	-88.251	149.212	30.480	-537.095497146	9.31
3_16	-75.383	164.212	44.414	-537.096705412	6.13

3_17	-62.457	179.211	58.377	-537.096481597	6.72
3_18	-48.649	-165.789	72.781	-537.094802629	11.13
3_19	-33.379	-150.789	87.916	-537.092369669	17.52
3_20	-16.643	-135.789	103.784	-537.090351836	22.81
3_21	0.387	-120.788	119.799	-537.089610645	24.76
3_22	16.189	-105.788	135.201	-537.090144279	23.36
3_23	30.712	-90.789	149.962	-537.091330091	20.25
3_24	44.659	-75.788	164.435	-537.092422075	17.38

Table S4: Energies for most stable conformers, their distribution and specific rotation for 6 wavelengths

Conformer name	Energy DFT/CAM-B3LYP/6-311G++(2d.2p)/ SMD(CH_2Cl_2)., Hartree	Relative energy, kJ/mol	Conformer fraction, *x*(conf)	Specific rotation, $[\alpha_{\lambda}^{20}(conf)]$. wavelength					
				365 nm	405 nm	436 nm	546 nm	589.3 nm	633 nm
1_1	-540.605881	3.02	0.048	212.49	151.21	121.13	65.86	54.43	45.78
1_2	-540.605333	4.46	0.027	244.84	176.80	142.77	78.92	65.48	55.23
1_7	-540.605205	4.79	0.023	13.75	11.05	9.35	5.56	4.67	3.97
1_8	-540.606620	1.08	0.104	1.47	-1.45	-2.34	-2.72	-2.55	-2.34
1_9	-540.607030	0.00	0.161	24.52	12.59	7.86	1.61	0.80	0.31
1_10	-540.606068	2.53	0.058	71.40	45.11	33.53	15.10	11.86	9.55
1_24	-540.605484	4.06	0.031	179.17	124.50	98.41	51.97	42.66	35.68

2_7	-540.604932	5.51	0.017	92.97	62.30	48.22	24.30	19.72	16.35
2_8	-540.605541	3.91	0.033	93.73	60.09	45.19	21.18	16.85	13.73
2_9	-540.605386	4.32	0.028	120.96	78.64	59.66	28.55	22.83	18.70
2_14	-540.604045	7.84	0.007	112.97	78.02	61.40	32.06	26.23	21.88
2_15	-540.605744	3.38	0.041	59.48	39.51	30.35	14.93	12.02	9.90
2_16	-540.606864	0.44	0.135	22.94	12.39	8.11	2.23	1.41	0.89
2_17	-540.606947	0.22	0.147	21.55	10.44	6.13	0.65	0.00	-0.36
2_18	-540.605873	3.04	0.047	60.56	38.12	28.28	12.72	9.98	8.04
3_15	-540.604453	6.77	0.010	289.97	206.52	165.57	90.25	74.65	62.83
3_16	-540.605651	3.62	0.037	232.57	165.45	132.55	72.09	59.59	50.13
3_17	-540.605799	3.23	0.044	202.31	143.80	115.12	62.48	51.62	43.40

Coordinates of conformer 1_1

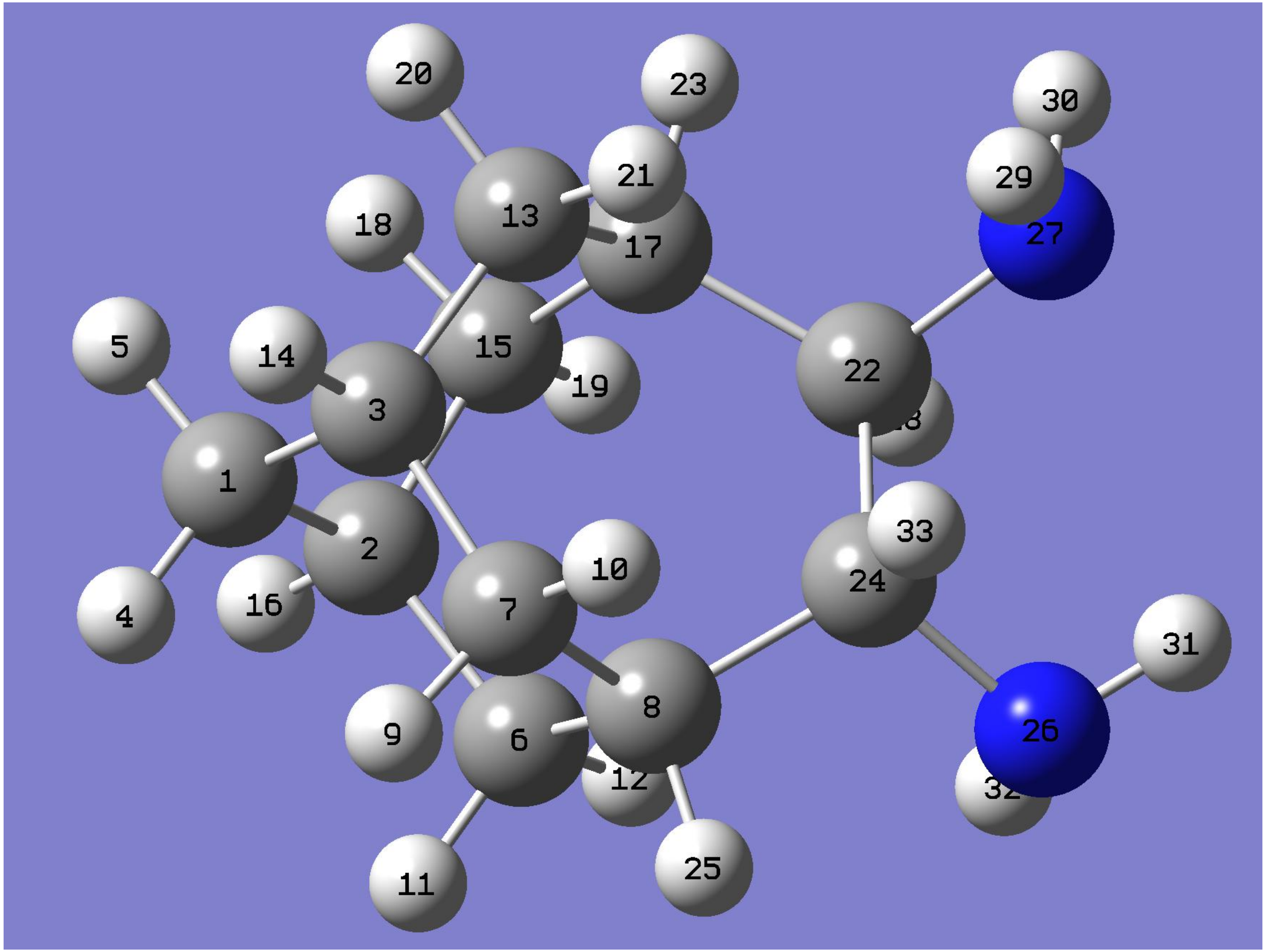
20
23
30
21
29
13
27
18
17
5
14
15
22
19
3
1
33
2
24
10
16
4
7
31
8
9
26
6
12
25
11

Tag	Symbol	X	Y	Z
1	C	-2.4685380	0.0183170	-0.0045110
2	C	-1.5793060	-0.2418790	-1.2208280
3	C	-1.5804710	0.2627990	1.2159250
4	H	-3.1244450	-0.8348470	0.1826940
5	H	-3.1085410	0.8828860	-0.1948410
6	C	-0.6927760	-1.4744980	-0.9818440
7	C	-0.8121670	-1.0239160	1.5069650
8	C	0.0853850	-1.4745820	0.3477050
9	H	-1.5413240	-1.8117170	1.7117570
10	H	-0.2091550	-0.9150630	2.4106710
11	H	-1.3411800	-2.3535940	-0.9856230
12	H	-0.0044320	-1.6041540	-1.8210070
13	C	-0.6733690	1.4812600	0.9813320
14	H	-2.2097050	0.4831680	2.0807140
15	C	-0.7881100	1.0314700	-1.5092620
16	H	-2.2082800	-0.4527360	-2.0881800
17	C	0.1093930	1.4651230	-0.3437310
18	H	-1.5015430	1.8315280	-1.7219930
19	H	-0.1800870	0.9086640	-2.4082800
20	H	-1.3072560	2.3709840	0.9843900
21	H	0.0161630	1.5985100	1.8216150
22	C	1.4332560	0.6757190	-0.3248770
23	H	0.4126250	2.4949830	-0.5468080
24	C	1.4211760	-0.7173820	0.3354890
25	H	0.3690750	-2.5081430	0.5498220
26	N	2.4752800	-1.5804030	-0.2212670
27	N	2.5521980	1.4165560	0.2752270
28	H	1.7052670	0.5235590	-1.3715220
29	H	2.3439830	1.6294450	1.2448310
30	H	2.6751070	2.3053150	-0.1949040
31	H	3.3448320	-1.0590670	-0.2380980
32	H	2.2602210	-1.7919790	-1.1901510
33	H	1.6894880	-0.5653910	1.3830190

Coordinates of conformer 1_2

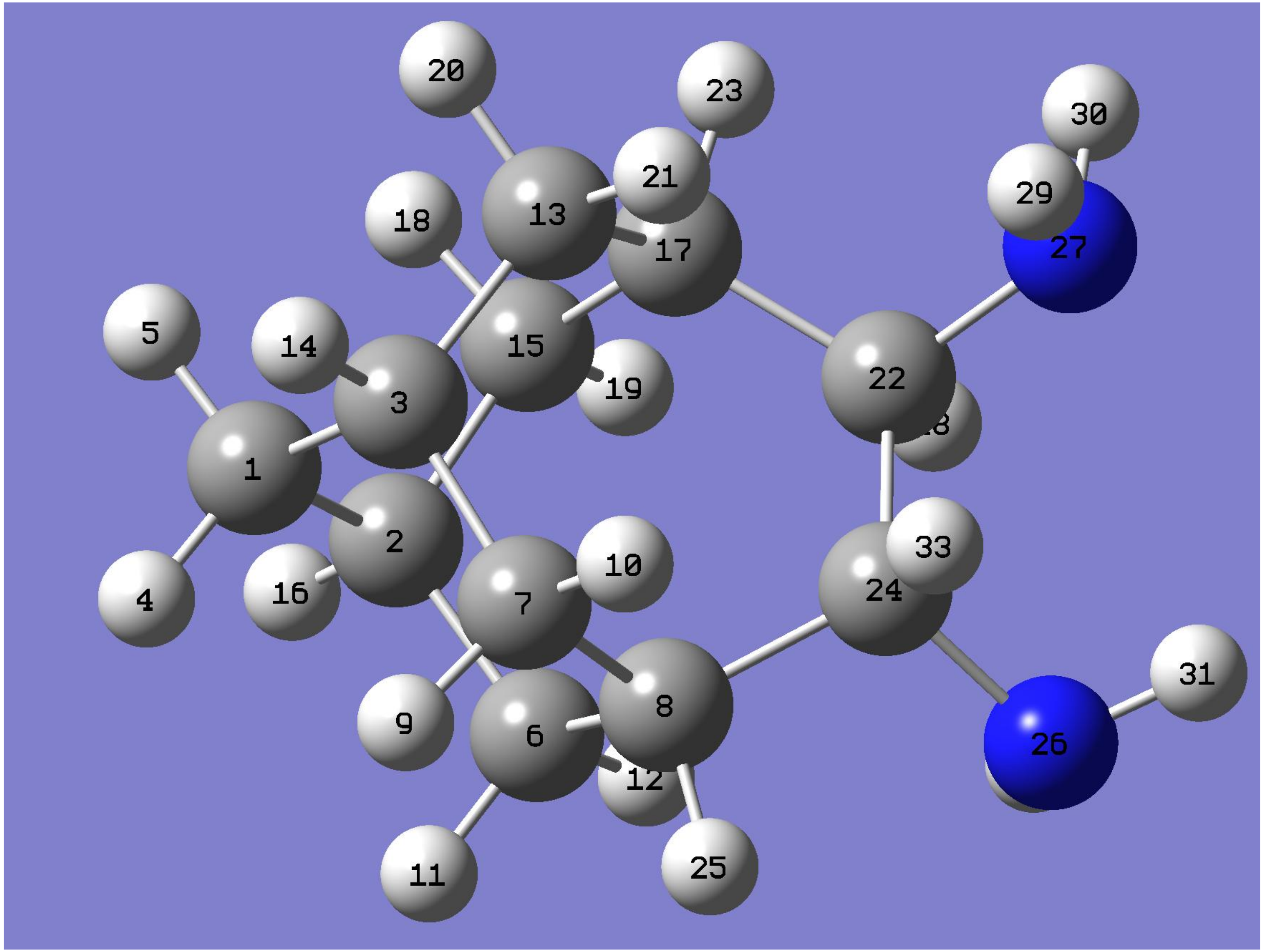

Tag	Symbol	X	Y	Z
1	C	2.4692240	0.0108600	0.0046570
2	C	1.5796240	-0.2807950	1.2136250
3	C	1.5822580	0.2925750	-1.2085840
4	H	3.1223920	-0.8389970	-0.2058850
5	H	3.1121010	0.8678610	0.2183190
6	C	0.6950850	-1.5071410	0.9412220
7	C	0.8025660	-0.9789830	-1.5333840
8	C	-0.0889230	-1.4655200	-0.3834340
9	H	1.5245450	-1.7644810	-1.7701060
10	H	0.1937020	-0.8363020	-2.4287200
11	H	1.3457530	-2.3842490	0.9144280
12	H	0.0103540	-1.6653740	1.7782770
13	C	0.6832680	1.5089240	-0.9412320
14	H	2.2126710	0.5311690	-2.0677100
15	C	0.7860380	0.9831620	1.5350500
16	H	2.2085320	-0.5130930	2.0755660
17	C	-0.1049200	1.4539620	0.3786680
18	H	1.4977290	1.7767160	1.7761390
19	H	0.1731140	0.8330010	2.4266530
20	H	1.3219610	2.3948310	-0.9138060
21	H	-0.0018020	1.6560210	-1.7804700
22	C	-1.4340230	0.6748000	0.3297270
23	H	-0.4056380	2.4781340	0.6123070
24	C	-1.4300140	-0.7163300	-0.3385680
25	H	-0.3726920	-2.4920970	-0.6160890
26	N	-2.4685660	-1.5877360	0.2388210
27	N	-2.5359280	1.4359720	-0.2779440
28	H	-1.7215340	0.5183080	1.3715830
29	H	-2.3120200	1.6555860	-1.2424750
30	H	-2.6522050	2.3222130	0.1987480
31	H	-3.3721890	-1.1593030	0.0713410
32	H	-2.3723590	-1.6025550	1.2489690
33	H	-1.7220590	-0.5681260	-1.3796920

Coordinates of conformer 1_7

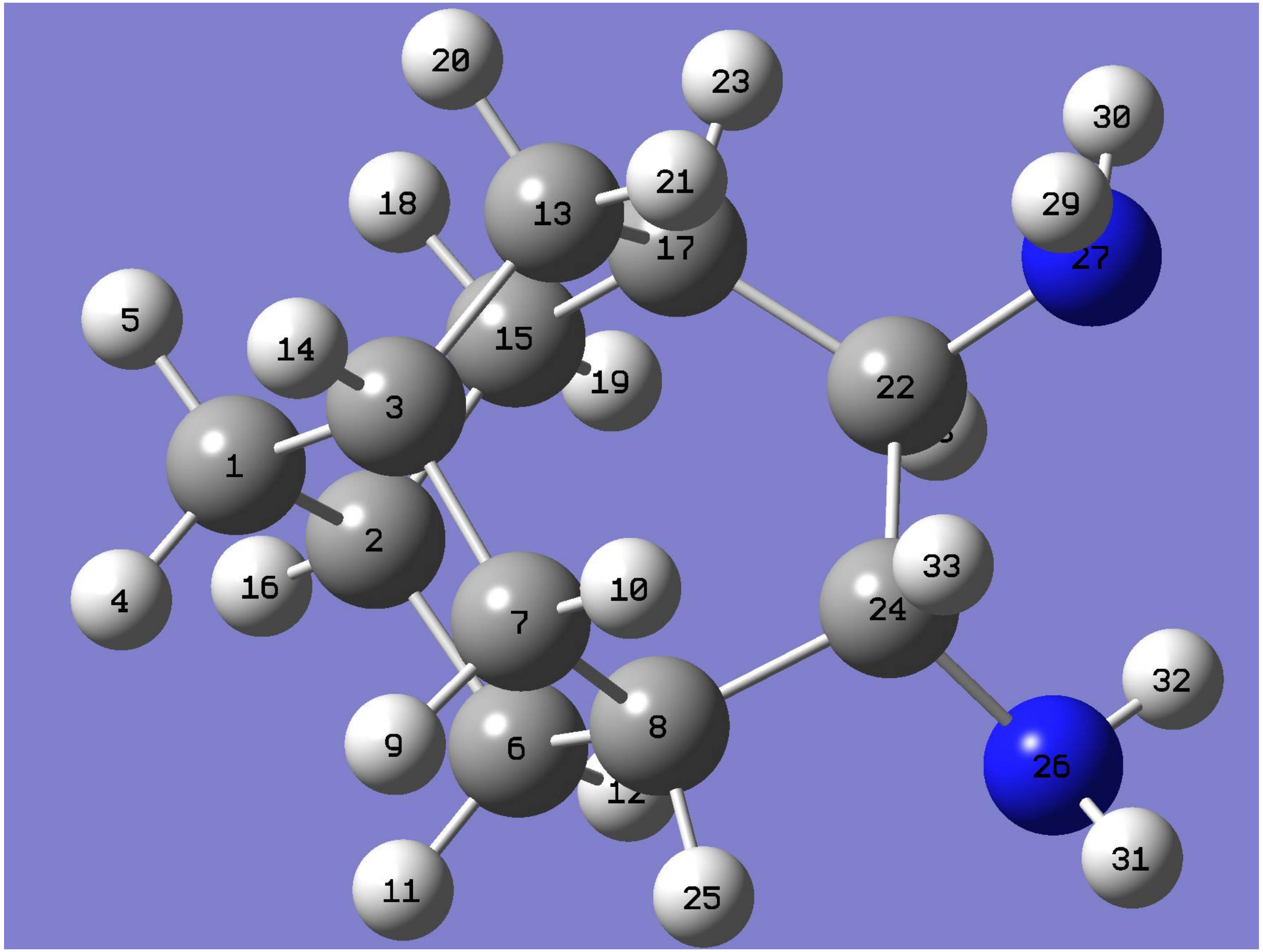

Tag	Symbol	X	Y	Z
1	C	2.4711660	-0.0158310	0.0026840
2	C	1.5881360	0.2405470	-1.2190450
3	C	1.5773740	-0.2534120	1.2203020
4	H	3.1274590	0.8374460	0.1884010
5	H	3.1110040	-0.8824540	-0.1792880
6	C	0.7054230	1.4770630	-0.9912570
7	C	0.8029120	1.0325690	1.5004810
8	C	-0.0813560	1.4767550	0.3295030
9	H	1.5271070	1.8226450	1.7138430
10	H	0.1898330	0.9209310	2.3976370
11	H	1.3572050	2.3539890	-0.9932170
12	H	0.0162090	1.6067470	-1.8281170
13	C	0.6739220	-1.4743280	0.9874120
14	H	2.2025550	-0.4681900	2.0895140
15	C	0.7911910	-1.0299110	-1.5049270
16	H	2.2219230	0.4426550	-2.0850440
17	C	-0.1063360	-1.4630190	-0.3388790
18	H	1.5006850	-1.8333600	-1.7183350
19	H	0.1825640	-0.9042090	-2.4030460
20	H	1.3097960	-2.3626560	0.9933840
21	H	-0.0164620	-1.5915630	1.8269320
22	C	-1.4369340	-0.6854540	-0.3212720
23	H	-0.4050450	-2.4944280	-0.5402420
24	C	-1.4177640	0.7179720	0.3037020
25	H	-0.3723550	2.5110040	0.5253310
26	N	-2.4455050	1.5327100	-0.3631410
27	N	-2.5417390	-1.4263910	0.3030630
28	H	-1.7169550	-0.5605030	-1.3702650
29	H	-2.3231280	-1.6190390	1.2744260
30	H	-2.6559820	-2.3247900	-0.1506450
31	H	-2.9099540	2.1468630	0.2916400
32	H	-3.1501830	0.9396310	-0.7797290
33	H	-1.6919700	0.5773480	1.3551400

Coordinates of conformer 1_8

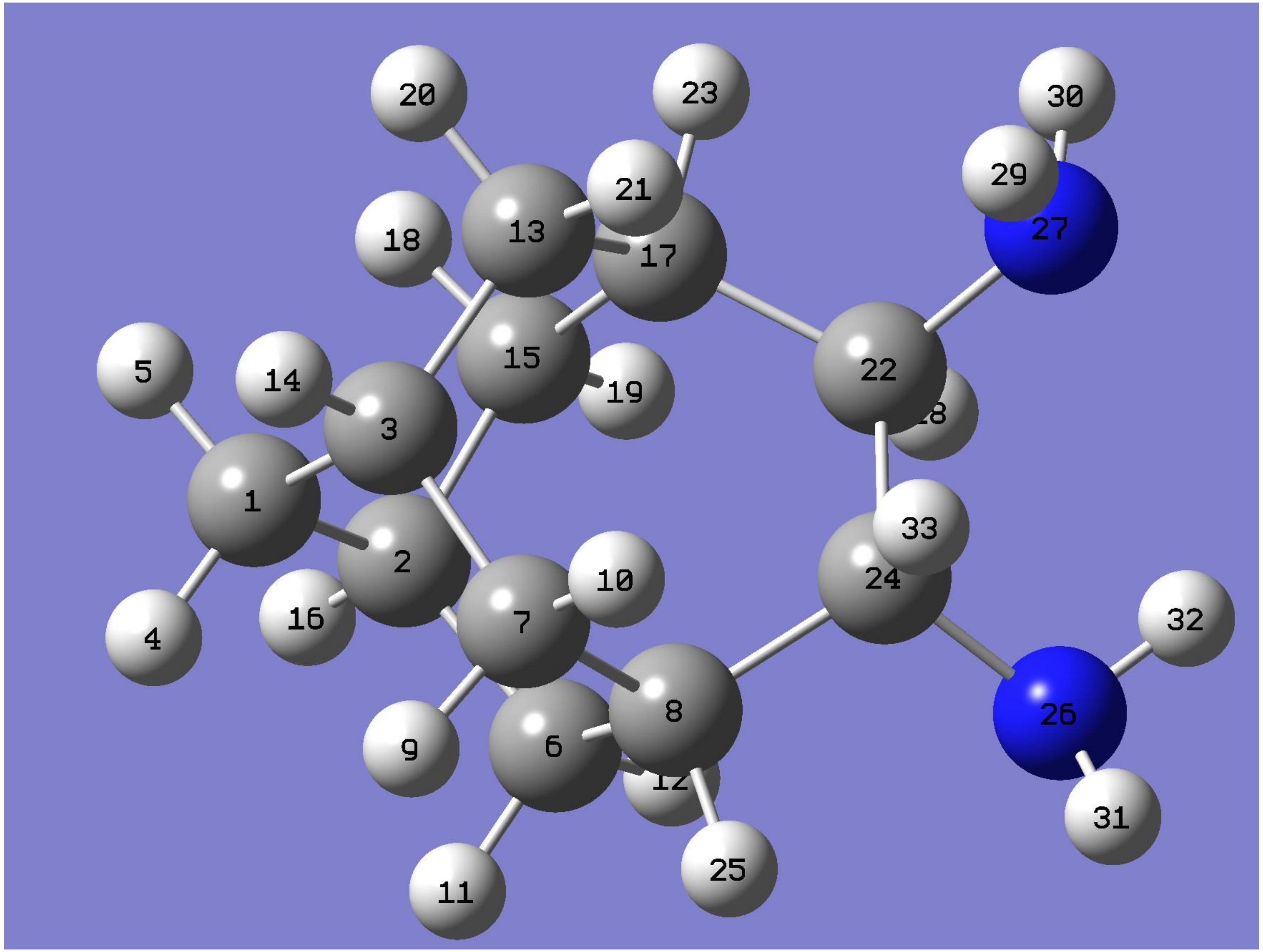

Tag	Symbol	X	Y	Z
1	C	2.4705740	-0.0183210	0.0058180
2	C	1.5893190	0.2158760	-1.2215380
3	C	1.5744840	-0.2326080	1.2260530
4	H	3.1272140	0.8376830	0.1770900
5	H	3.1099540	-0.8885770	-0.1594980
6	C	0.7036490	1.4547850	-1.0174400
7	C	0.8047570	1.0612360	1.4825600
8	C	-0.0819530	1.4813600	0.3045740
9	H	1.5320830	1.8533900	1.6766170
10	H	0.1941530	0.9705110	2.3835500
11	H	1.3535130	2.3328790	-1.0375280
12	H	0.0137020	1.5655270	-1.8561290
13	C	0.6682690	-1.4558950	1.0133570
14	H	2.1978560	-0.4339060	2.0997500
15	C	0.7957180	-1.0611540	-1.4865850
16	H	2.2241590	0.4044970	-2.0897780
17	C	-0.1071860	-1.4702590	-0.3161590
18	H	1.5074270	-1.8676820	-1.6801200
19	H	0.1912460	-0.9542300	-2.3898190
20	H	1.3025860	-2.3449700	1.0392990
21	H	-0.0255250	-1.5553120	1.8523750
22	C	-1.4341620	-0.6870220	-0.3197060
23	H	-0.4073750	-2.5048290	-0.4979880
24	C	-1.4143960	0.7176420	0.2994190
25	H	-0.3712720	2.5202610	0.4799800
26	N	-2.4430350	1.5190010	-0.3792840
27	N	-2.5510260	-1.4159910	0.2990110
28	H	-1.7057560	-0.5630090	-1.3702190
29	H	-2.3377890	-1.6150530	1.2703640
30	H	-2.6765850	-2.3110280	-0.1582260
31	H	-2.7537640	2.2772610	0.2147280
32	H	-3.2507100	0.9385630	-0.5691720
33	H	-1.6811210	0.5830980	1.3545250

Coordinates of conformer 1_9

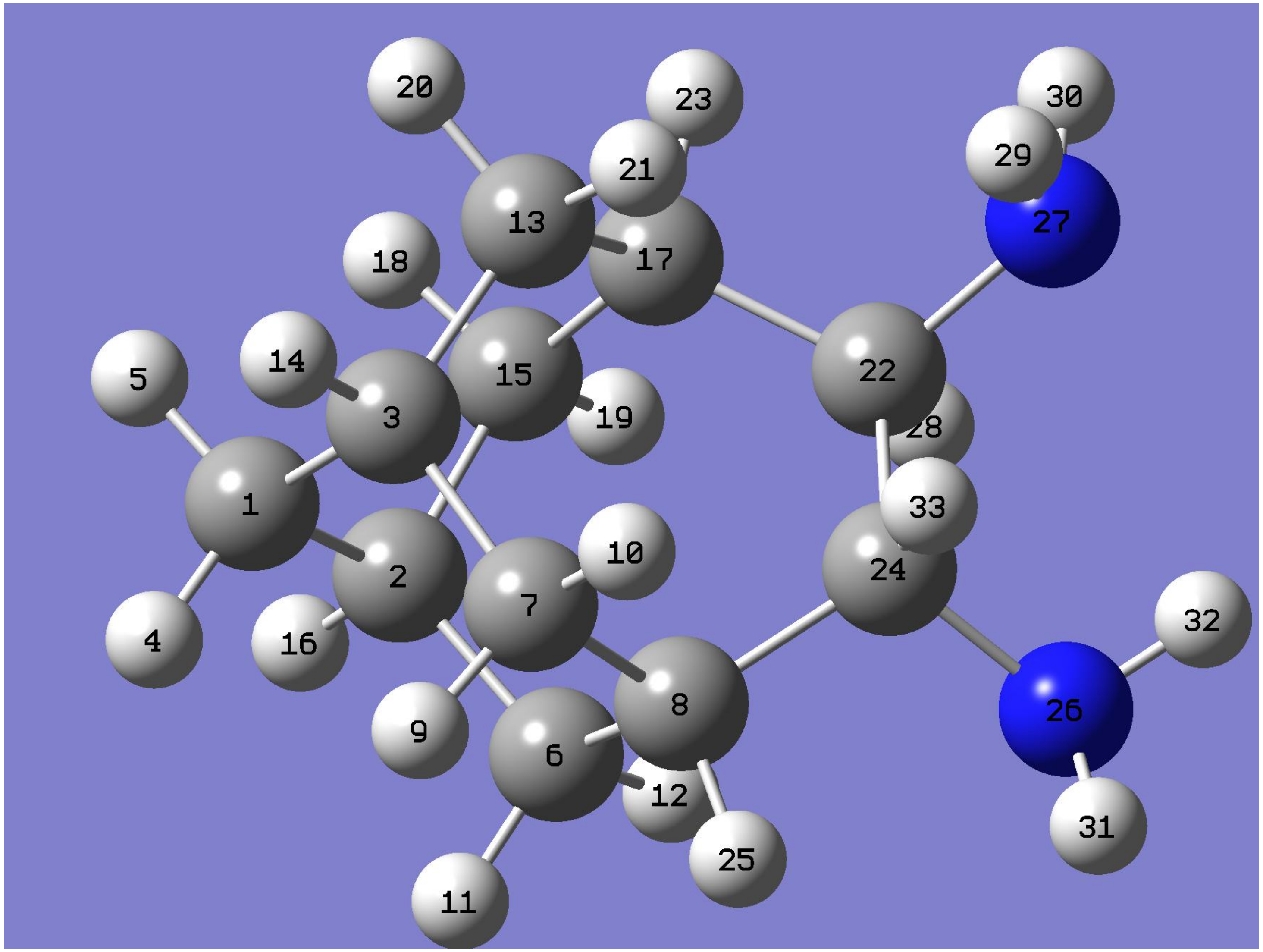

Tag	Symbol	X	Y	Z
1	C	2.4699170	-0.0112110	0.0098730
2	C	1.5900730	0.2071470	-1.2213550
3	C	1.5721140	-0.2176370	1.2302230
4	H	3.1224860	0.8491960	0.1744730
5	H	3.1133420	-0.8801830	-0.1460380
6	C	0.6965190	1.4427680	-1.0307750
7	C	0.7989880	1.0766110	1.4735400
8	C	-0.0898280	1.4811700	0.2914480
9	H	1.5244030	1.8728250	1.6578650
10	H	0.1893850	0.9945560	2.3758730
11	H	1.3412030	2.3243800	-1.0600050
12	H	0.0068570	1.5397820	-1.8712680
13	C	0.6697640	-1.4456080	1.0268370
14	H	2.1943460	-0.4090950	2.1069160
15	C	0.8045600	-1.0768270	-1.4761440
16	H	2.2255930	0.3919960	-2.0898850
17	C	-0.1017060	-1.4753360	-0.3048510
18	H	1.5212660	-1.8818970	-1.6569990
19	H	0.2035650	-0.9832180	-2.3830960
20	H	1.3067550	-2.3324080	1.0633360
21	H	-0.0263140	-1.5388740	1.8646810
22	C	-1.4286910	-0.6924630	-0.3219170
23	H	-0.4000720	-2.5121400	-0.4766710
24	C	-1.4186710	0.7117240	0.2976910
25	H	-0.3787640	2.5220990	0.4566510
26	N	-2.4486700	1.5053620	-0.3894180
27	N	-2.5507170	-1.4204800	0.2909370
28	H	-1.6945850	-0.5669900	-1.3727460
29	H	-2.3395180	-1.6256160	1.2615760
30	H	-2.6792640	-2.3128580	-0.1706730
31	H	-2.6040240	2.3718470	0.1124820
32	H	-3.3262140	0.9987240	-0.3584760
33	H	-1.6829760	0.5816650	1.3539530

Coordinates of conformer 1_10

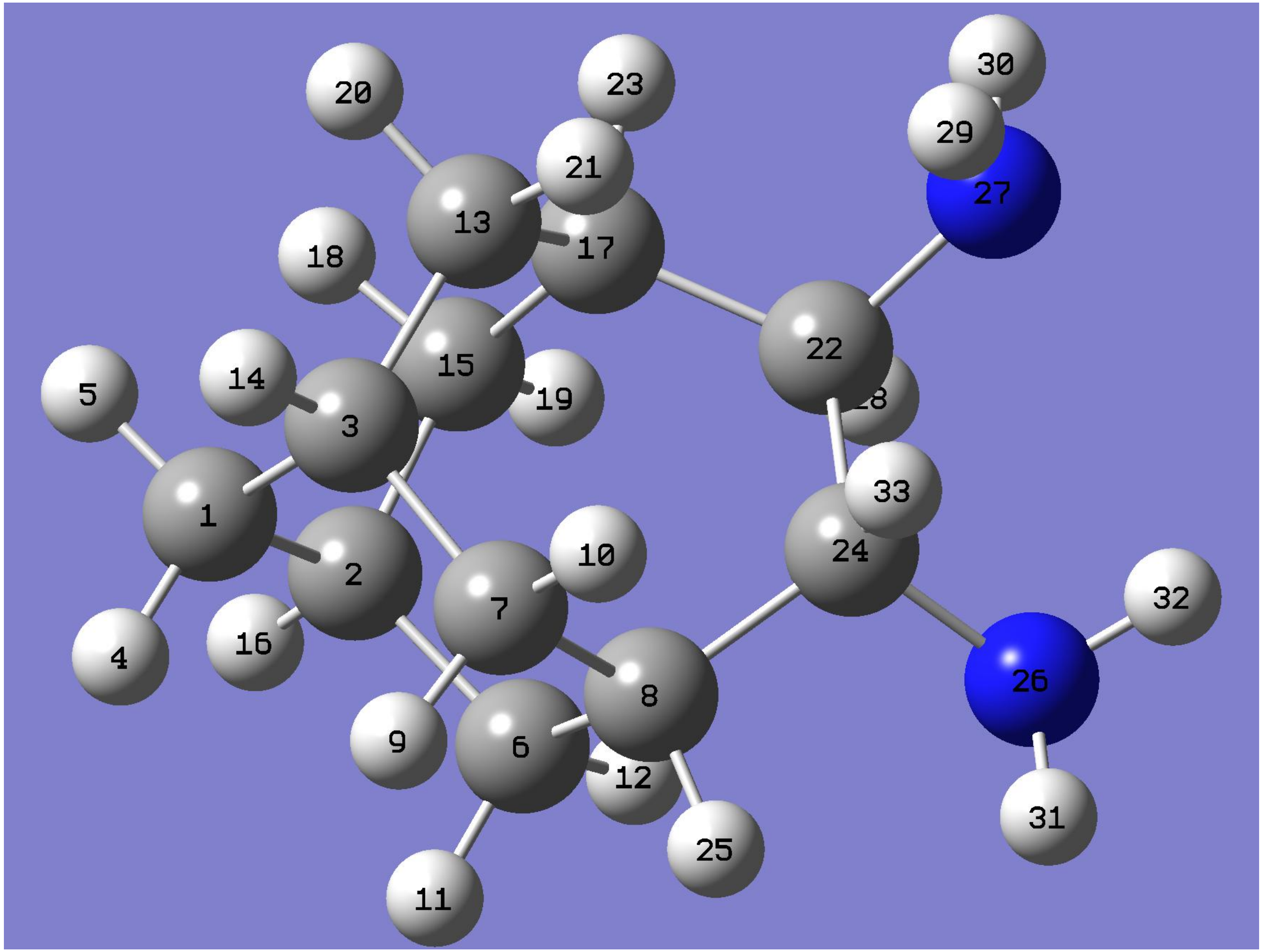

Tag	Symbol	X	Y	Z
1	C	2.4690670	0.0038550	0.0135480
2	C	1.5897480	0.2205740	-1.2183340
3	C	1.5708680	-0.2160340	1.2312860
4	H	3.1141450	0.8688670	0.1834970
5	H	3.1199830	-0.8589400	-0.1453710
6	C	0.6857170	1.4479180	-1.0239680
7	C	0.7862820	1.0700960	1.4792310
8	C	-0.1034970	1.4755110	0.2976620
9	H	1.5047380	1.8713900	1.6687090
10	H	0.1757300	0.9789480	2.3799340
11	H	1.3234360	2.3347140	-1.0476270
12	H	-0.0022780	1.5428430	-1.8660890
13	C	0.6787800	-1.4498450	1.0205410
14	H	2.1931030	-0.4059580	2.1082970
15	C	0.8146200	-1.0681270	-1.4798810
16	H	2.2252370	0.4147980	-2.0848170
17	C	-0.0911650	-1.4764820	-0.3117950
18	H	1.5375640	-1.8673360	-1.6620340
19	H	0.2147550	-0.9755150	-2.3876540
20	H	1.3224920	-2.3319840	1.0523540
21	H	-0.0172940	-1.5532040	1.8572140
22	C	-1.4226080	-0.7008680	-0.3276520
23	H	-0.3841690	-2.5139210	-0.4891870
24	C	-1.4302880	0.7010110	0.2998570
25	H	-0.3925920	2.5153330	0.4693590
26	N	-2.4616020	1.4940000	-0.3922710
27	N	-2.5398070	-1.4417460	0.2805430
28	H	-1.6886880	-0.5699860	-1.3768690
29	H	-2.3271640	-1.6492270	1.2504530
30	H	-2.6590620	-2.3338570	-0.1842360
31	H	-2.4626290	2.4356170	-0.0161220
32	H	-3.3743500	1.1158210	-0.1649100
33	H	-1.6982510	0.5701640	1.3542360

Coordinates of conformer 1_24

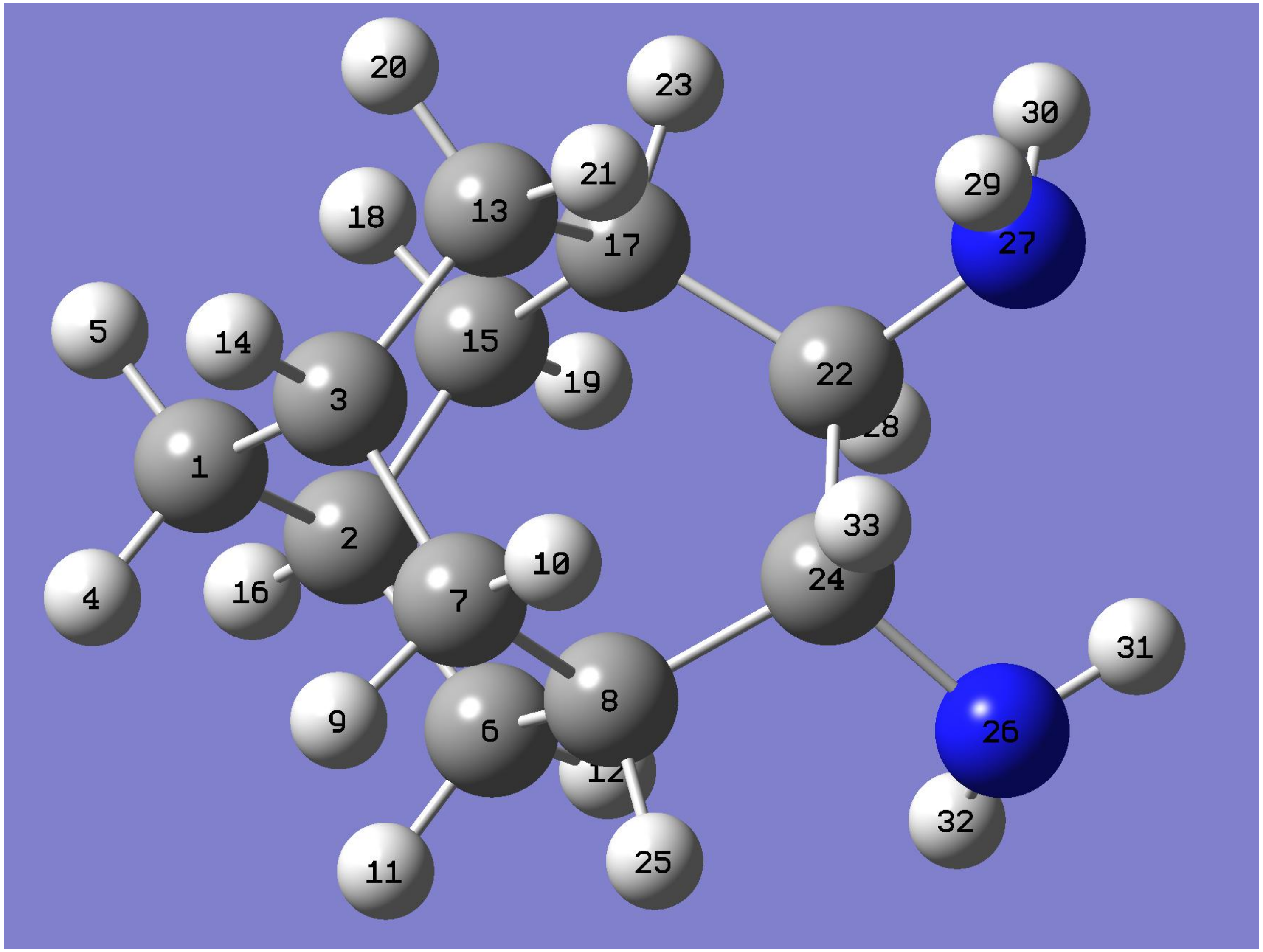

Tag	Symbol	X	Y	Z
1	C	-2.4679310	0.0216490	-0.0054460
2	C	-1.5778420	-0.2091750	-1.2269860
3	C	-1.5800950	0.2344930	1.2210630
4	H	-3.1250040	-0.8349620	0.1605520
5	H	-3.1066440	0.8913860	-0.1752510
6	C	-0.6882990	-1.4456710	-1.0172050
7	C	-0.8194190	-1.0632630	1.4820820
8	C	0.0840490	-1.4806430	0.3156260
9	H	-1.5531450	-1.8535270	1.6589690
10	H	-0.2216830	-0.9829390	2.3920140
11	H	-1.3340400	-2.3261480	-1.0479950
12	H	0.0042140	-1.5496500	-1.8565280
13	C	-0.6678140	1.4555280	1.0160250
14	H	-2.2092260	0.4374420	2.0901270
15	C	-0.7903090	1.0729000	-1.4852240
16	H	-2.2060120	-0.4015120	-2.0991790
17	C	0.1113670	1.4748750	-0.3118080
18	H	-1.5061710	1.8769330	-1.6733250
19	H	-0.1857210	0.9753570	-2.3895280
20	H	-1.2990280	2.3466440	1.0450230
21	H	0.0244750	1.5481130	1.8570480
22	C	1.4321190	0.6796910	-0.3200620
23	H	0.4156670	2.5094100	-0.4873640
24	C	1.4143540	-0.7153750	0.3313790
25	H	0.3695250	-2.5191310	0.4919760
26	N	2.4836900	-1.5694690	-0.2086920
27	N	2.5646330	1.4040580	0.2728740
28	H	1.6897480	0.5340730	-1.3712810
29	H	2.3754830	1.6001770	1.2499130
30	H	2.6850440	2.2999720	-0.1840360
31	H	3.3005360	-1.0039790	-0.4067470
32	H	2.1848450	-1.9813450	-1.0852920
33	H	1.6578010	-0.5584920	1.3849660

Coordinates of conformer 2_7

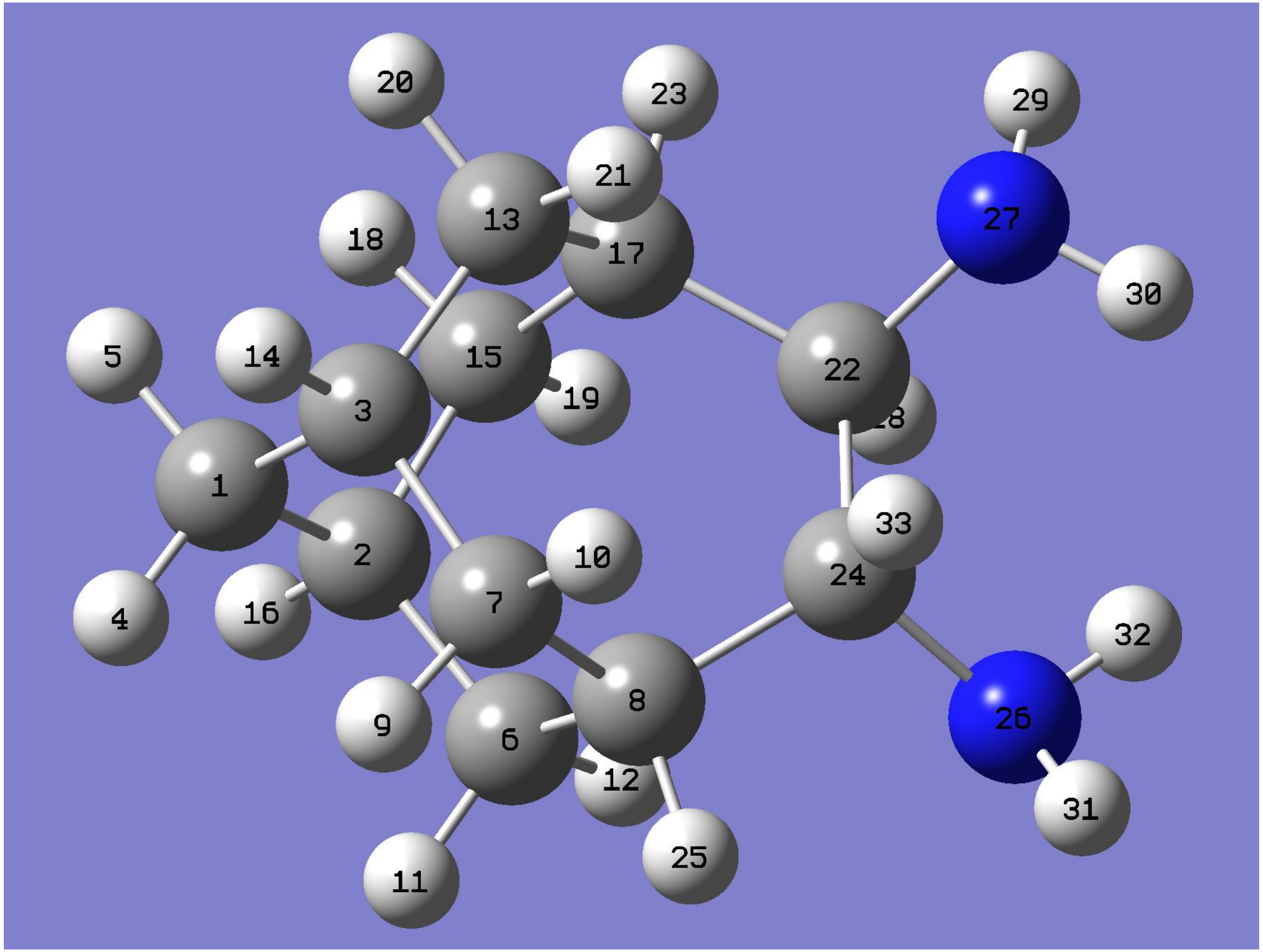

Tag	Symbol	X	Y	Z
1	C	2.4722280	0.0034210	0.0044010
2	C	1.5812410	-0.2381000	1.2232670
3	C	1.5857750	0.2402250	-1.2185990
4	H	3.1229210	-0.8563010	-0.1717730
5	H	3.1178170	0.8663810	0.1836300
6	C	0.6926270	-1.4707960	0.9976350
7	C	0.8032020	-1.0413910	-1.4970780
8	C	-0.0878940	-1.4743750	-0.3273020
9	H	1.5219240	-1.8381180	-1.7045290
10	H	0.1938190	-0.9275630	-2.3962740
11	H	1.3402020	-2.3508520	1.0068330
12	H	-0.0013750	-1.5929360	1.8314590
13	C	0.6888740	1.4675710	-0.9977160
14	H	2.2164660	0.4458330	-2.0861310
15	C	0.7914440	1.0399140	1.4982240
16	H	2.2090230	-0.4395680	2.0938750
17	C	-0.0970800	1.4694970	0.3248260
18	H	1.5058850	1.8399000	1.7077260
19	H	0.1794790	0.9243020	2.3953180
20	H	1.3307960	2.3517470	-1.0047060
21	H	-0.0021650	1.5838320	-1.8345120
22	C	-1.4251340	0.6989500	0.3053250
23	H	-0.3915790	2.5047370	0.5150850
24	C	-1.4192010	-0.7077750	-0.3094830
25	H	-0.3829790	-2.5088800	-0.5181810
26	N	-2.4371180	-1.5176100	0.3838400
27	N	-2.4329690	1.5029400	-0.4075390
28	H	-1.7101080	0.5731890	1.3565800
29	H	-2.6391200	2.3427420	0.1204140
30	H	-3.3053450	0.9928510	-0.4822500
31	H	-2.8343740	-2.2108220	-0.2363560
32	H	-3.1969520	-0.9455990	0.7279260
33	H	-1.7002190	-0.5750430	-1.3592400

Coordinates of conformer 2_8

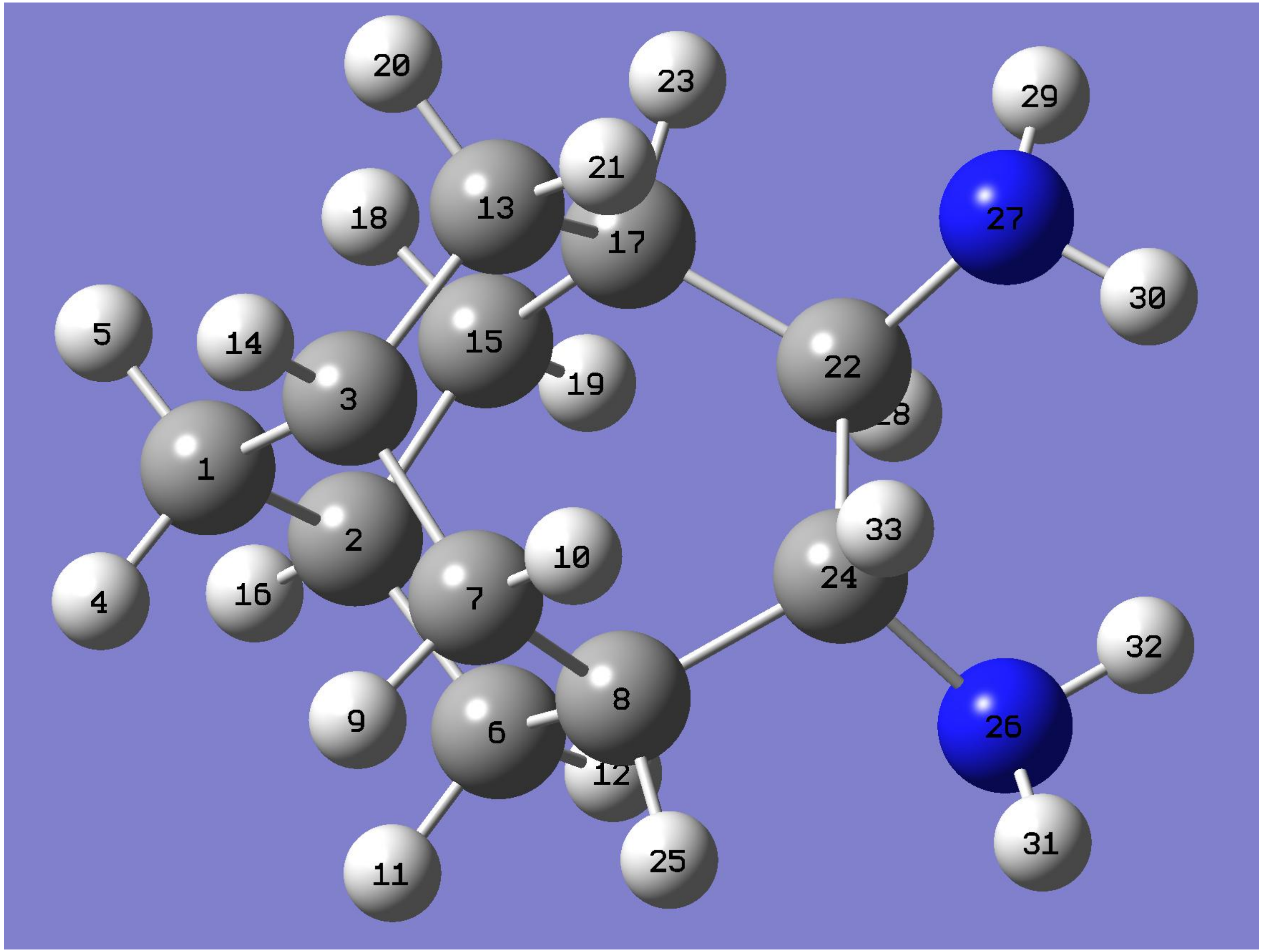

Tag	Symbol	X	Y	Z
1	C	2.4704400	0.0020390	0.0013740
2	C	1.5810940	-0.2455490	1.2201170
3	C	1.5822670	0.2474100	-1.2186590
4	H	3.1197250	-0.8575090	-0.1808030
5	H	3.1174170	0.8631330	0.1844900
6	C	0.6906200	-1.4754900	0.9887060
7	C	0.7969490	-1.0310400	-1.5038870
8	C	-0.0926890	-1.4703920	-0.3351170
9	H	1.5140360	-1.8276480	-1.7173230
10	H	0.1863400	-0.9107100	-2.4012690
11	H	1.3369820	-2.3564950	0.9905160
12	H	-0.0018170	-1.6013750	1.8229260
13	C	0.6883010	1.4750970	-0.9886710
14	H	2.2117730	0.4568040	-2.0861260
15	C	0.7923790	1.0310530	1.5042560
16	H	2.2098610	-0.4531800	2.0885450
17	C	-0.0967360	1.4689840	0.3343960
18	H	1.5074130	1.8292590	1.7185590
19	H	0.1809480	0.9094910	2.4008840
20	H	1.3324100	2.3577400	-0.9897560
21	H	-0.0031550	1.5988530	-1.8239750
22	C	-1.4260600	0.7006460	0.3101590
23	H	-0.3897610	2.5033130	0.5319270
24	C	-1.4232020	-0.7039550	-0.3116080
25	H	-0.3852470	-2.5047820	-0.5327150
26	N	-2.4275950	-1.5184810	0.3960520
27	N	-2.4296780	1.5129480	-0.4013270
28	H	-1.7149010	0.5686130	1.3586160
29	H	-2.6278440	2.3539610	0.1277110
30	H	-3.3073450	1.0120410	-0.4762610
31	H	-2.6711360	-2.3298000	-0.1591350
32	H	-3.2839160	-0.9953510	0.5335550
33	H	-1.7110470	-0.5704400	-1.3598440

Coordinates of conformer 2_9

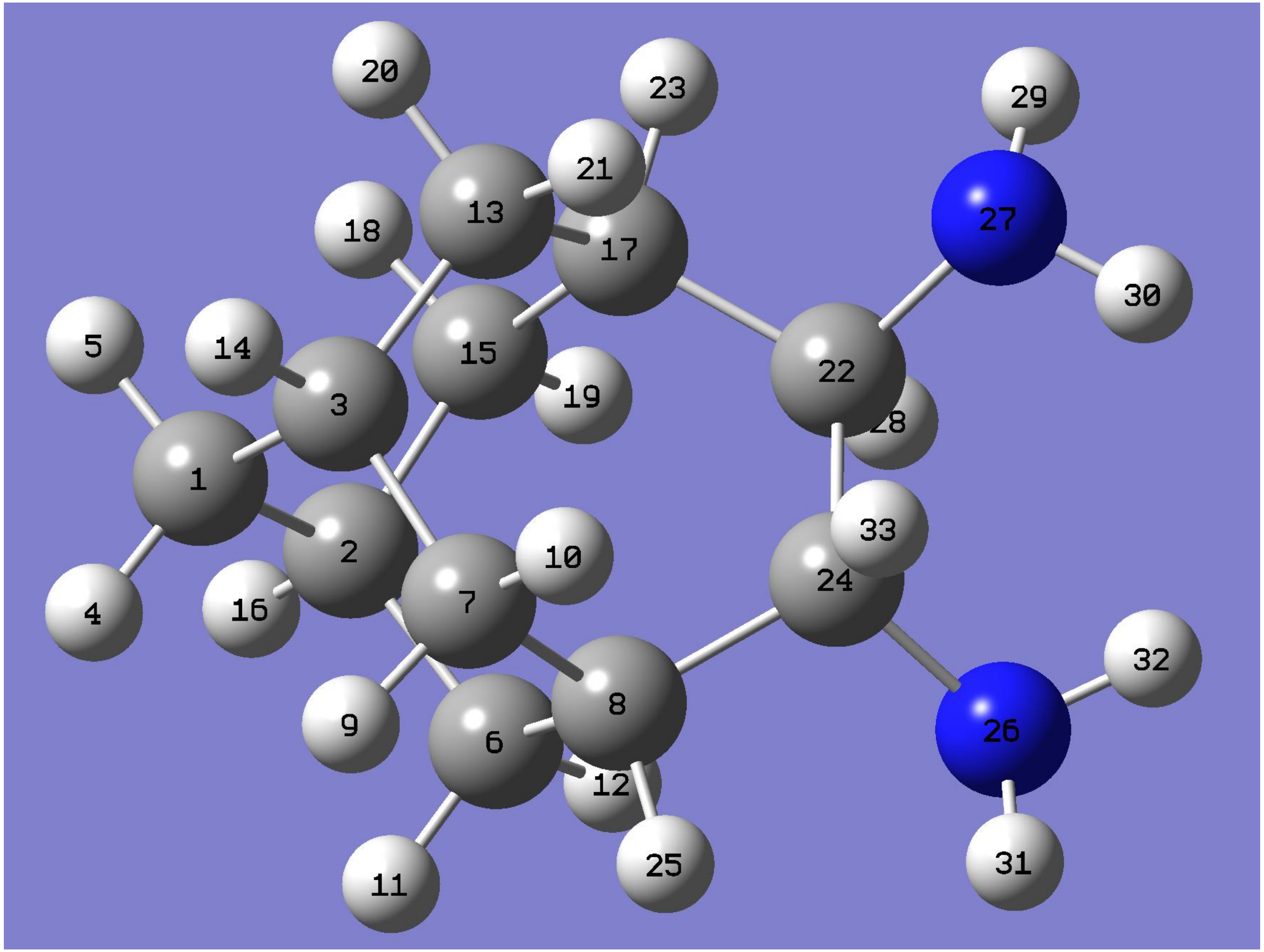

Tag	Symbol	X	Y	Z
1	C	-2.4698030	-0.0066340	0.0021110
2	C	-1.5813930	-0.2493920	-1.2181710
3	C	-1.5806870	0.2431250	1.2205060
4	H	-3.1144760	-0.8695070	0.1853080
5	H	-3.1214280	0.8512720	-0.1797370
6	C	-0.6836650	-1.4744220	-0.9890450
7	C	-0.7885640	-1.0312380	1.5039920
8	C	0.1019380	-1.4669720	0.3342840
9	H	-1.5014800	-1.8316550	1.7173950
10	H	-0.1777400	-0.9087930	2.4008740
11	H	-1.3250270	-2.3591100	-0.9899930
12	H	0.0072050	-1.5956070	-1.8253130
13	C	-0.6926920	1.4746850	0.9893090
14	H	-2.2097980	0.4485680	2.0893050
15	C	-0.7993840	1.0309620	-1.5029110
16	H	-2.2106720	-0.4599770	-2.0856120
17	C	0.0906980	1.4704340	-0.3345900
18	H	-1.5184670	1.8263820	-1.7143810
19	H	-0.1895530	0.9131090	-2.4011300
20	H	-1.3404030	2.3547680	0.9908490
21	H	-0.0008090	1.6016910	1.8239170
22	C	1.4228820	0.7065970	-0.3136330
23	H	0.3801190	2.5058860	-0.5328760
24	C	1.4312340	-0.6981810	0.3118880
25	H	0.3928980	-2.5019090	0.5329910
26	N	2.4339890	-1.5124190	-0.4016760
27	N	2.4248130	1.5277740	0.3914310
28	H	1.7095060	0.5709580	-1.3615410
29	H	2.6019610	2.3759480	-0.1340080
30	H	3.3122110	1.0416720	0.4469220
31	H	2.5191210	-2.4130640	0.0560770
32	H	3.3511320	-1.0861290	-0.3296080
33	H	1.7207110	-0.5657660	1.3598390

Coordinates of conformer 2_14

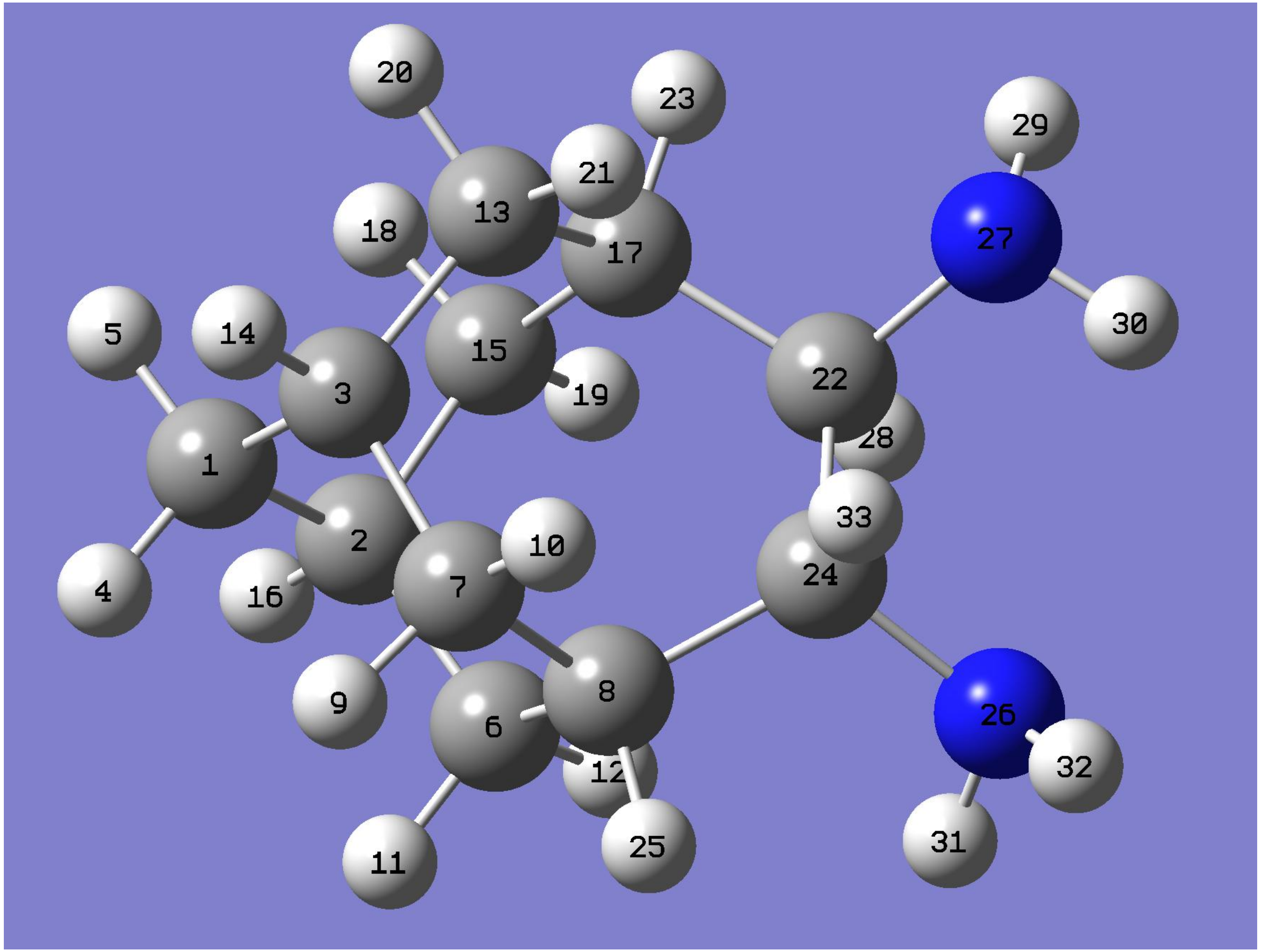

Tag	Symbol	X	Y	Z
1	C	-2.4724730	-0.0377730	0.0079170
2	C	-1.5835370	-0.2003100	-1.2255250
3	C	-1.5834240	0.1594040	1.2361330
4	H	-3.1034860	-0.9185790	0.1472460
5	H	-3.1371520	0.8182580	-0.1282510
6	C	-0.6631600	-1.4219120	-1.0625070
7	C	-0.7831150	-1.1228650	1.4497020
8	C	0.1204220	-1.4793890	0.2626080
9	H	-1.4912480	-1.9399530	1.6093110
10	H	-0.1787510	-1.0508210	2.3561510
11	H	-1.2907650	-2.3145450	-1.1156950
12	H	0.0258060	-1.4844450	-1.9087830
13	C	-0.7057140	1.4103090	1.0699050
14	H	-2.2127170	0.3139790	2.1150560
15	C	-0.8336700	1.1124420	-1.4419470
16	H	-2.2114240	-0.3786690	-2.1009350
17	C	0.0647190	1.4956190	-0.2598670
18	H	-1.5743300	1.9014030	-1.5944710
19	H	-0.2343050	1.0655310	-2.3534220
20	H	-1.3604160	2.2828120	1.1318040
21	H	-0.0079580	1.4917770	1.9055130
22	C	1.3986160	0.7366500	-0.3084150
23	H	0.3399770	2.5441260	-0.3985660
24	C	1.4344920	-0.6786910	0.2874030
25	H	0.4321460	-2.5160920	0.4047260
26	N	2.5652670	-1.4015910	-0.3242890
27	N	2.4406210	1.5219860	0.3686750
28	H	1.6434920	0.6201450	-1.3696620
29	H	2.5961380	2.3892080	-0.1318850
30	H	3.3153470	1.0106570	0.3257860
31	H	2.2504340	-2.0750170	-1.0102070
32	H	3.1057330	-1.8916230	0.3737030
33	H	1.6933230	-0.5518230	1.3394370

Coordinates of conformer 2_15

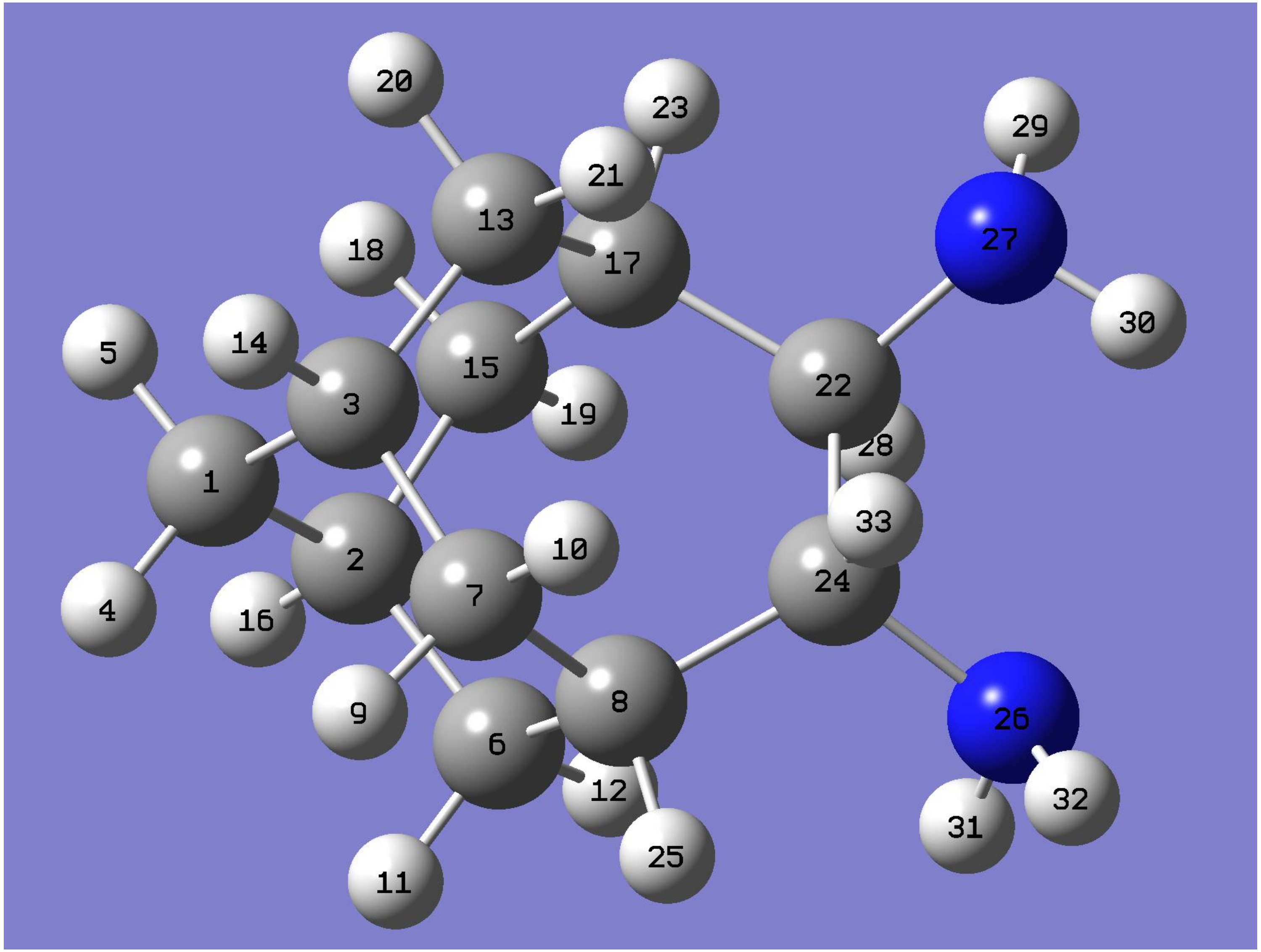

Tag	Symbol	X	Y	Z
1	C	2.4715520	-0.0293710	-0.0035010
2	C	1.5804260	-0.2124680	1.2254340
3	C	1.5848130	0.1808120	-1.2312040
4	H	3.1065680	-0.9056540	-0.1528580
5	H	3.1323780	0.8275030	0.1456880
6	C	0.6669160	-1.4361290	1.0437260
7	C	0.7880410	-1.1004610	-1.4639920
8	C	-0.1145110	-1.4772830	-0.2824960
9	H	1.4981610	-1.9130900	-1.6370100
10	H	0.1831470	-1.0154520	-2.3691120
11	H	1.2981590	-2.3267760	1.0860430
12	H	-0.0229590	-1.5137490	1.8882450
13	C	0.7031570	1.4266580	-1.0504170
14	H	2.2157490	0.3489770	-2.1064540
15	C	0.8219200	1.0926490	1.4576230
16	H	2.2070610	-0.3986830	2.1001230
17	C	-0.0725180	1.4902170	0.2772760
18	H	1.5571580	1.8834010	1.6260990
19	H	0.2184660	1.0284340	2.3654440
20	H	1.3554060	2.3019450	-1.0972210
21	H	0.0079380	1.5181890	-1.8870280
22	C	-1.4058820	0.7294940	0.3064420
23	H	-0.3513240	2.5357680	0.4303400
24	C	-1.4330830	-0.6822270	-0.2970400
25	H	-0.4232930	-2.5129770	-0.4390520
26	N	-2.5622700	-1.4033650	0.3126610
27	N	-2.4411240	1.5178470	-0.3768690
28	H	-1.6607640	0.6087140	1.3655610
29	H	-2.6116920	2.3766070	0.1331340
30	H	-3.3114680	0.9979750	-0.3572170
31	H	-2.2713630	-1.9010520	1.1452660
32	H	-2.9482160	-2.0771380	-0.3344370
33	H	-1.7003490	-0.5556570	-1.3472080

Coordinates of conformer 2_16

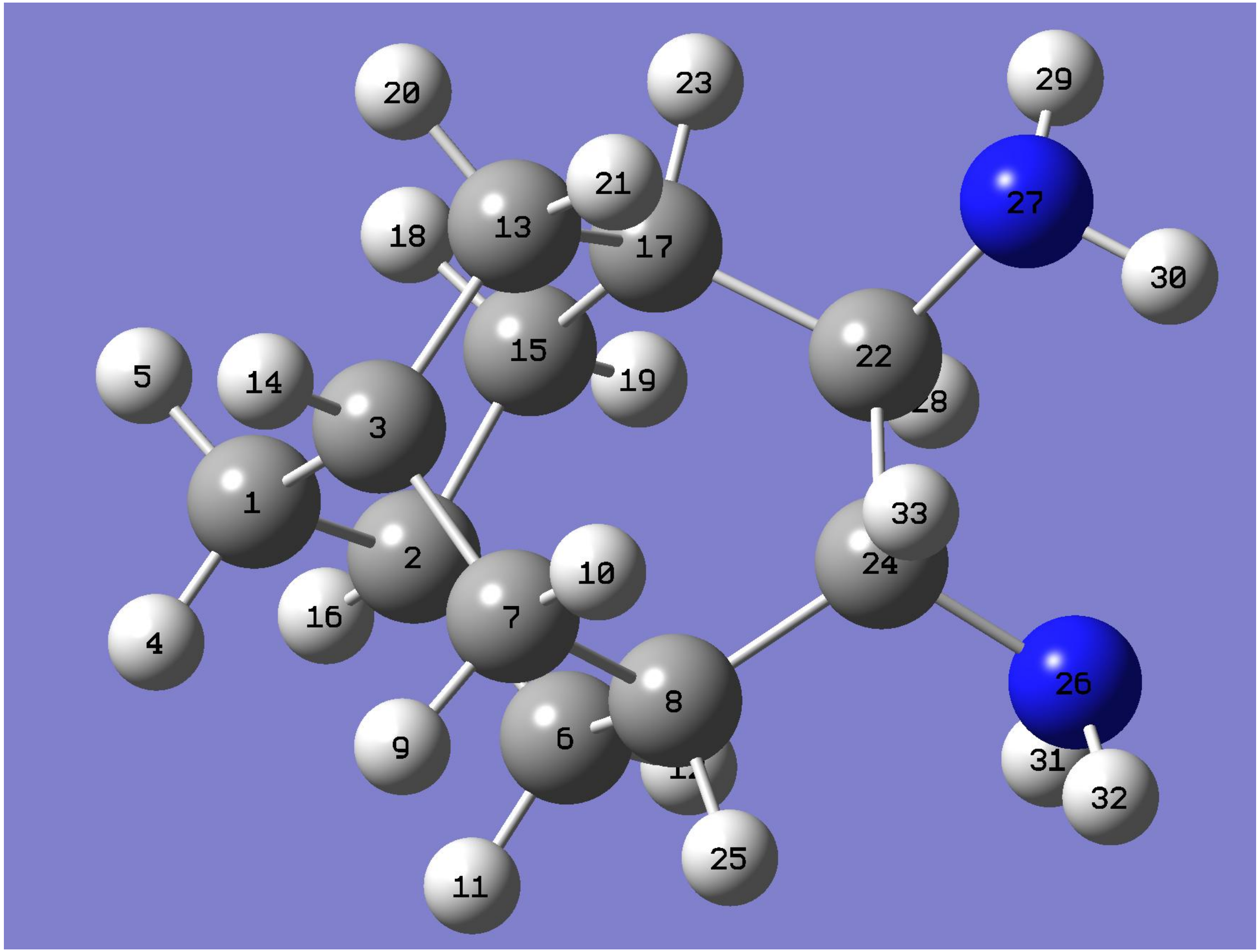

Tag	Symbol	X	Y	Z
1	C	2.4706270	-0.0190610	0.0038410
2	C	1.5758500	-0.2162230	1.2279440
3	C	1.5876670	0.1966350	-1.2256260
4	H	3.1103750	-0.8911380	-0.1498830
5	H	3.1268350	0.8397330	0.1622270
6	C	0.6687450	-1.4422600	1.0330990
7	C	0.7969650	-1.0859240	-1.4717540
8	C	-0.1072860	-1.4760070	-0.2960120
9	H	1.5106010	-1.8942090	-1.6504640
10	H	0.1939060	-0.9949750	-2.3775990
11	H	1.3032580	-2.3307630	1.0714170
12	H	-0.0245260	-1.5297190	1.8738950
13	C	0.6995720	1.4367740	-1.0382290
14	H	2.2211580	0.3747410	-2.0970540
15	C	0.8088900	1.0824980	1.4675130
16	H	2.2001270	-0.4059620	2.1035640
17	C	-0.0821050	1.4850600	0.2864030
18	H	1.5384410	1.8762950	1.6459390
19	H	0.2017360	1.0072280	2.3720820
20	H	1.3477030	2.3156030	-1.0745460
21	H	0.0073350	1.5322160	-1.8768310
22	C	-1.4130810	0.7196440	0.3010020
23	H	-0.3670880	2.5277720	0.4470630
24	C	-1.4308270	-0.6878150	-0.3111260
25	H	-0.4104820	-2.5122360	-0.4616690
26	N	-2.5564310	-1.4103280	0.3005500
27	N	-2.4448880	1.5110280	-0.3845600
28	H	-1.6740560	0.5933320	1.3584890
29	H	-2.6259450	2.3629250	0.1331380
30	H	-3.3122450	0.9862370	-0.3838330
31	H	-2.3104820	-1.7228890	1.2330820
32	H	-2.7789430	-2.2356500	-0.2415860
33	H	-1.6985660	-0.5633670	-1.3616960

Coordinates of conformer 2_17

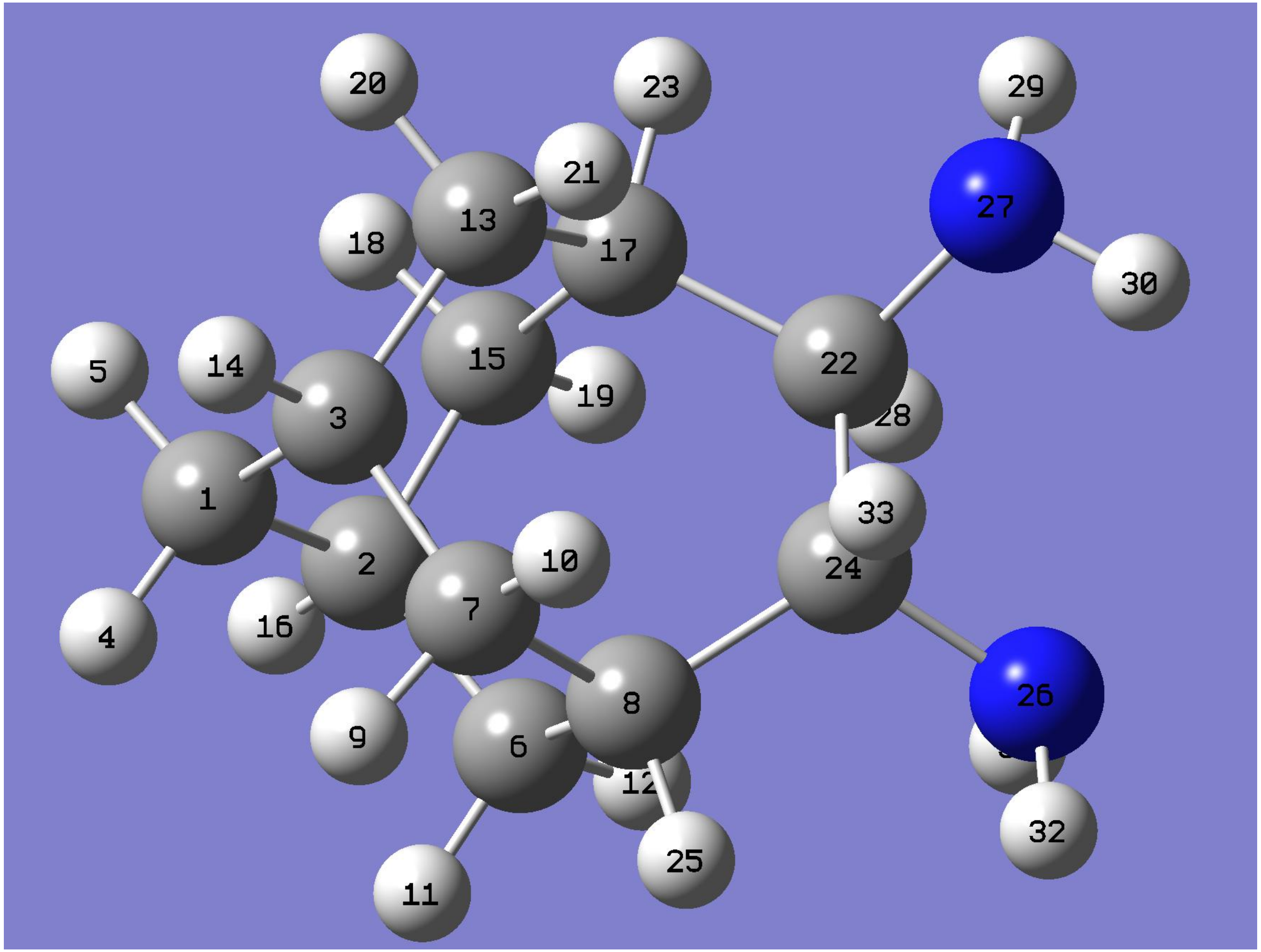

Tag	Symbol	X	Y	Z
1	C	2.4696600	-0.0094110	0.0133530
2	C	1.5697870	-0.2202060	1.2314500
3	C	1.5920480	0.2126430	-1.2188310
4	H	3.1137520	-0.8776080	-0.1442100
5	H	3.1215870	0.8507380	0.1819160
6	C	0.6683940	-1.4479120	1.0225800
7	C	0.8072790	-1.0705900	-1.4792930
8	C	-0.1002580	-1.4739250	-0.3107610
9	H	1.5243320	-1.8748590	-1.6622830
10	H	0.2073400	-0.9737920	-2.3866400
11	H	1.3056770	-2.3345600	1.0578850
12	H	-0.0297390	-1.5441640	1.8583440
13	C	0.6974600	1.4470500	-1.0258250
14	H	2.2291790	0.4005560	-2.0855590
15	C	0.7952290	1.0726740	1.4773150
16	H	2.1906010	-0.4140100	2.1086660
17	C	-0.0915470	1.4796950	0.2947050
18	H	1.5194960	1.8690540	1.6654120
19	H	0.1839650	0.9873640	2.3782690
20	H	1.3414660	2.3293100	-1.0505870
21	H	0.0093620	1.5467050	-1.8673360
22	C	-1.4197180	0.7089920	0.2949800
23	H	-0.3834810	2.5193830	0.4624840
24	C	-1.4290120	-0.6935610	-0.3282330
25	H	-0.3962920	-2.5108580	-0.4869980
26	N	-2.5485570	-1.4247950	0.2860570
27	N	-2.4489890	1.5050480	-0.3901240
28	H	-1.6851400	0.5757080	1.3508250
29	H	-2.6374430	2.3505790	0.1352420
30	H	-3.3150790	0.9788420	-0.4074340
31	H	-2.3714190	-1.5476180	1.2773980
32	H	-2.6084440	-2.3573910	-0.1056460
33	H	-1.6928170	-0.5678540	-1.3799150

Coordinates of conformer 2_18

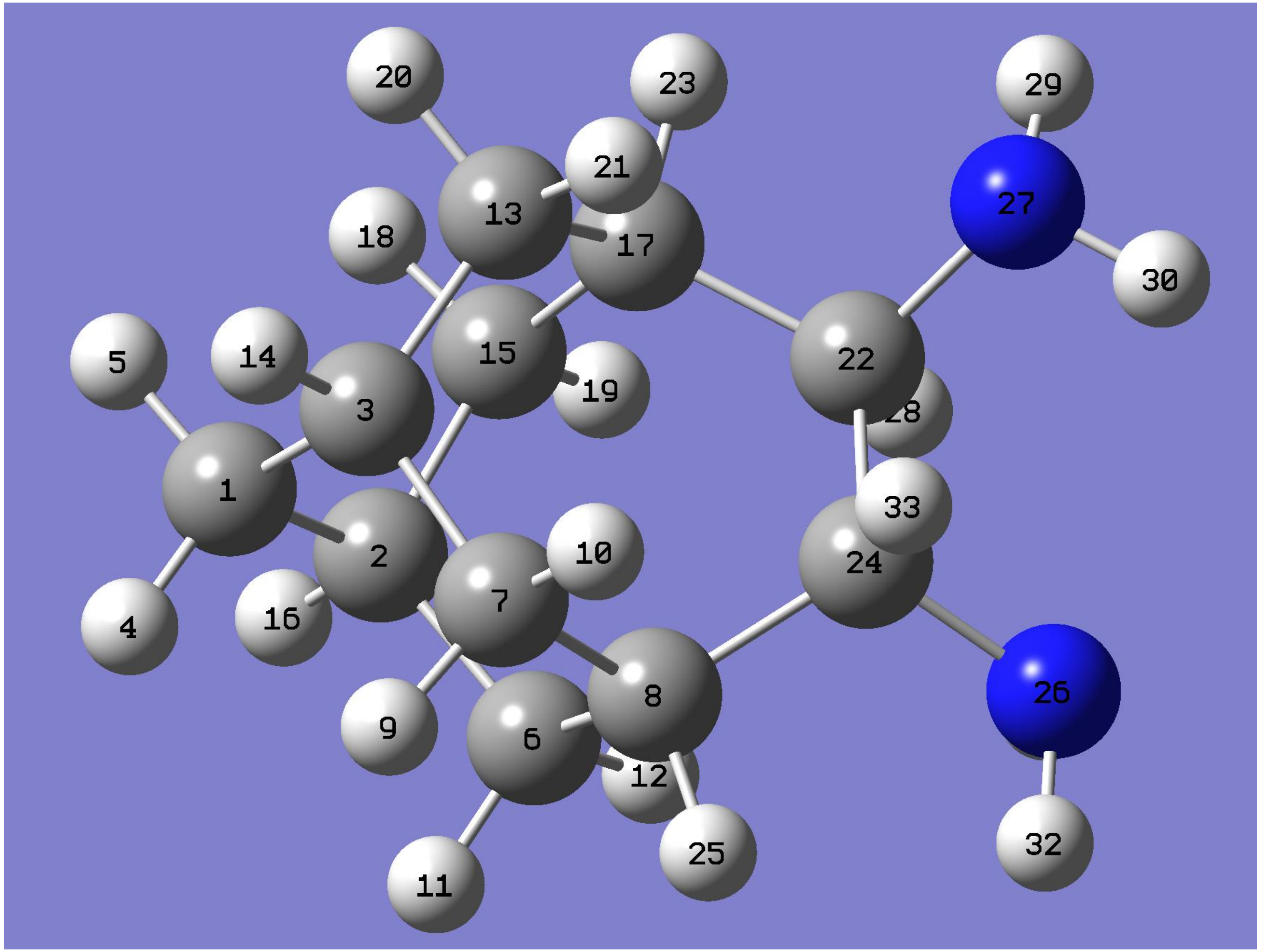

Tag	Symbol	X	Y	Z
1	C	-2.4693880	0.0016030	0.0217370
2	C	-1.5651190	0.2201710	1.2351400
3	C	-1.5963200	-0.2222290	-1.2133420
4	H	-3.1171570	0.8670360	-0.1364370
5	H	-3.1176450	-0.8601890	0.1964290
6	C	-0.6683010	1.4497540	1.0180090
7	C	-0.8168290	1.0623280	-1.4819620
8	C	0.0942120	1.4728340	-0.3188440
9	H	-1.5369720	1.8639130	-1.6647290
10	H	-0.2202100	0.9640340	-2.3913730
11	H	-1.3081610	2.3346400	1.0528530
12	H	0.0338840	1.5510550	1.8495840
13	C	-0.6961060	-1.4523130	-1.0196070
14	H	-2.2365840	-0.4156800	-2.0766340
15	C	-0.7849110	-1.0689170	1.4824120
16	H	-2.1829850	0.4151150	2.1142780
17	C	0.0985480	-1.4761450	0.2975270
18	H	-1.5052060	-1.8676840	1.6757820
19	H	-0.1704540	-0.9783450	2.3807750
20	H	-1.3365200	-2.3373860	-1.0375660
21	H	-0.0113370	-1.5528610	-1.8638120
22	C	1.4241380	-0.7007660	0.2898880
23	H	0.3965460	-2.5138390	0.4678440
24	C	1.4268990	0.6988020	-0.3412950
25	H	0.3833190	2.5109260	-0.5020210
26	N	2.5436450	1.4443800	0.2667630
27	N	2.4529550	-1.4999170	-0.3930220
28	H	1.6899480	-0.5641340	1.3454950
29	H	2.6473000	-2.3403540	0.1383650
30	H	3.3183380	-0.9736300	-0.4265650
31	H	2.4840550	1.3750050	1.2768580
32	H	2.4523700	2.4327830	0.0619370
33	H	1.6803280	0.5676210	-1.3952260

Coordinates of conformer 3_15

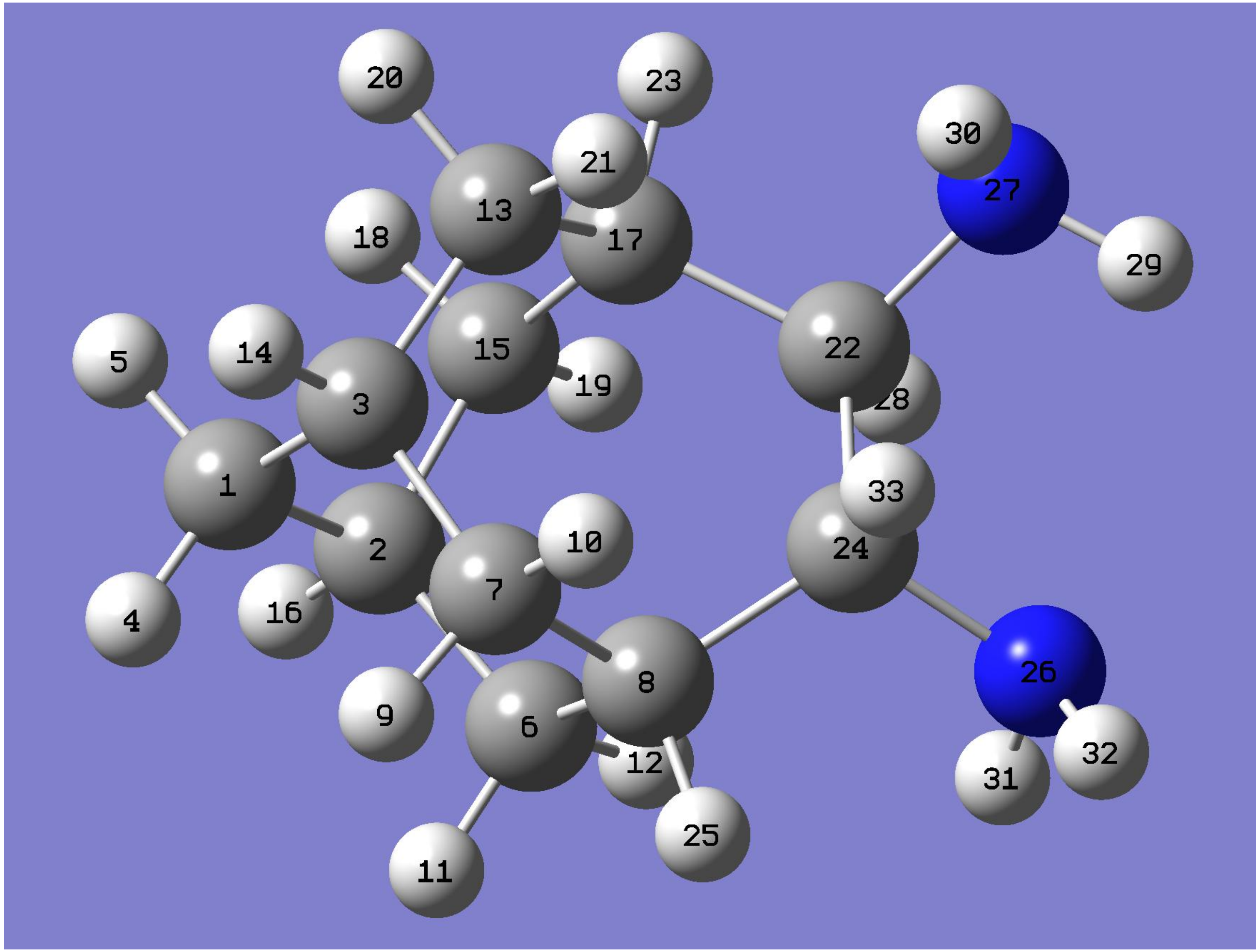

Tag	Symbol	X	Y	Z
1	C	2.4701470	0.0348450	0.0189890
2	C	1.5895200	0.2525910	-1.2118650
3	C	1.5733150	-0.2119450	1.2325350
4	H	3.1024160	0.9070360	0.2001320
5	H	3.1335410	-0.8164040	-0.1494240
6	C	0.6716030	1.4684050	-1.0038650
7	C	0.7726240	1.0613590	1.4938560
8	C	-0.1211470	1.4692130	0.3160030
9	H	1.4805370	1.8692910	1.6951250
10	H	0.1609480	0.9517400	2.3919900
11	H	1.2998740	2.3620260	-1.0162010
12	H	-0.0115630	1.5669130	-1.8516760
13	C	0.6973660	-1.4550450	1.0072580
14	H	2.1970520	-0.4039930	2.1080000
15	C	0.8355590	-1.0460160	-1.4879270
16	H	2.2236050	0.4645230	-2.0752120
17	C	-0.0685940	-1.4851470	-0.3292240
18	H	1.5742930	-1.8297750	-1.6732060
19	H	0.2395130	-0.9579580	-2.3984140
20	H	1.3522590	-2.3289480	1.0333200
21	H	0.0018980	-1.5746850	1.8420960
22	C	-1.4084020	-0.7353820	-0.3441190
23	H	-0.3417850	-2.5244560	-0.5157990
24	C	-1.4362170	0.6669860	0.2980290
25	H	-0.4346900	2.4991100	0.4995470
26	N	-2.5643560	1.3997430	-0.2977320
27	N	-2.4670760	-1.5915960	0.2116850
28	H	-1.6664040	-0.5954890	-1.3952030
29	H	-3.3401400	-1.0758560	0.1948020
30	H	-2.2718370	-1.7777060	1.1900730
31	H	-2.2726330	1.9127720	-1.1206930
32	H	-2.9534210	2.0604140	0.3608340
33	H	-1.7080860	0.5152410	1.3442180

Coordinates of conformer 3_16

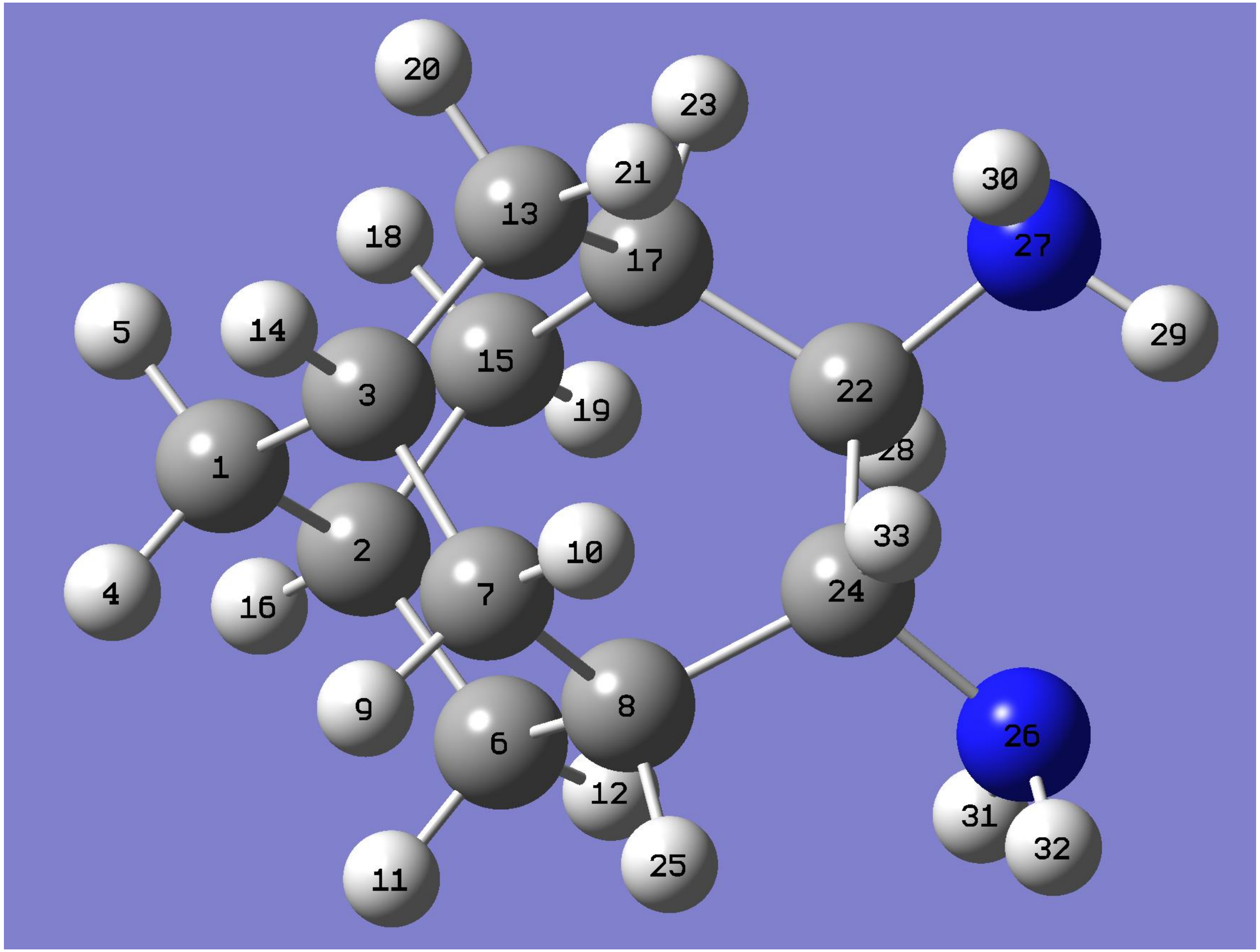

Tag	Symbol	X	Y	Z
1	C	2.4691960	0.0249230	0.0111700
2	C	1.5846800	0.2562930	-1.2143900
3	C	1.5762640	-0.2271740	1.2264730
4	H	3.1061530	0.8928460	0.1963450
5	H	3.1280570	-0.8280020	-0.1664150
6	C	0.6730100	1.4741430	-0.9932420
7	C	0.7816280	1.0471350	1.5011670
8	C	-0.1141880	1.4677970	0.3295240
9	H	1.4930880	1.8505860	1.7077000
10	H	0.1719940	0.9317440	2.4000430
11	H	1.3045580	2.3654970	-1.0017720
12	H	-0.0135960	1.5821780	-1.8371670
13	C	0.6937480	-1.4643170	0.9949610
14	H	2.2026600	-0.4290380	2.0978170
15	C	0.8224240	-1.0358710	-1.4973910
16	H	2.2162750	0.4716480	-2.0787130
17	C	-0.0785710	-1.4793830	-0.3380400
18	H	1.5556230	-1.8224770	-1.6921790
19	H	0.2228150	-0.9372920	-2.4044960
20	H	1.3445170	-2.3415360	1.0102400
21	H	0.0018470	-1.5878260	1.8321530
22	C	-1.4157580	-0.7248100	-0.3388780
23	H	-0.3579190	-2.5157290	-0.5319190
24	C	-1.4341960	0.6723870	0.3127720
25	H	-0.4221540	2.4980580	0.5220280
26	N	-2.5589470	1.4062660	-0.2844930
27	N	-2.4724620	-1.5839570	0.2174870
28	H	-1.6788370	-0.5782060	-1.3883180
29	H	-3.3406930	-1.0601930	0.2297430
30	H	-2.2600910	-1.7941600	1.1872930
31	H	-2.3127040	1.7369330	-1.2106900
32	H	-2.7849350	2.2202340	0.2731390
33	H	-1.7061970	0.5218330	1.3594460

Coordinates of conformer 3_17

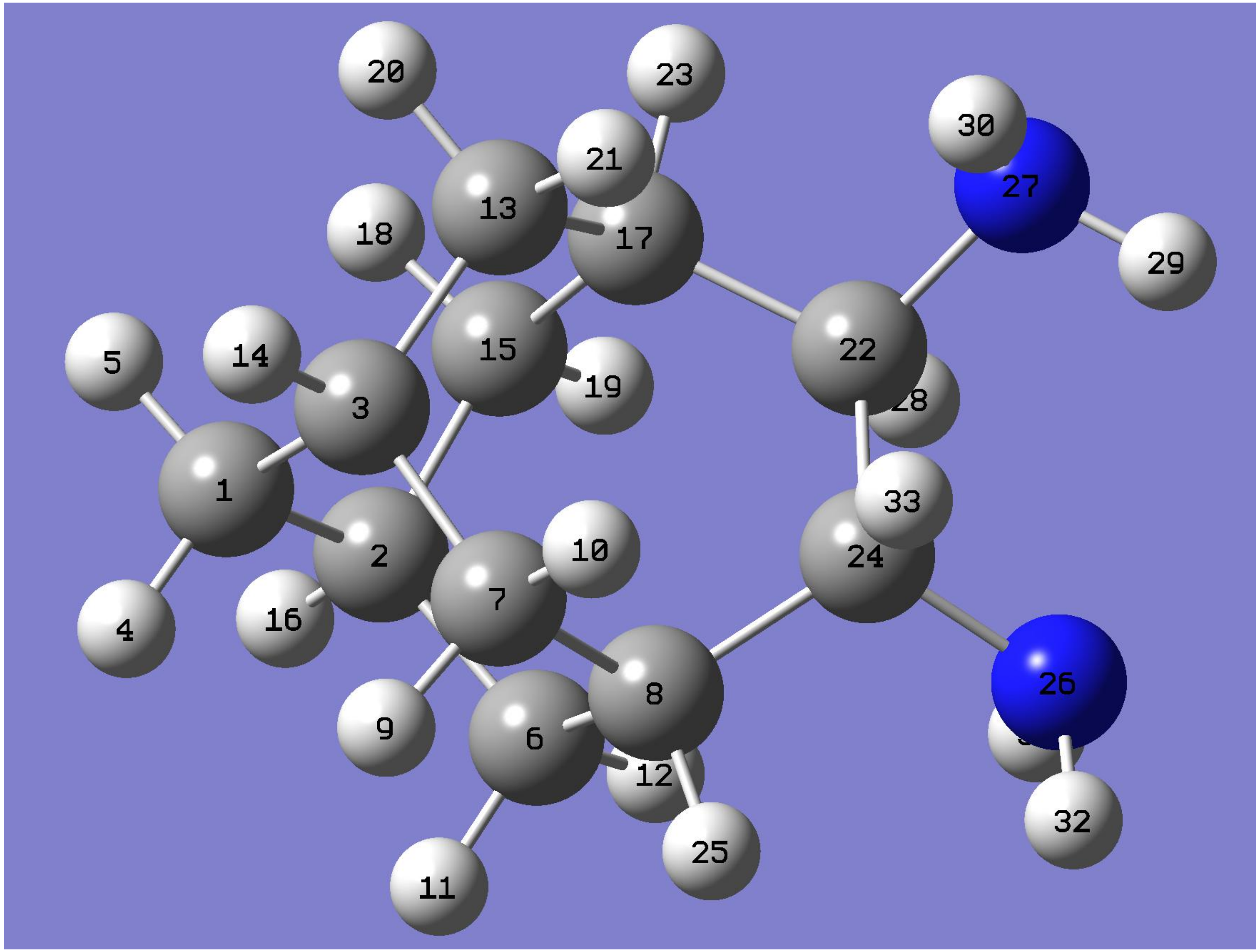

Tag	Symbol	X	Y	Z
1	C	-2.4681300	0.0149960	-0.0007960
2	C	-1.5780750	0.2640310	1.2172720
3	C	-1.5809030	-0.2471390	-1.2181550
4	H	-3.1097510	0.8781350	-0.1921630
5	H	-3.1224390	-0.8386250	0.1899220
6	C	-0.6732060	1.4831400	0.9776590
7	C	-0.7918900	1.0263890	-1.5115590
8	C	0.1069970	1.4644590	-0.3488040
9	H	-1.5066310	1.8249820	-1.7253630
10	H	-0.1851840	0.9017400	-2.4112100
11	H	-1.3084610	2.3718970	0.9794380
12	H	0.0180230	1.6039460	1.8159450
13	C	-0.6919520	-1.4776370	-0.9776280
14	H	-2.2112400	-0.4612740	-2.0837080
15	C	-0.8072010	-1.0207010	1.5101340
16	H	-2.2059580	0.4856710	2.0827150
17	C	0.0889060	-1.4725280	0.3502730
18	H	-1.5345970	-1.8093090	1.7180120
19	H	-0.2027540	-0.9087310	2.4124970
20	H	-1.3388110	-2.3578730	-0.9774020
21	H	-0.0051960	-1.6084960	-1.8178850
22	C	1.4233430	-0.7131820	0.3334660
23	H	0.3751710	-2.5049770	0.5546390
24	C	1.4326900	0.6778010	-0.3311870
25	H	0.4074890	2.4945400	-0.5555260
26	N	2.5497870	1.4223650	0.2688490
27	N	2.4774050	-1.5776460	-0.2217660
28	H	1.6923670	-0.5579050	1.3804630
29	H	3.3440760	-1.0526000	-0.2568900
30	H	2.2522620	-1.8076890	-1.1840340
31	H	2.3725150	1.5650320	1.2574420
32	H	2.6128740	2.3463710	-0.1421820
33	H	1.7024330	0.5243550	-1.3783300

References

1. Schlatmann, J. L. M. A.; Korsloot, J. G.; Schut, J. *Tetrahedron*, **1970**, 26, 949–954.
2. Nordlander, J. E.; Wu, F. Y. H.; Jindal, S. P.; Hamilton, J. B. *J. Am. Chem. Soc.*, **1969**, 91, 3962–3964.
3. Balode, D. E.; Ziemelis, K. M. USSR Patent SU 637404 A1, **1978**.
4. Stetter, H.; Löhr, V.; Simos, A. *Liebigs Ann. Chem.*, **1977**, 1977, 999–1004.
5. Sasaki, T.; Eguchi, S.; Hattori, S. *Heterocycles*, **1978**, 11, 235–242.
6. Avogadro: an open-source molecular builder and visualization tool. Version 1.21. http://avogadro.cc/
7. Frisch, M. J.; Trucks, G. W.; Schlegel, H. B.; Scuseria, G. E.; Robb, M. A.; Cheeseman, J. R.; Scalmani, G.; Barone, V.; Petersson, G. A.; Nakatsuji, H.; Li, X.; Caricato, M.; Marenich, A. V.; Bloino, J.; Janesko, B. G.; Gomperts, R.; Mennucci, B.; Hratchian, H. P.; Ortiz, J. V.; Izmaylov, A. F.; Sonnenberg, J. L.; Williams-Young, D.; Ding, F.; Lipparini, F.; Egidi, F.; Goings, J.; Peng, B.; Petrone, A.; Henderson, T.; Ranasinghe, D.; Zakrzewski, V. G.; Gao, J.; Rega, N.; Zheng, G.; Liang, W.; Hada, M.; Ehara, M.; Toyota, K.; Fukuda, R.; Hasegawa, J.; Ishida, T.; Nakajima, Y.; Honda, Y.; Kitao, O.; Nakai, H.; Vreven, T.; Throssell, K.; Montgomery, J. A., Jr.; Peralta, J. E.; Ogliaro, F.; Bearpark, M. J.; Heyd, J. J.; Brothers, E. N.; Kudin, K. N.; Staroverov, V. N.; Keith, T. A.; Kobayashi, R.; Normand, J.; Raghavachari, K.; Rendell, A. P.; Burant, J. C.; Iyengar, S. S.; Tomasi, J.; Cossi, M.; Millam, J. M.; Klene, M.; Adamo, C.; Cammi, R.; Ochterski, J. W.; Martin, R. L.; Morokuma, O.; Farkas, J. B.; Foresman, J. B.; Fox, D. J. *Gaussian 09*, Revision A.02; Gaussian, Inc.: Wallingford CT, **2016**.
